# Supplementary material for: The NFIB‐ERO1A axis promotes breast cancer metastatic colonization of disseminated tumour cells
Source: EMBO Mol Med. 2021 Mar 10;13(4):e13162. doi: 10.15252/emmm.202013162 (PMC8033524; doi:10.15252/emmm.202013162)
Supplement: Supplementary file 1 — Appendix [file EMMM-13-e13162-s011.pdf]

The NFIB-ERO1A axis promotes breast cancer metastatic colonization of disseminated tumour cells

Federica Zilli*, Pedro Marques Ramos*, Priska Auf der Maur, Charly Jehanno, Atul Sethi, Marie-May Coissieux, Tobias Eichlisberger, Loïc Sauteur, Adelin Rouchon, Laura Bonapace, Joana Pinto Couto, Roland Rad, Michael Rugaard Jensen, Andrea Banfi, Michael B. Stadler and Mohamed Bentires-Alj

APPENDIX

<u>Table of content</u>

Appendix Figures

Appendix Figure S1 *Foxp1* depletion delays mammary tumour onset and decreases metastasis.

Appendix Figure S2 *Nfib* ablation in a highly metastatic mammary cancer line increases overall survival in mice.

Appendix Figure S3 Overexpression of NFIB enhances proliferation, tumoursphere formation, invasion and migration.

Appendix Figure S4 *Nfib* increases *Ero1l/ERO1A* levels.

Appendix Figure S5 The NFIB-ERO1A axis promotes lung metastatic colonization.

Appendix Figure S6 NFIB-ERO1A enhances VEGFA levels.

Appendix Figure S7 Elevated *NFIB* copy number and mRNA expression correlate with poor prognosis in TNBC patients.

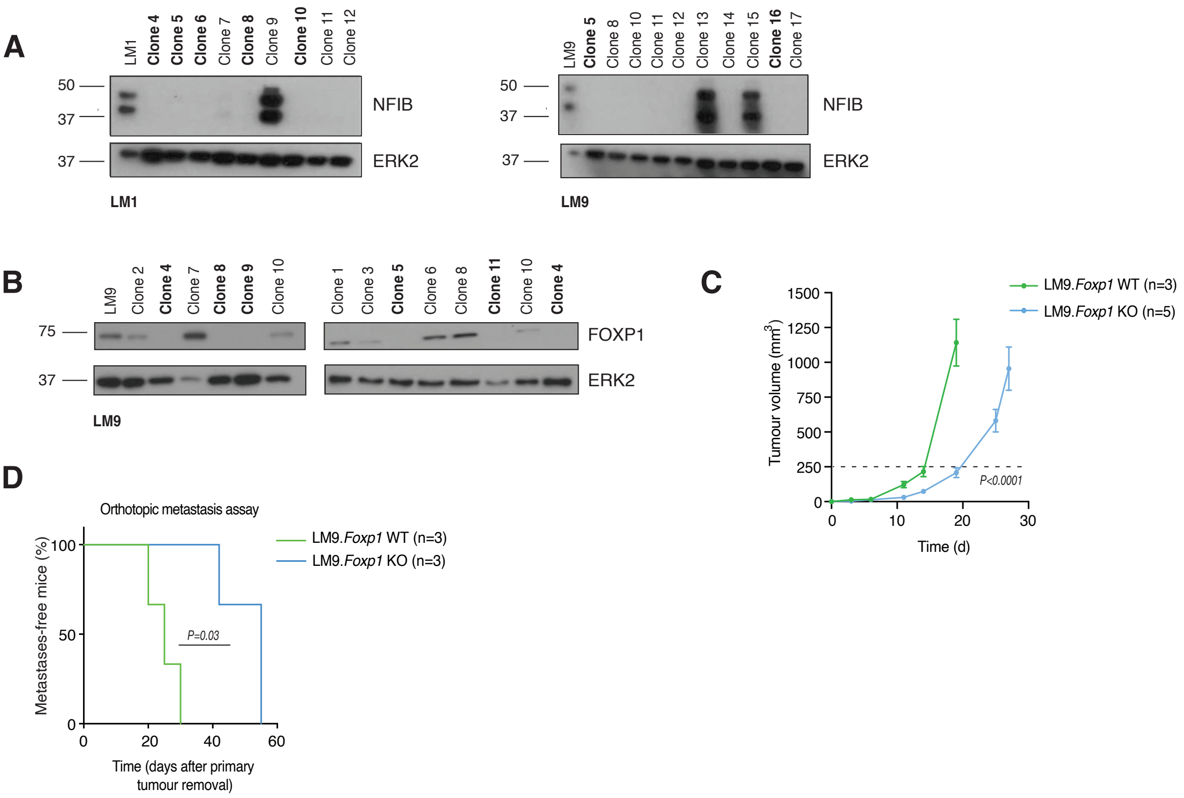

Appendix Figure S1. *Foxp1* depletion delays mammary tumour onset and decreases metastasis.

A Immunoblots showing protein levels of NFIB in single-cell-derived clones of LM1 and LM9 after *Nfib* knockout (KO). Single-cell clones were pooled for the KOs (LM1 KOs clones: 4, 5, 6, 8, 10; LM9 KOs clones: 5,16; in bold). ERK2 served as a loading control.

B Immunoblots showing protein levels of FOXP1 in single-cell-derived clones of LM9 after *Foxp1* knockout. Single-cell clones were pooled (LM9 WT clones: 1,2,3,6,7,10; KOs: 4,5,8,9,11; in bold). ERK2 served as a loading control.

C Graph representing the kinetics of LM9 *Foxp1* WT ($n = 3$) or KO ($n = 5$) tumour growth. Curves show means of tumour volume ± s.d., two-tailed Student's *t*-test on the times to reach 250 mm^3 (dashed line).

D Kaplan-Meier plot depicting metastasis onset after tumour removal in mice injected orthotopically with LM9 *Foxp1* WT ($n = 3$) or KO ($n = 3$), two-tailed log-rank test.

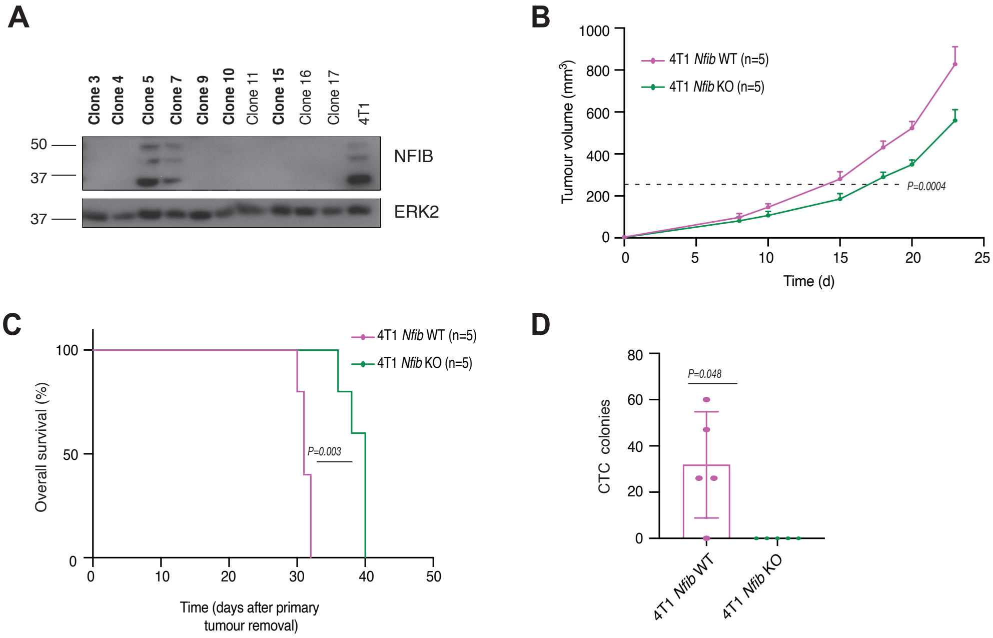

Appendix Figure S2. Nfib ablation in a highly metastatic mammary cancer line increases overall survival in mice.

A Immunoblots showing protein levels of NFIB in single-cell-derived clones of 4T1 after *Nfib* knockout. Single-cell clones were pooled: WT clones: 5,7; KOs clones: 3, 4, 9, 10, 15; in bold. ERK2 served as a loading control.

B Graph representing the kinetics of 4T1 *Nfib* WT (n = 5) and 4T1 *Nfib* KO (n = 5) tumour growth upon orthotopic injection of 250 x 10^3 cells into BALB/c animals. Curves show means of tumour volume ± s.d., two-tailed Student's t-test on the times to reach 250 mm^3 (dashed line).

C Kaplan-Meier plot depicting survival analysis after tumour removal from mice injected orthotopically with 4T1 *Nfib* WT (n = 5) or 4T1 *Nfib* KO (n = 5) cells, two-tailed log-rank test.

D Quantification of CTC colonies from mice orthotopically injected with 4T1 *Nfib* WT (n = 5) and 4T1 *Nfib* KO (n = 5). Means ± s.d., two-tailed Mann-Whitney U-test.

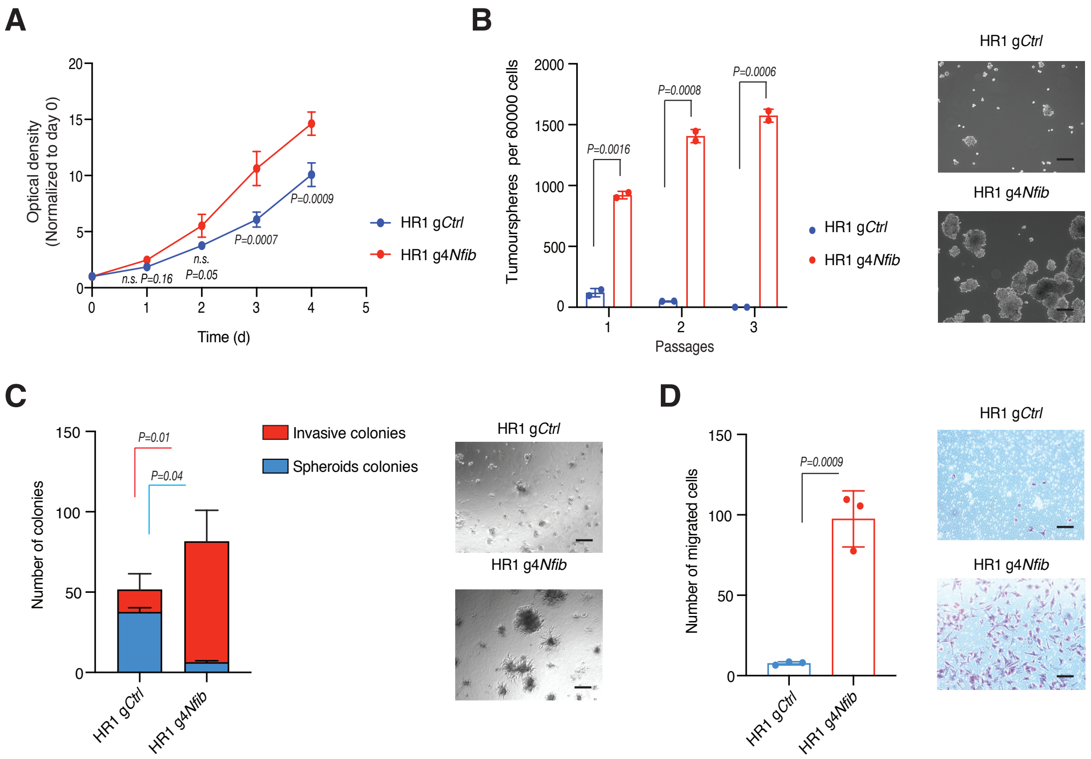

Appendix Figure S3. Overexpression of NFIB enhances proliferation, tumoursphere formation, invasion and migration.

A Growth curve depicting *in vitro* proliferation of HR1 g*Ctrl* and HR1 g4*Nfib* cells. $n = 2$ biological replicates and $n = 3$ technical replicates, means ± s.d., two-tailed Student's t-test. The average of the triplicates of each cell line was normalized to the average of day zero.

B Left panel: Bar graph showing tumoursphere numbers over three passages of HR1 g*Ctrl* and HR1 g4*Nfib*, $n = 2$ biological replicates and $n = 3$ technical replicates, means ± s.d., two-tailed Student's t-test. Right panel: Representative images of tumourspheres of HR1 g*Ctrl* and HR1 g4*Nfib* cells at the first passage. Scale bar 100 µm.

C Left panel: Bar graph showing the number of HR1 g*Ctrl* and HR1 g4*Nfib* invasive and spheroid colonies. $n = 2$ biological replicates and $n = 2$ technical replicates, means ± s.d., two-tailed Student's t-test. Right panel: Representative images of HR1 g*Ctrl* and HR1 g4*Nfib* invasive and spheroid cells. Scale bar 100 µm.

D Left panel: Bar graph showing the number of HR1 g*Ctrl* and HR1 g4*Nfib* cells that migrated in 24 h. $n = 3$ biological replicates and $n = 2$ technical replicates, means ± s.d., two-tailed Student's t-test. Right panel: Representative images of HR1 g*Ctrl* and HR1 g4*Nfib* migrated cells stained with Crystal Violet. Scale bar 100 µm.

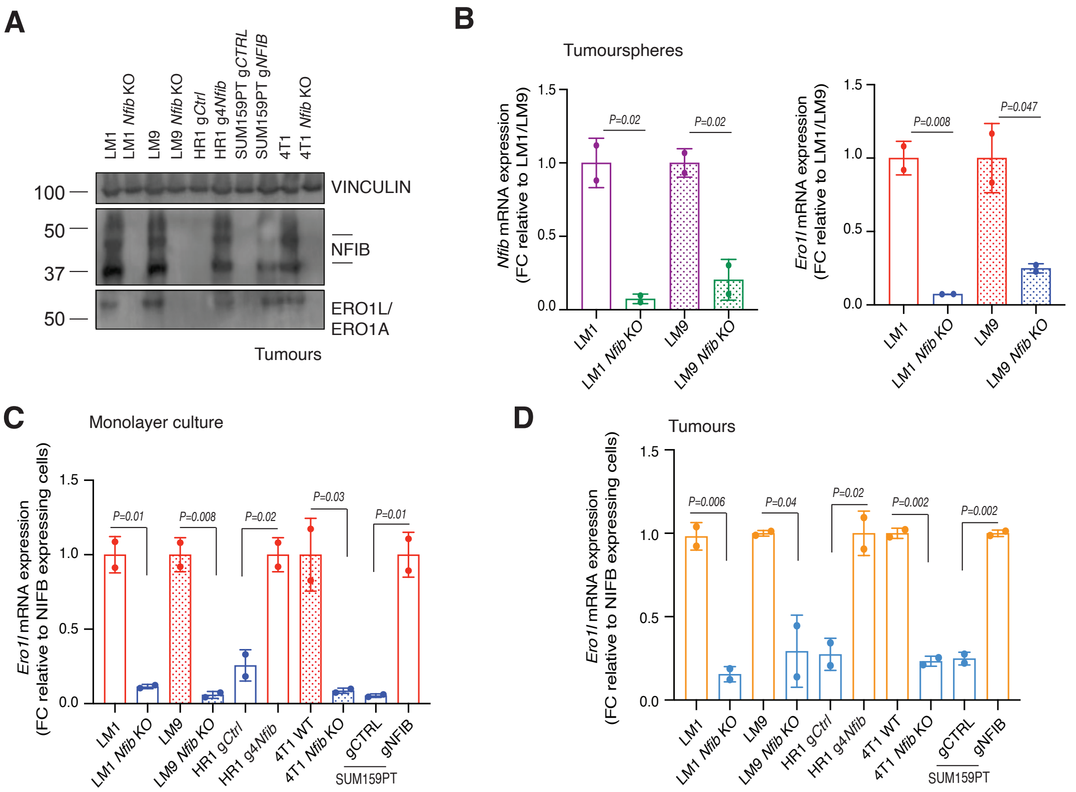

Appendix Figure S4. *Nfib* increases *Ero1l/ERO1A* levels.

A Immunoblots showing proteins levels of NFIB and ERO1L/ERO1A in tumours derived from mammary fat pad injection of LM, LM *Nfib* KO, HR1g*Ctrl*, HR1 g4*Nfib,* SUM159PT g*CTRL*, SUM159PT, 4T1 or 4T1 *Nfib* KO cell lines. VINCULIN served as a loading control.

B Bar graphs representing mean *Nfib* and *Ero1l* mRNA expression in tumourspheres derived from LM and LM KO cancer cells at the first passage. *Ero1l* is downregulated upon *Nfib* KO in this model. In both graphs, $n = 2$ biological replicates and $n = 2$ technical replicates, means ± s.d., two-tailed Student's t-test, FC= fold change.

C Bar graph representing mean Ero1l/*ERO1A* mRNA expression. Means ± s.d., $n = 2$ biological replicates and $n = 2$ technical replicates, two-tailed Student's t-test, FC= fold change.

D Bar graph representing mean *Ero1l/ERO1A* mRNA expression. Means ± s.d., $n = 2$ biological replicates and $n = 2$ technical replicates, two-tailed Student's t-test, FC= fold change.

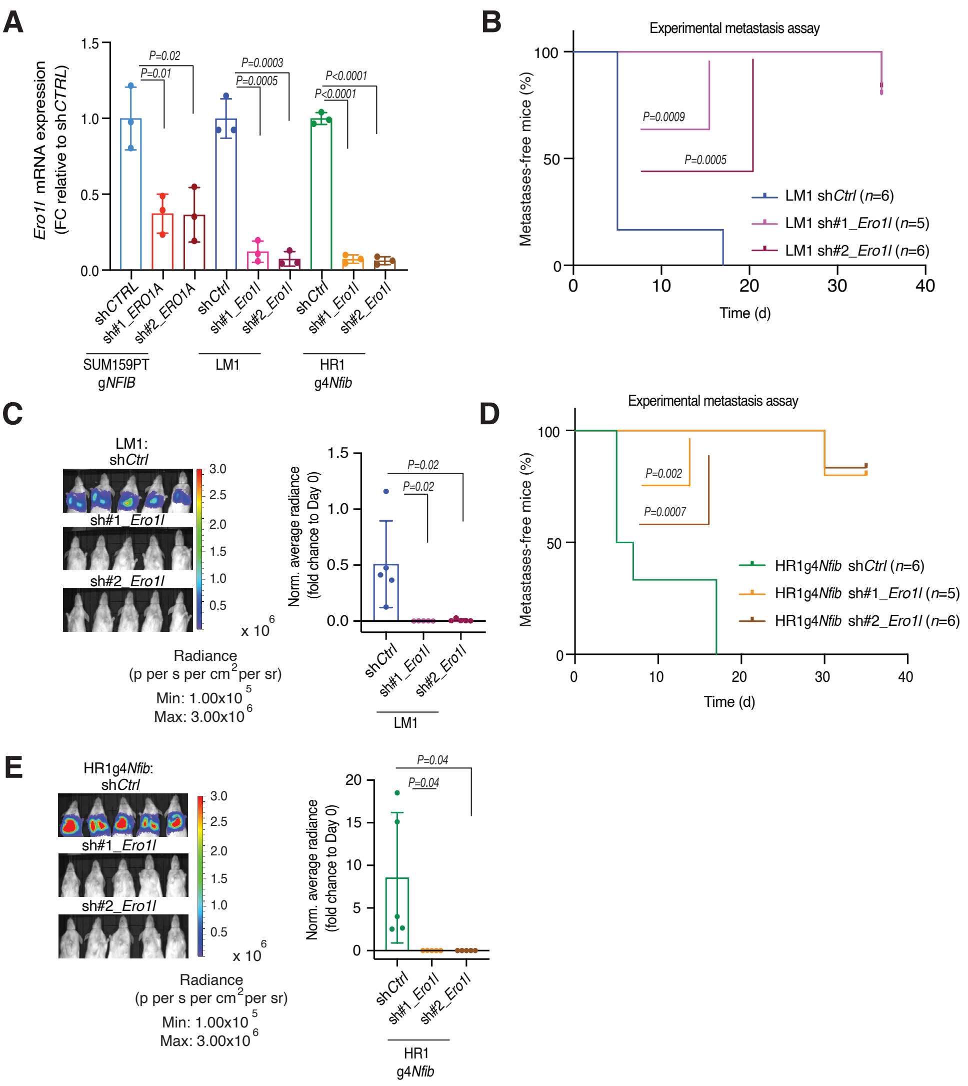

Appendix Figure S5. The NFIB-ERO1A axis promotes lung metastatic colonization.

A Bar graph representing mean *Ero1l/ERO1A* mRNA expression. Means ± s.d., *n* = 3 biological replicates and *n* = 2 technical replicates, two-tailed Student's *t*-test, FC= fold change.

B Kaplan-Meier survival analysis of NOD/SCID mice inoculated *i.v.* with LM1 sh*Ctrl* (*n* = 6), LM1 sh1 *Ero1l* (*n* = 5), or LM1 sh2 *Ero1l* (*n* = 6) cells. Two-tailed log-rank test.

C Representative bioluminescence images (left panel) and bar plot quantification (right panel) of lung metastases from mice 7 days after *i.v.* injection as indicated. *n* = 5, means ± s.d., two-tailed Student's *t*-test.

D Kaplan-Meier survival analysis of NOD/SCID mice inoculated *i.v.* with HR1 g4*Nfib* sh*Ctrl* (*n* = 6), HR1 g4*Nfib* sh1 *Ero1l* (*n* = 5), or HR1 g4*Nfib* sh2 *Ero1l* (*n* = 6) cells. Two-tailed log-rank test.

E Representative bioluminescence images (left panel) and bar plot quantification (right panel) of lung metastases from mice 17 days after *i.v.* injection as indicated. *n* = 5, means ± s.d., two-tailed Student's *t*-test.

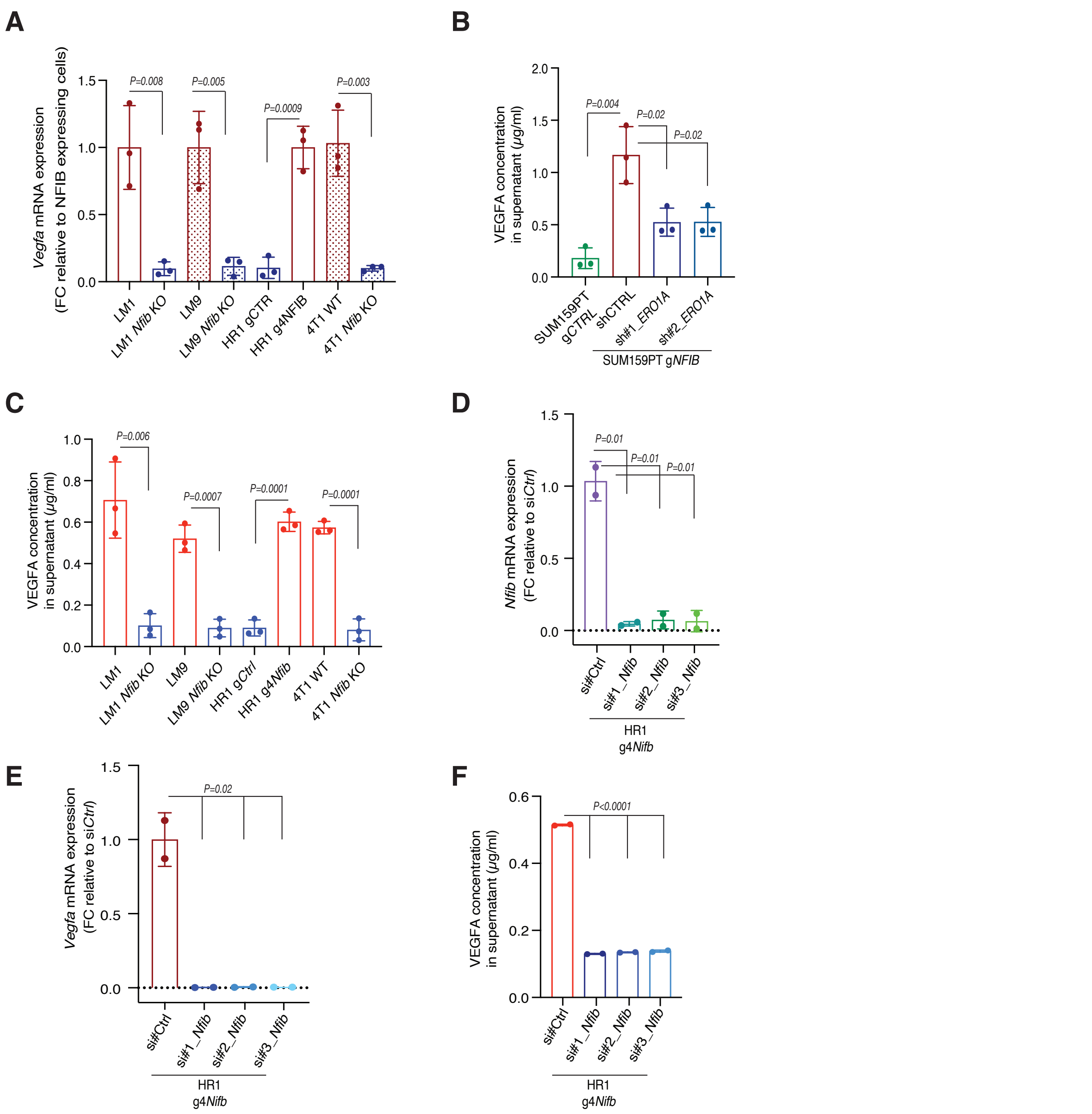

Appendix Figure S6. NFIB-ERO1A enhances VEGFA levels.

A Bar graph representing mean *Vegfa* mRNA expression. n = 3 biological replicates and n = 2 technical replicates, means ± s.d., two-tailed Student's t-test, FC= fold change.

B Bar graph representing VEGFA abundance in SUM159PT g*CTRL* and SUM159PT g*NFIB* cells upon downregulation of *ERO1A* with two independent shRNAs (sh1 and sh2) measured by ELISA. n = 3 biological replicates and n = 2 technical replicates, means ± s.d., two-tailed Student's t-test.

C Bar graph representing VEGFA abundance measured by ELISA. n = 3 biological replicates and n = 2 technical replicates, means ± s.d., two-tailed Student's t-test.

D Bar graph representing mean *Nfib* mRNA expression in HR1 g4*NFIB* upon downregulation of *Nfib* with three independent siRNAs. n = 2 biological replicates and n = 2 technical replicates, means ± s.d., two-tailed Student's t-test.

E	Bar graph representing mean *Vegfa* mRNA expression in HR1 g4*Nfib* upon downregulation of *Nfib* with three independent siRNAs. n = 2 biological replicates and n = 2 technical replicates, means ± s.d., two-tailed Student's t-test.

F	Bar graph representing VEGFA abundance in HR1 g4*Nfib* upon downregulation of *Nfib* with three independent siRNAs measured by ELISA. n = 3 biological replicates and n = 2 technical replicates, means ± s.d., two-tailed Student's t-test.

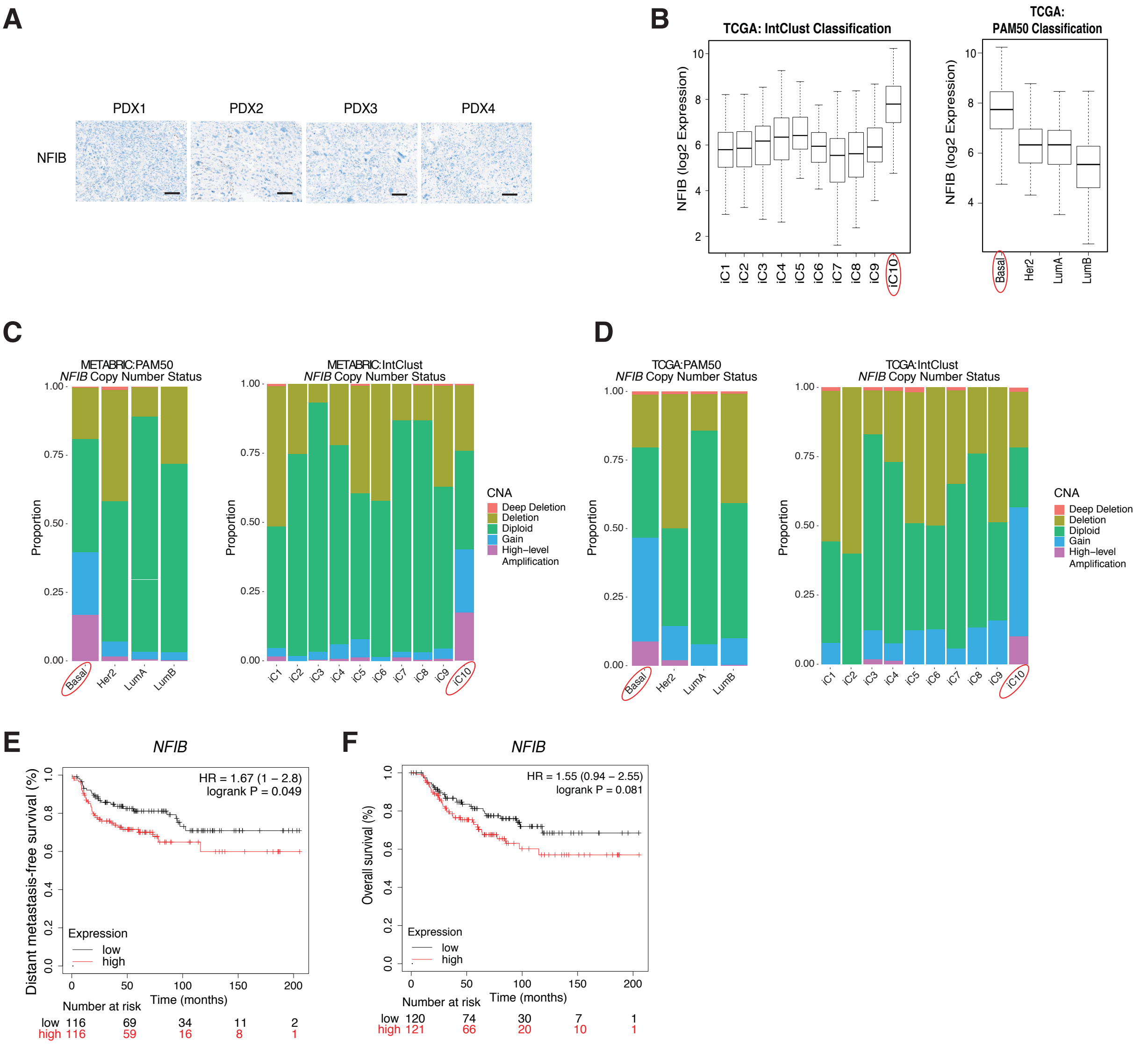

Appendix Figure S7. Elevated *NFIB* copy number and mRNA expression correlate with poor prognosis in TNBC patients.

A Representative images of NFIB-stained mammary tumours from TNBC PDXs that are negative for NFIB. Scale bar, 1 mm.

B Higher *NFIB* expression in iC10 and basal breast cancer subtype compared with the other subtypes using intClust (Dawson et al., 2013) and PAM50 classifications in the TCGA cohort.

C Higher *NFIB* gene copy number in iC10 and basal breast cancer subtype compared with the other subtypes using intClust (Dawson et al., 2013) and PAM50 classifications in the METABRIC cohort.

D Higher *NFIB* gene copy number in iC10 and basal breast cancer subtype compared with the other subtypes using intClust (Dawson et al., 2013) and PAM50 classifications in the TCGA cohort.

E, F Distant metastasis-free survival (E) and overall survival (F) plots generated using the Kaplan-Meier Plotter (Györffy et al., 2010) based on the signal intensity of the *NFIB* probe (213029_at).

In all graphs, the cut-off was automatically set to split patients into two groups (median), high and low. All the plots were generated using the signal intensity of the different probes in Affymetrix microarray gene expression data from TNBC patients of The Cancer Genome Atlas. Number of patients (*n*) and *P* values (two tailed log-rank test) are presented in the panels.
