# Supplementary material for: The NFIB‐ERO1A axis promotes breast cancer metastatic colonization of disseminated tumour cells
Source: EMBO Mol Med. 2021 Mar 10;13(4):e13162. doi: 10.15252/emmm.202013162 (PMC8033524; doi:10.15252/emmm.202013162)
Supplement: Supplementary file 2 — Expanded View Figures PDF [file EMMM-13-e13162-s002.pdf]

Expanded View Figures

Figure EV1. *In vivo* unbiased transposon screen in the non-metastatic *PIK3CA*^{H1047R} mammary tumour-derived cells allows the isolation of aggressive lung metastatic lines.

A Kaplan–Meier plot depicting tumour incidence in NOD/SCID animals inoculated in the mammary fat pad with 10^6 cells of HR1. Ctrl (n = 14) or HR1. PB cells (n = 19), two-tailed log-rank test.
B Representative bioluminescence acquisition of lungs from mice injected with HR1. PB or HR1. Ctrl cells as in A.
C Graph representing the kinetics of HR1. Ctrl (n = 5), HR1. PB (n = 4), HR1. LM1 (n = 6), HR1. LM3 (n = 5), HR1. LM4 (n = 6), HR1. LM5 (n = 5), HR1. LM6 (n = 4), and HR1. LM9 (n = 8) tumour growth upon orthotopic injection of 250×10^3 cells in NOD/SCID mice, means ± s.d.
D Bar graph showing tumour volumes in mice injected with HR1. Ctrl (n = 5), HR1. PB (n = 4), HR1. LM1 (n = 6), HR1. LM3 (n = 5), HR1. LM4 (n = 6), HR1. LM5 (n = 5), HR1. LM6 (n = 4), or HR1. LM9 (n = 8), means ± s.d.
E Kaplan–Meier plot showing metastasis onset after tumour removal in mice injected with HR1. Ctrl (n = 6), HR1. PB (n = 8), HR1. LM1 (n = 5), HR1. LM3 (n = 3), HR1. LM4 (n = 5), HR1. LM5 (n = 3), HR1. LM6 (n = 7), or HR1. LM9 (n = 7).
F Bar graph showing metastatic incidence after tumour removal in mice injected with HR1. Ctrl (n = 6), HR1. PB (n = 8), HR1. LM1 (n = 5), HR1. LM3 (n = 3), HR1. LM4 (n = 5), HR1. LM5 (n = 3), HR1. LM6 (n = 7), or HR1. LM9 (n = 7).
G Representative bioluminescence images (left panel) and bar plot quantification (right panel) of lung metastases after primary tumour removal in mice injected with metastatic or non-metastatic LM cells. n = 3 mice, means ± s.d., two-tailed Student's t-test.

Source data are available online for this figure.

© 2021 The Authors

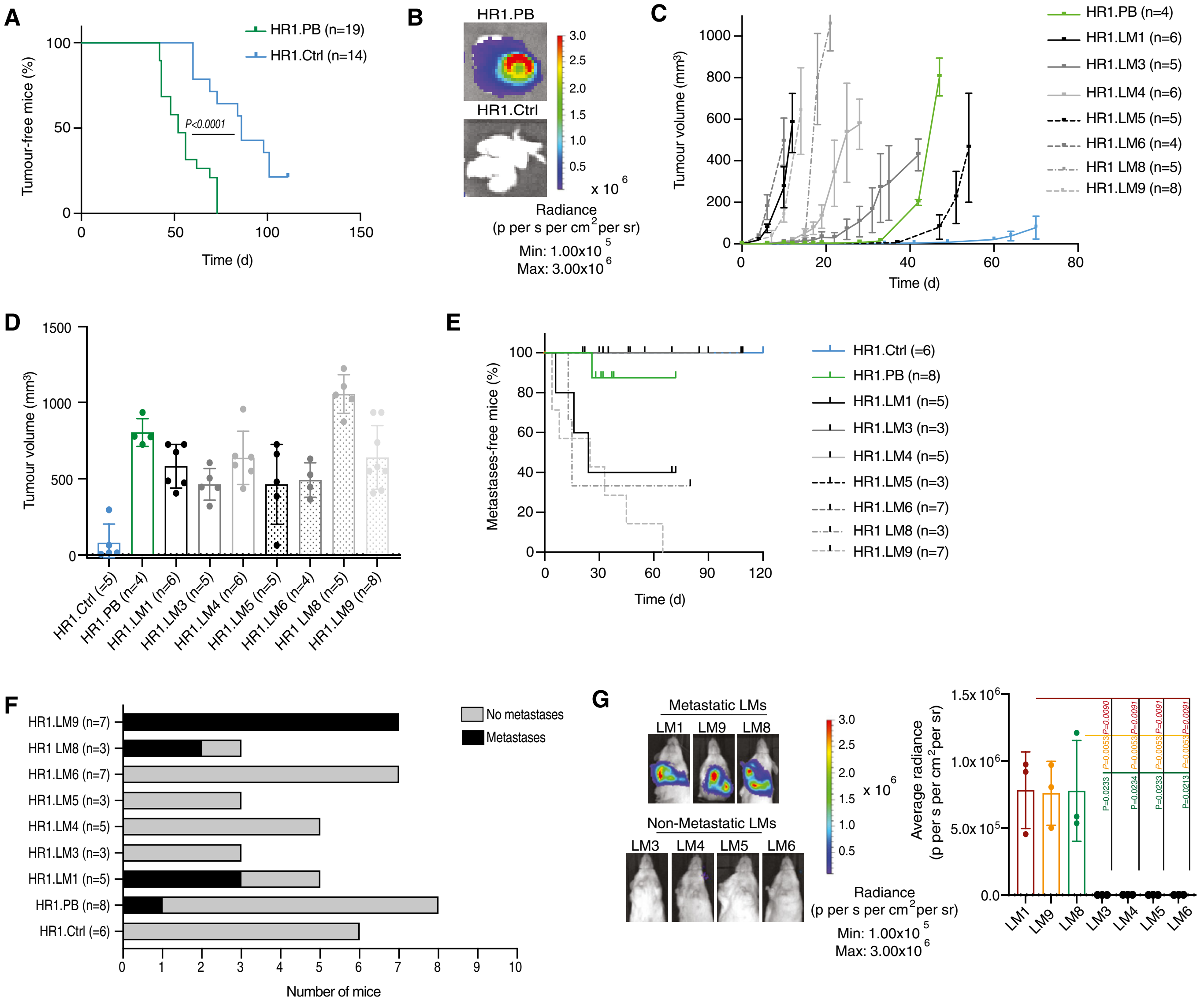
A
HR1.PB (n=19)
HR1.Ctrl (n=14)
P<0.0001
Tumour-free mice (%)
Time (d)
B
HR1.PB
HR1.Ctrl
x 10 6
Radiance
(p per s per cm2 per sr)
Min: 1.00x10 5
Max: 3.00x10 6
C
Tumour volume (mm³)
Time (d)
HR1.Ctrl (=5)
HR1.PB (n=4)
HR1.LM1 (n=6)
HR1.LM3 (n=5)
HR1.LM4 (n=6)
HR1.LM5 (n=5)
HR1.LM6 (n=4)
HR1 LM8 (n=5)
HR1.LM9 (n=8)
D
Tumour volume (mm³)
HR1.Ctrl (=5)
HR1.PB (n=4)
HR1.LM1 (n=6)
HR1.LM3 (n=5)
HR1.LM4 (n=6)
HR1.LM5 (n=5)
HR1.LM6 (n=4)
HR1 LM8 (n=5)
HR1.LM9 (n=8)
E
Metastases-free mice (%)
Time (d)
HR1.Ctrl (=6)
HR1.PB (n=8)
HR1.LM1 (n=5)
HR1.LM3 (n=3)
HR1.LM4 (n=5)
HR1.LM5 (n=3)
HR1.LM6 (n=7)
HR1 LM8 (n=3)
HR1.LM9 (n=7)
F
No metastases
Metastases
HR1.LM9 (n=7)
HR1 LM8 (n=3)
HR1.LM6 (n=7)
HR1.LM5 (n=3)
HR1.LM4 (n=5)
HR1.LM3 (n=3)
HR1.LM1 (n=5)
HR1.PB (n=8)
HR1.Ctrl (=6)
Number of mice
G
Metastatic LMs
LM1 LM9 LM8
Non-Metastatic LMs
LM3 LM4 LM5 LM6
x 10 6
Radiance
(p per s per cm2 per sr)
Min: 1.00x10 5
Max: 3.00x10 6
Average radiance
(p per s per cm2 per sr)
LM1 LM9 LM8 LM3 LM4 LM5 LM6

Figure EV1.

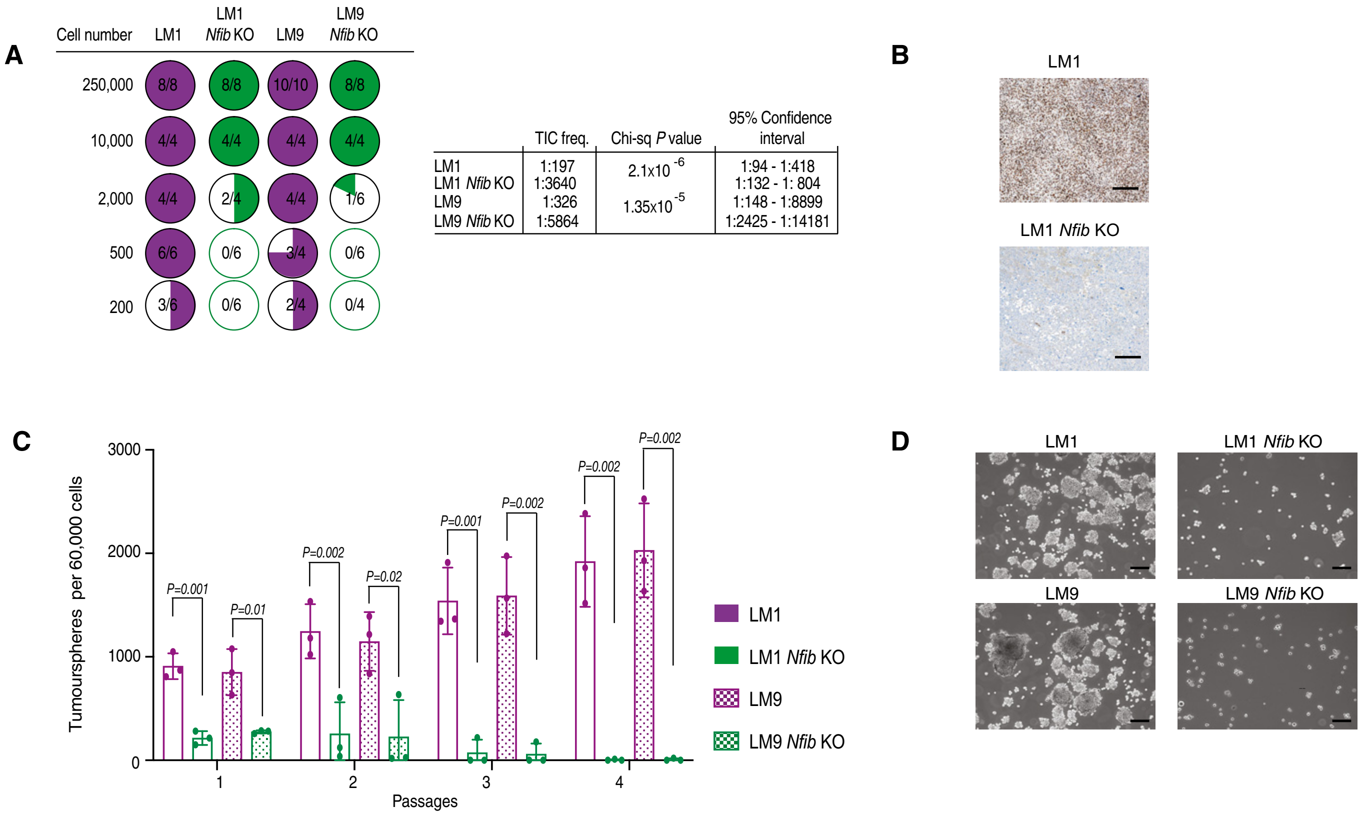

Figure EV2. ***Nfib* overexpression accelerates tumour growth.**

A Limiting dilution assay: (left panel) quantification of tumour initiation upon orthotopic injection of mice with LM1, LM9, LM1 *Nfib* KO or LM9 *Nfib* KO cells. (right panel) Tumour-initiating cell (TIC) frequencies and 95% confidence intervals were calculated using ELDA (Hu & Smyth, 2009) as described in the experimental procedures.

B Representative images of immunostaining for NFIB of tumours from mice injected with LM or LM *Nfib* KO cancer cells. Scale bar 100 μm.

C Bar graph showing tumoursphere numbers over four passages of LM1, LM9, LM1 *Nfib* KO, and LM9 *Nfib* KO, $n = 3$ biological replicates and $n = 3$ technical replicates, means ± s.d., two-tailed Student's *t*-test.

D Representative images of tumourspheres of LM1, LM9, LM1 *Nfib* KO, and LM9 *Nfib* KO cancer cell lines at the first passage. Scale bar 100 μm.

Source data are available online for this figure.

© 2021 The Authors

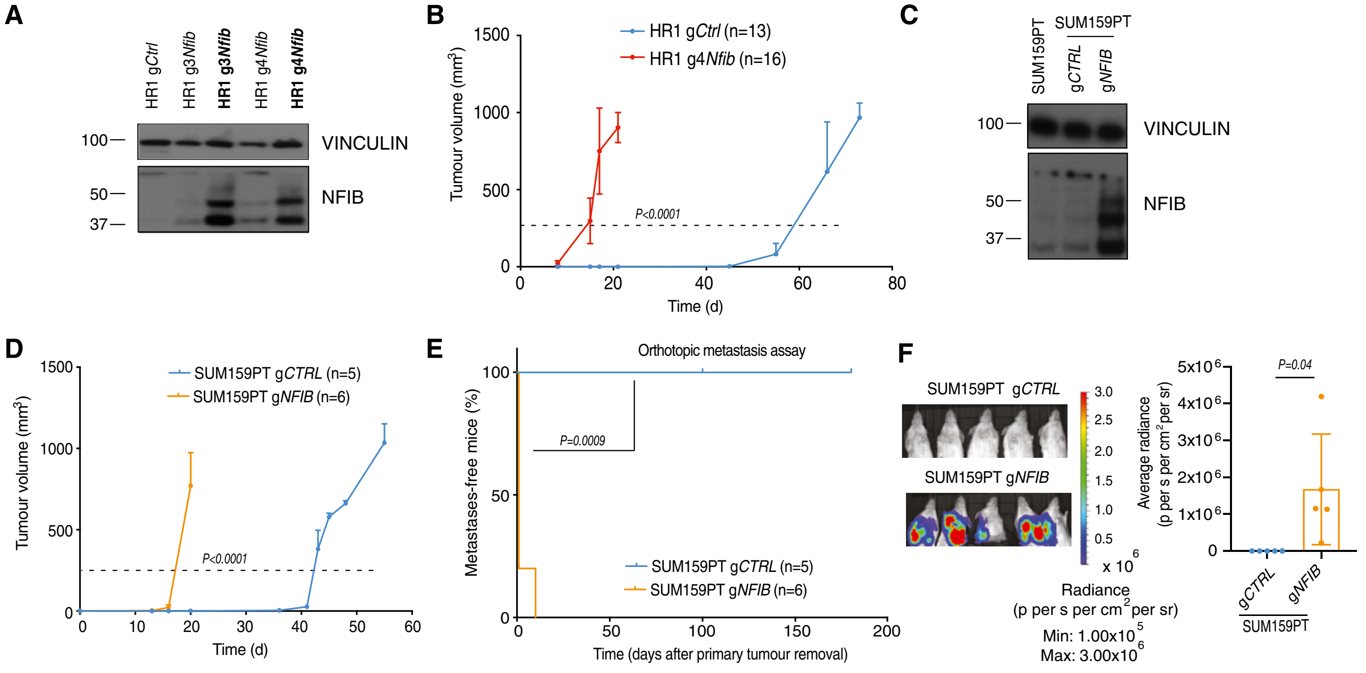

Figure EV3. Endogenous *Nfib/NFIB* overexpression in HR1 and SUM159PT cell lines decreases tumour latency and enhances metastasis.

A Immunoblot showing proteins levels of NFIB in HR1 cells overexpressing *Nfib* via the CRISPR-Cas 9 SAM system. In bold: cell lines after two months of culture *in vitro*. HR1 g4*Nfib* cells were selected for further studies. VINCULIN served as a loading control.

B Graph representing the kinetics of HR1 g*Ctrl* ($n = 13$) and HR1 g4*Nfib* ($n = 16$) tumour growth upon orthotopic injection of 250×10^3 cells in NOD/SCID mice. Curves show means of tumour volume ± s.d., two-tailed Student's *t*-test on the times to reach 250 mm^3 (dashed line).

C Immunoblots showing protein levels of NFIB in SUM159PT cells overexpressing *NFIB* via the CRISPR-Cas 9 SAM system. VINCULIN served as a loading control.

D Graph representing the kinetics of SUM159PT g*CTRL* ($n = 5$) and g*NFIB* ($n = 6$) tumour growth upon orthotopic injection of 250 x 10^3 cells into NSG mice. Curves show means of tumour volume ± s.d., two-tailed Student's *t*-test on the times to reach 250 mm^3 (dashed line).

E Kaplan–Meier plot depicting metastasis onset after tumour removal from mice injected orthotopically with SUM159PT g*CTRL* ($n = 5$) or g*NFIB* ($n = 6$), two-tailed log-rank test.

F Representative bioluminescence images (left panel) and quantification (right panel) of lung metastases at 30 (SUM159PT g*CTRL*) and 3 (SUM159PT g*NFIB*) days after primary tumour removal. $n = 5$ mice, means ± s.d., two-tailed Student's *t*-test.

Source data are available online for this figure.

Figure EV4. The NFIB-ERO1A-VEGFA axis promotes lung metastatic colonization through angiogenesis.

A Representative images of VEGFA-stained tumours. Scale bar, 1 mm.

B Bar graph showing quantification of VEGFA staining in the tumours. Means ± s.d., $n = 2$, two-tailed Student's *t*-test.

C Left panel: Representative images of VEGFA-positive lung metastases. Scale bar, 1 mm. Right panel: Bar graph showing quantification of VEGFA. Means ± s.d., $n = 4$ SUM159PT g*NFIB* sh*CTRL*, $n = 4$ sh1 *ERO1A*, and $n = 4$ sh2 *ERO1A*, two-tailed Student's *t*-test.

D Left panel: Representative images of VEGFA-positive lung metastases. Scale bar, 1 mm. Right panel: Bar graph showing quantification of VEGFA. Means ± s.d., $n = 5$ HR1g4*Nfib* and LM1 sh*CTRL*, $n = 5$ sh1 *ERO1A*, and $n = 5$ sh2 *ERO1A*, two-tailed Student's *t*-test.

E Representative image of CD31-positive endothelial structures in tumours. Scale bar, 1 mm.

F Bar graph showing quantification of CD31 staining in tumours. Means ± s.d., $n = 2$, two-tailed Student's *t*-test.

G Left panel: Representative images of CD31-positive endothelial structures in lung metastases. Scale bar, 1 mm. Right panel: Bar graphs showing quantification of metastases as percentage of metastatic area per lung and quantification of CD31 staining in the metastatic area. Means ± s.d., $n = 6$ HR1 gCtrl and $n = 6$ HR1 g4*Nfib*, two-tailed Student's *t*-test.

Source data are available online for this figure.

▶

© 2021 The Authors

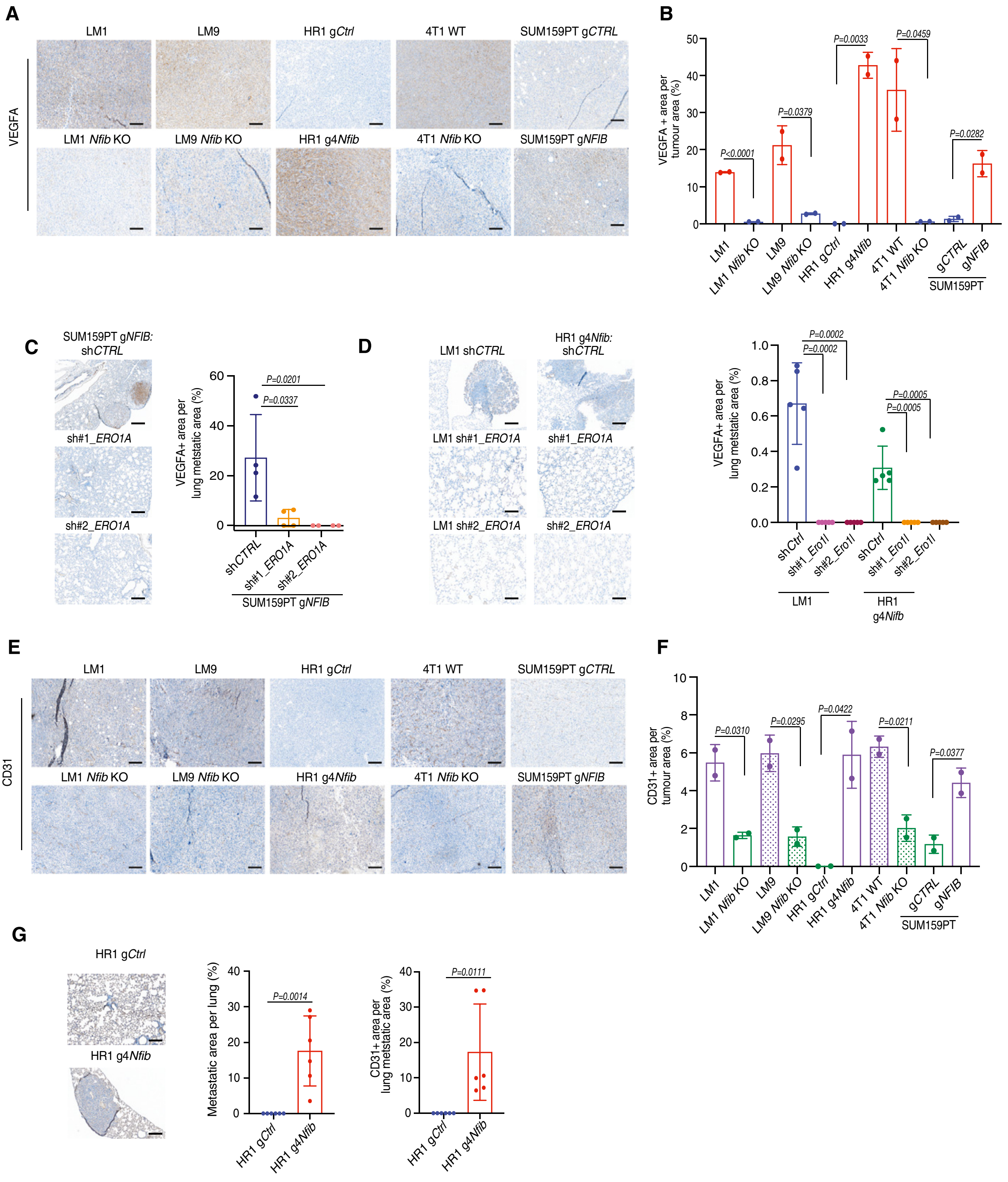

Figure EV4.

© 2021 The Authors

Figure EV5. *Ero1l* overexpression in *Nfib* KO cells restores *Vegfa* expression and metastasis.

A Bar graph representing mean *Ero1l* mRNA expression in the LM1, LM9, 4T1 WT with respective *Nfib* KO cell lines and *Ero1l* overexpression (OE) as indicated. Means ± s.d., n = 2 biological replicates and n = 2 technical replicates, two-tailed Student's t-test, FC = fold change.
B Kaplan–Meier survival analysis of NOD/SCID mice inoculated *i.v.* with LM1 *Nfib* KO (n = 6), LM1 *Nfib* KO Ero1l OE (n = 6), LM9 *Nfib* KO (n = 6) or LM9 *Nfib* KO Ero1l OE (n = 6) cells. Two-tailed log-rank test.
C Representative bioluminescence images (left panel) and quantification (right panel) of lung metastases 5 days after *i.v.* injection of LM1 *Nfib* KO (n = 4) or LM1 *Nfib* KO Ero1l OE (n = 4) cells, means ± s.d., two-tailed Student's t-test.
D Bar graph representing mean *Vegfa* mRNA expression in the LM1, LM9, 4T1 WT with respective *Nfib* KO cell lines and *Ero1l* overexpression. Means ± s.d., n = 2 biological replicates and n = 2 technical replicates, two-tailed Student's t-test, FC = fold change.
E Left panel: Representative images of VEGFA-positive lung metastases. Scale bar, 1 mm. Right panel: Bar graph showing quantification of VEGFA staining in the metastatic area. Means ± s.d., n = 5 LM1/LM9 *Nfib* KO and LM1/LM9 *Nfib* KO Ero1l OE, two-tailed Student's t-test.

Source data are available online for this figure.

© 2021 The Authors

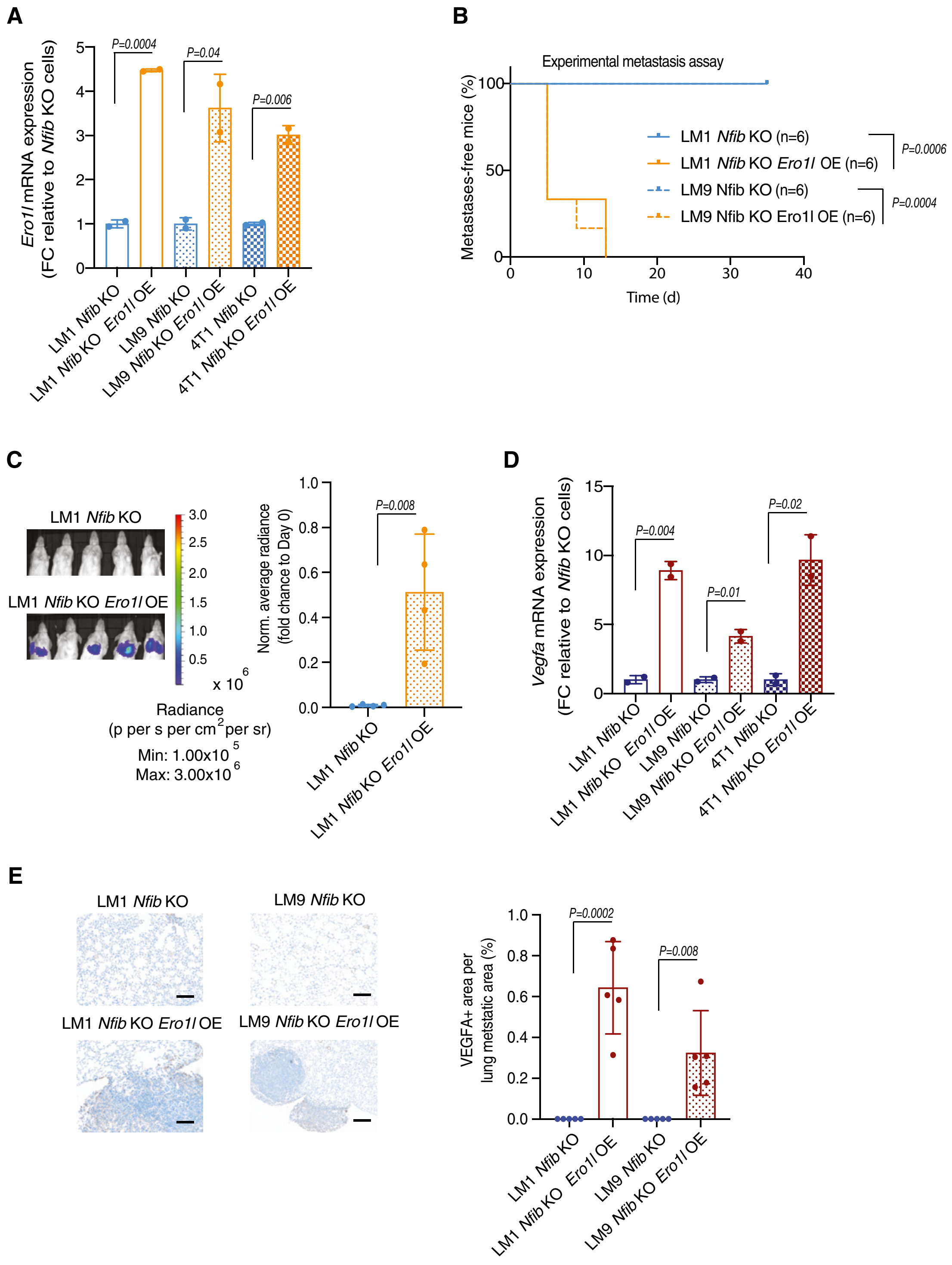

Figure EV5.

 © 2021 The Authors
