# Supplementary figures and images for: The NFIB‐ERO1A axis promotes breast cancer metastatic colonization of disseminated tumour cells
Source: EMBO Mol Med. 2021 Mar 10;13(4):e13162. doi: 10.15252/emmm.202013162 (PMC8033524; doi:10.15252/emmm.202013162)

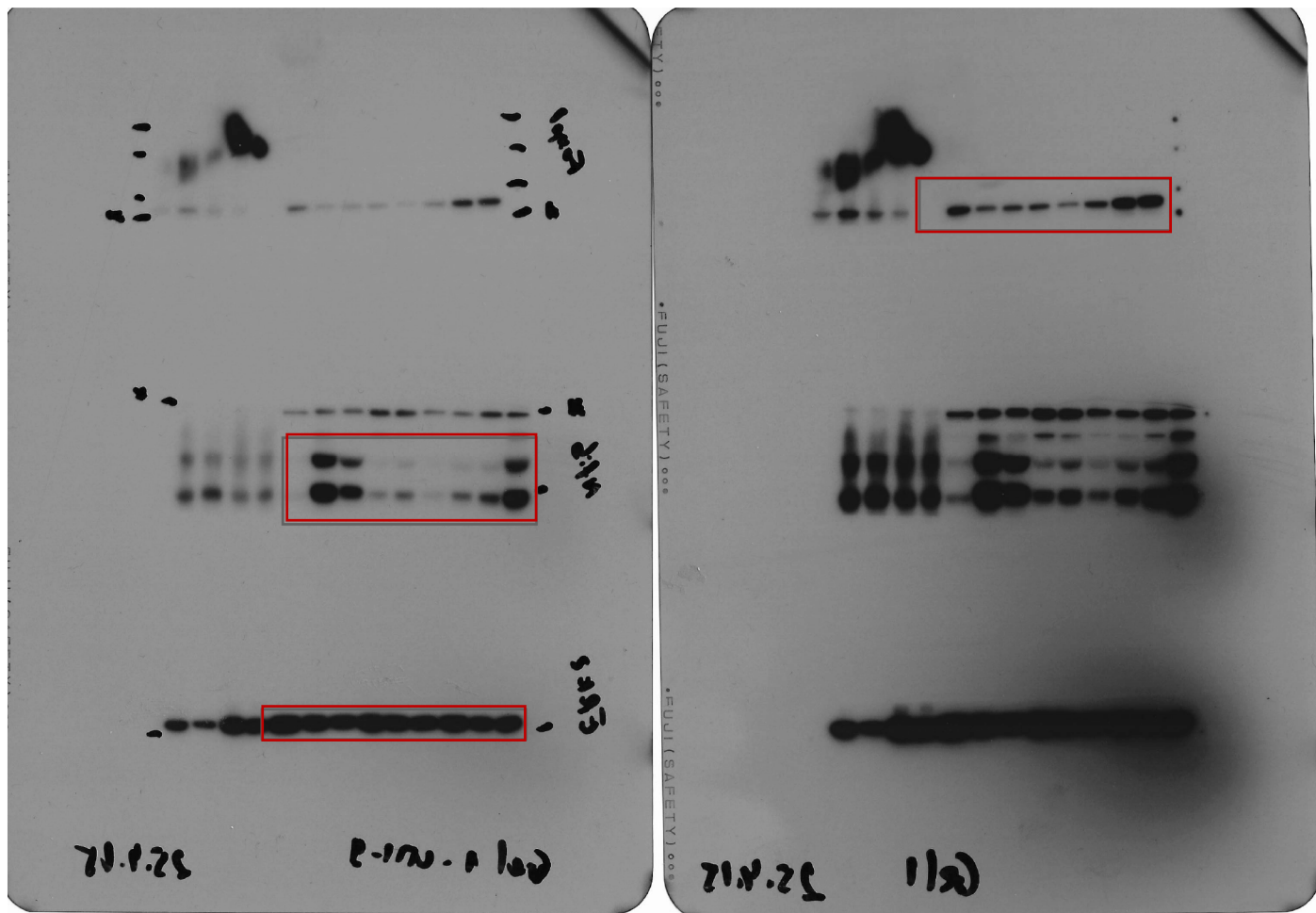

Supplement: Supplementary file 9 — Source Data for Figure 1 [file EMMM-13-e13162-s012.zip › SourceDataFor_Figure1.pdf]

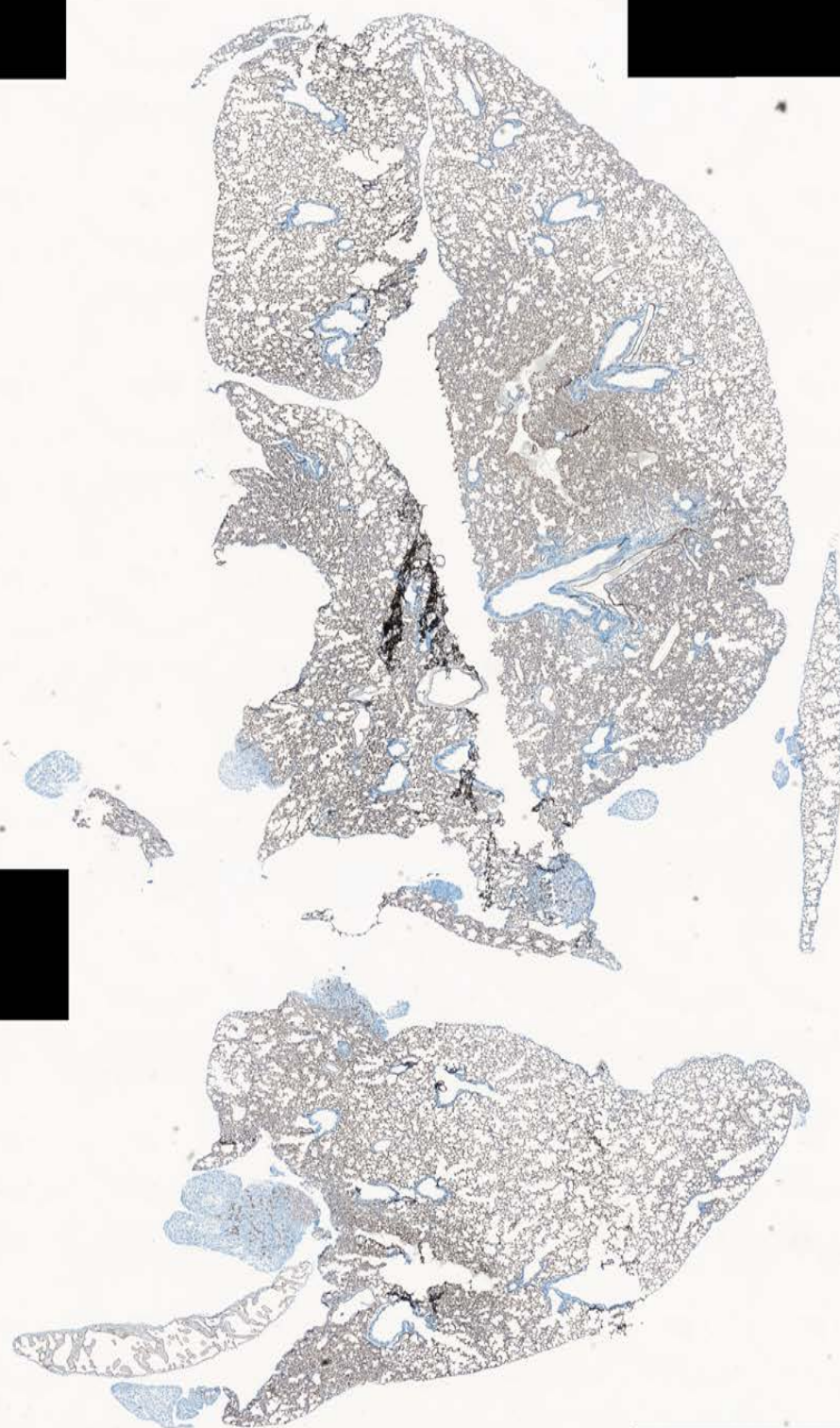

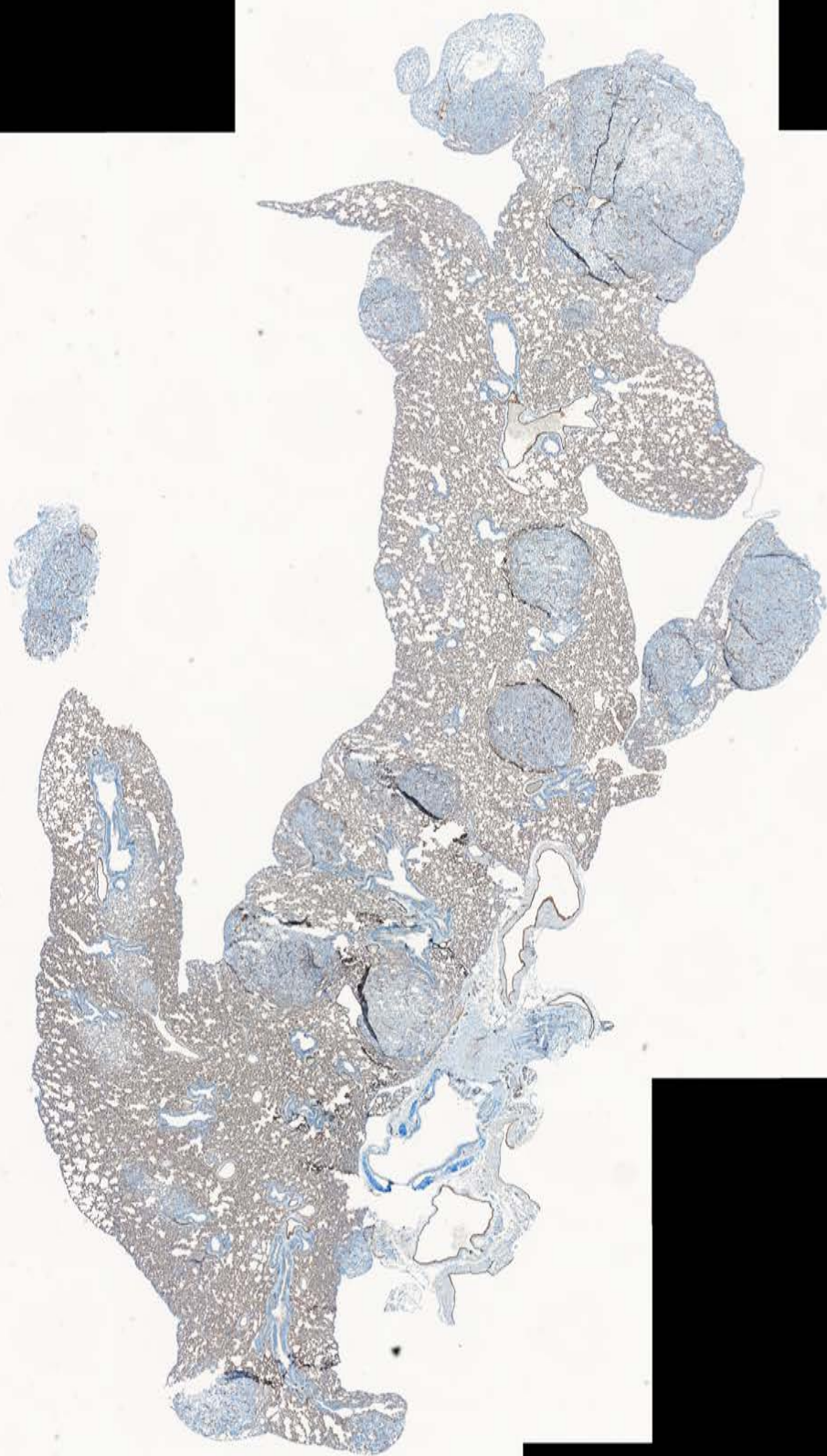

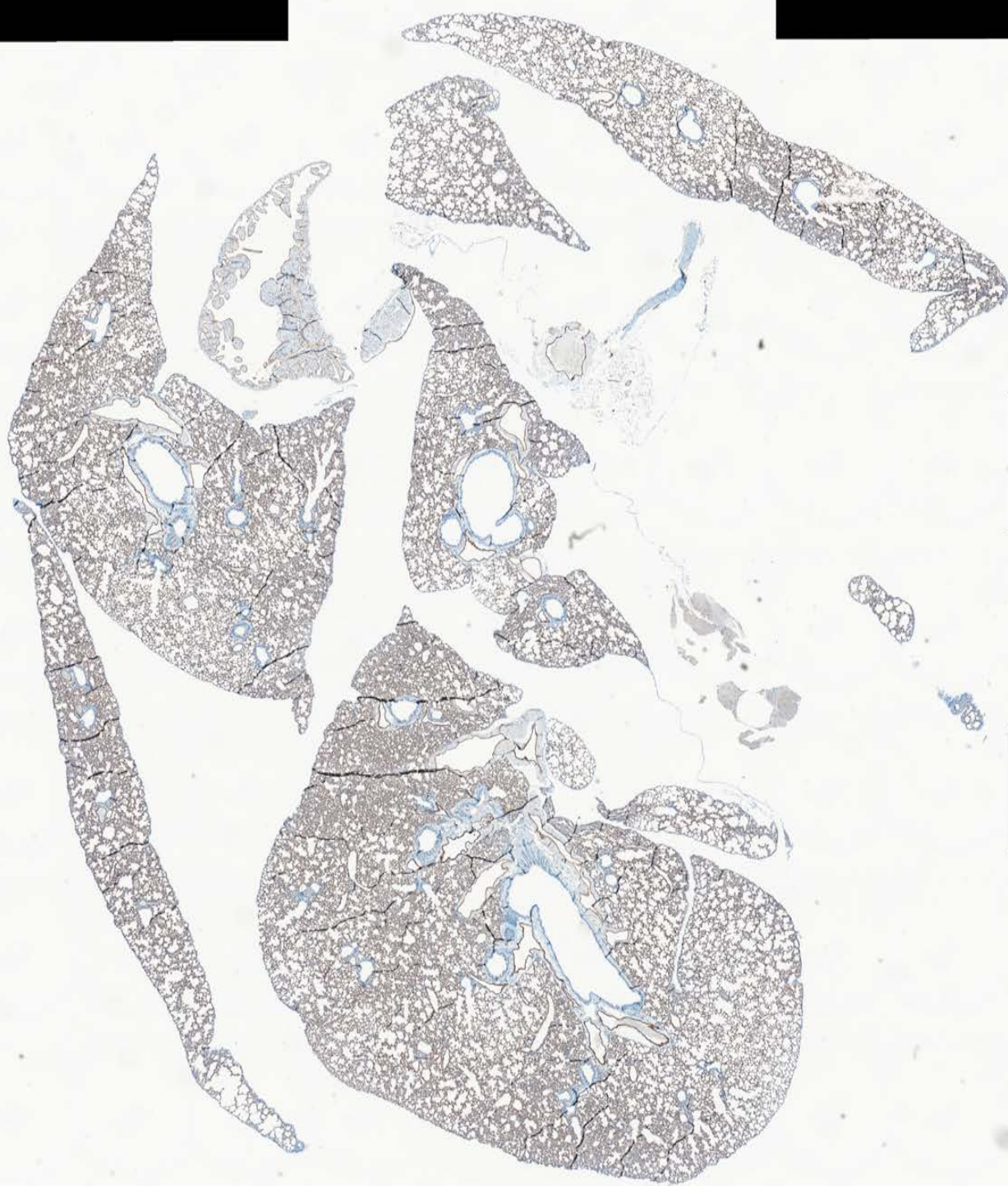

Supplement: Supplementary file 13 — Source Data for Figure 5 [file EMMM-13-e13162-s009.zip › SourceDataFor_Figure5D.pdf]

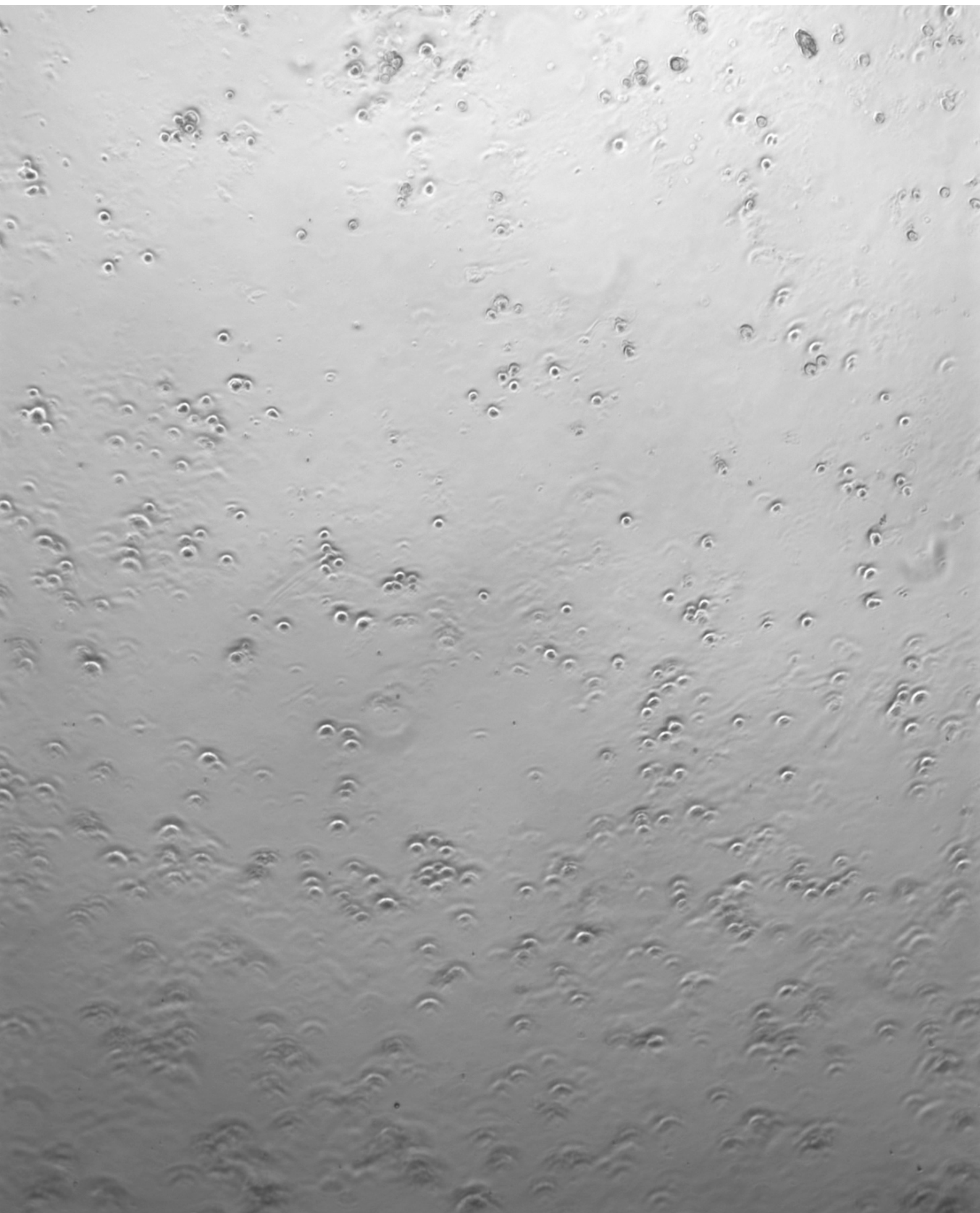

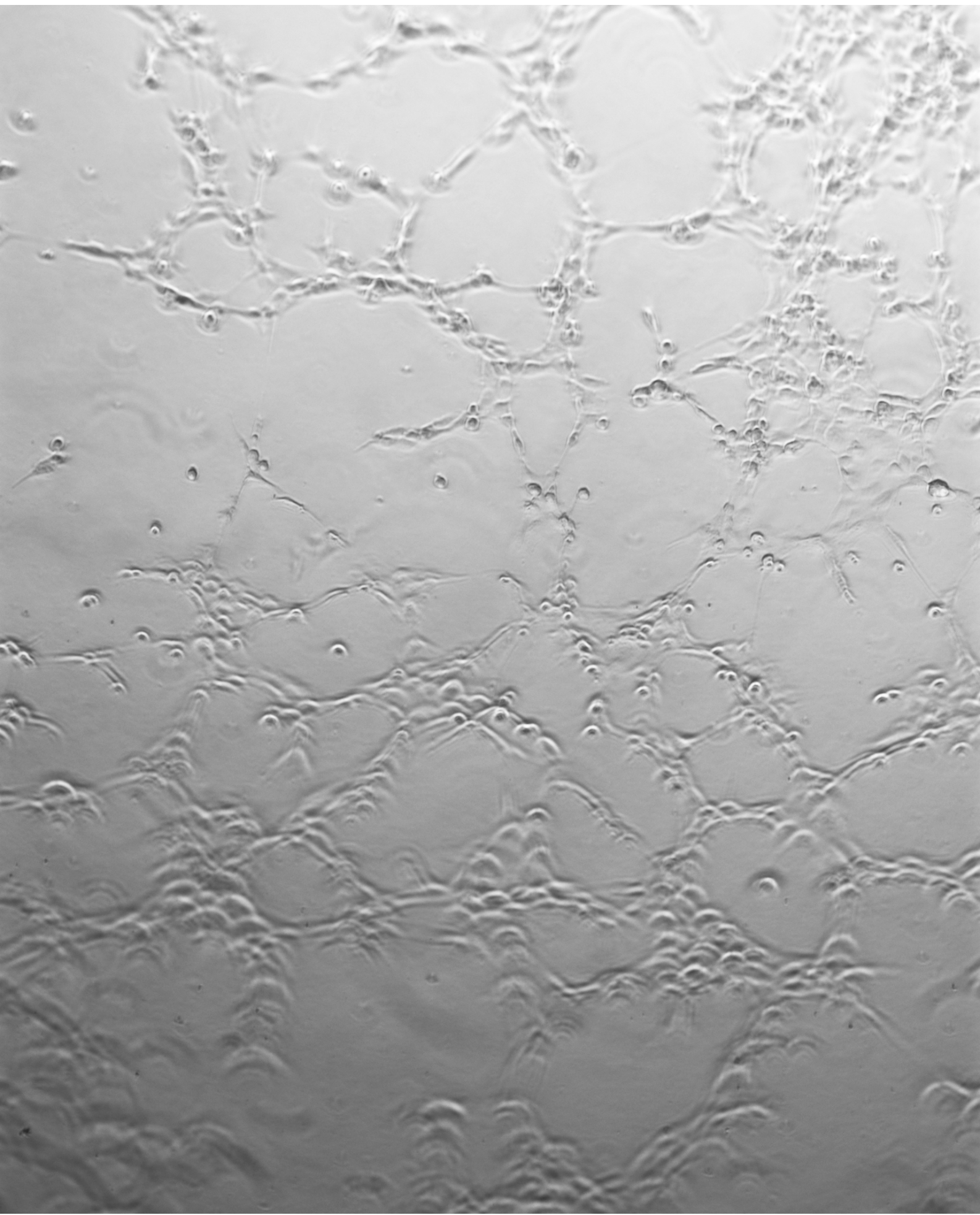

Supplement: Supplementary file 13 — Source Data for Figure 5 [file EMMM-13-e13162-s009.zip › SourceDataFor_Figure5E.pdf]

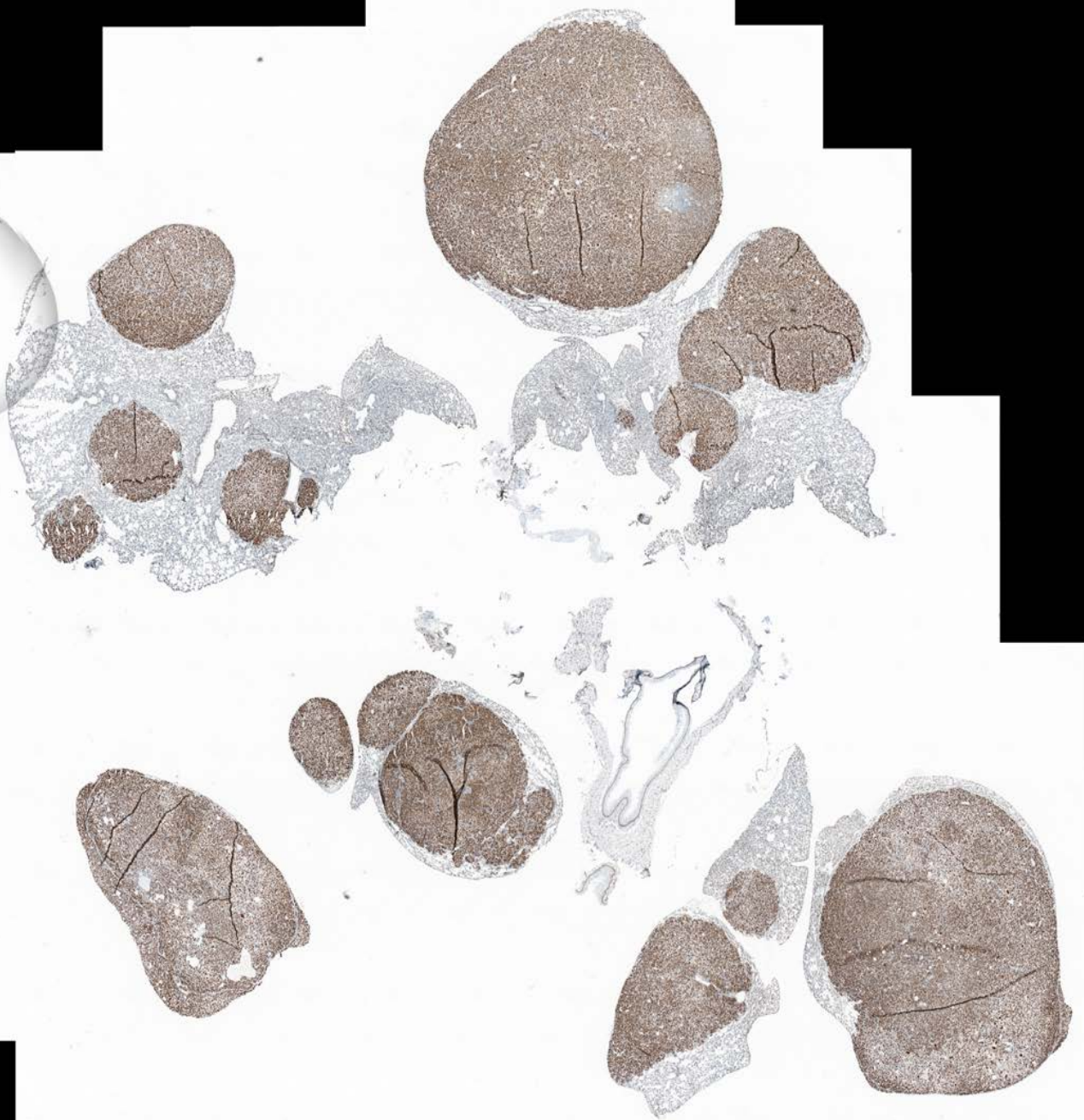

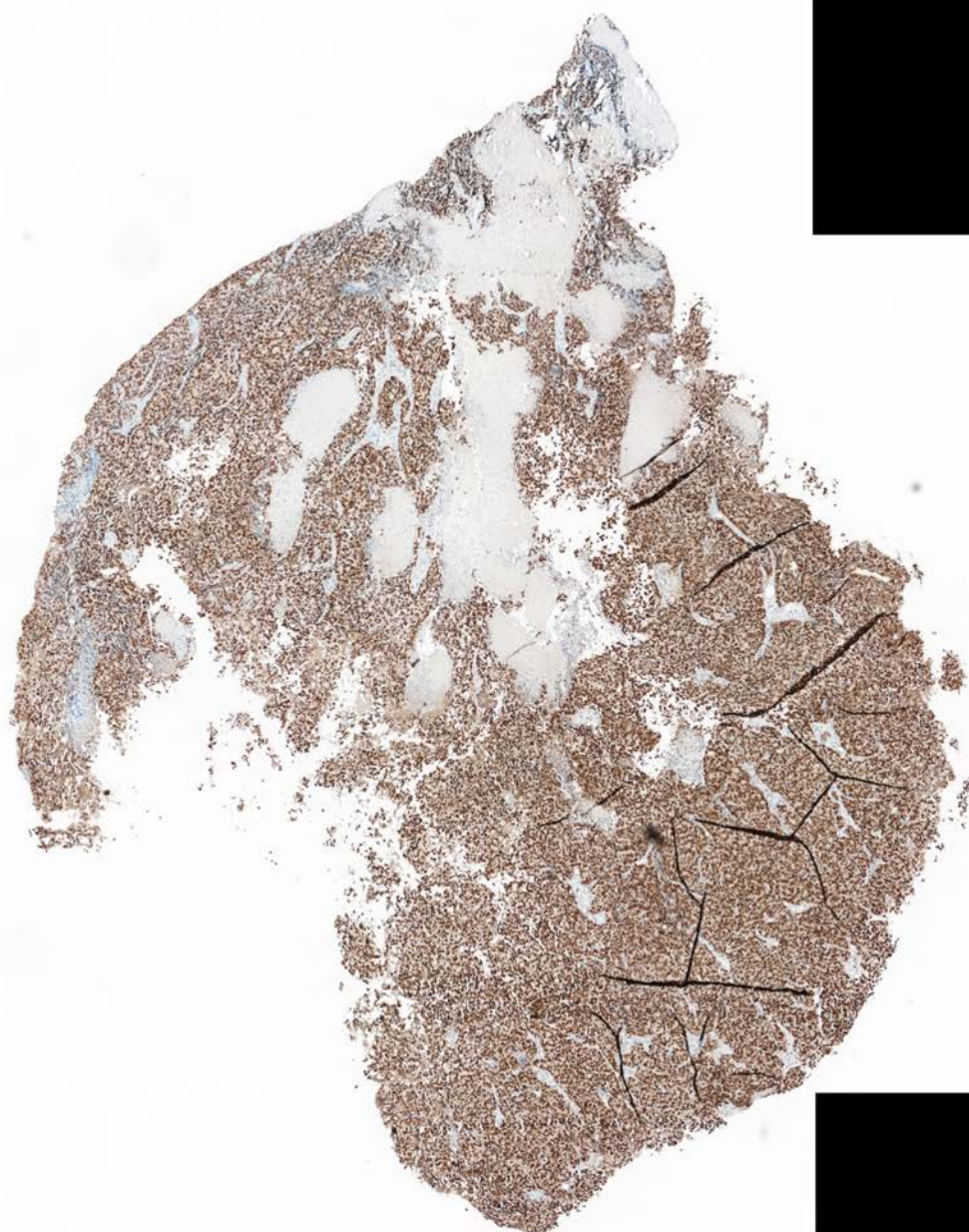

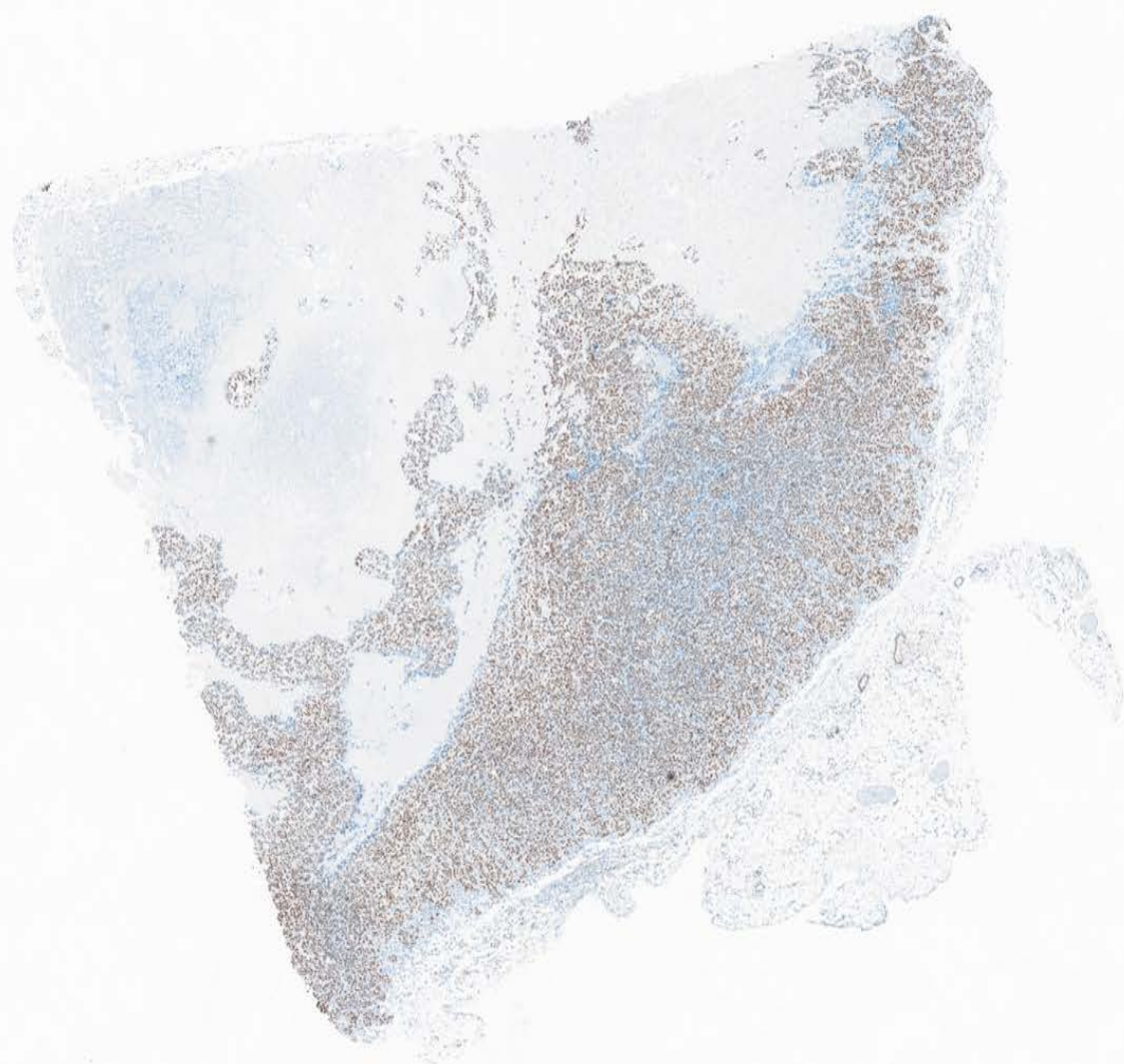

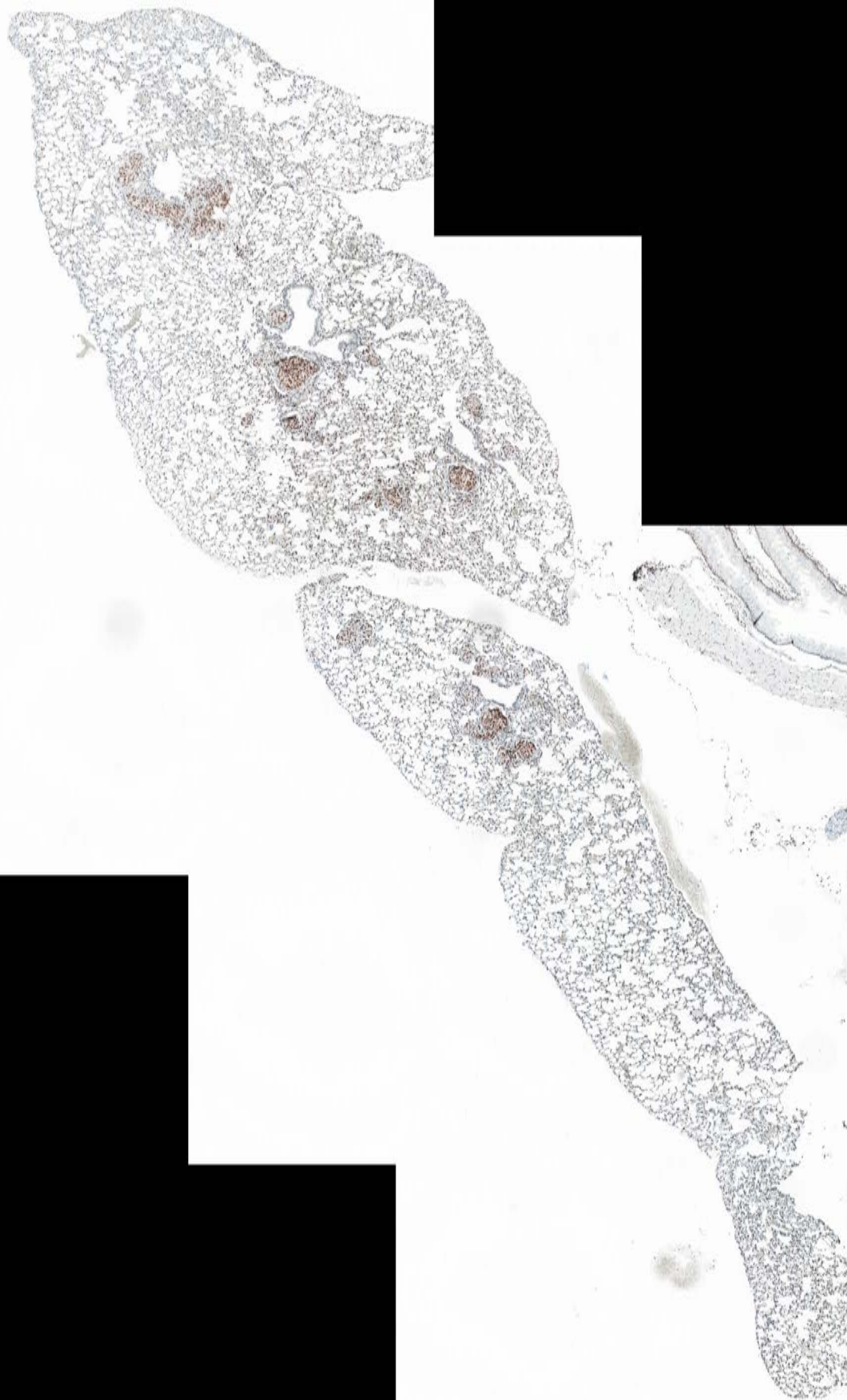

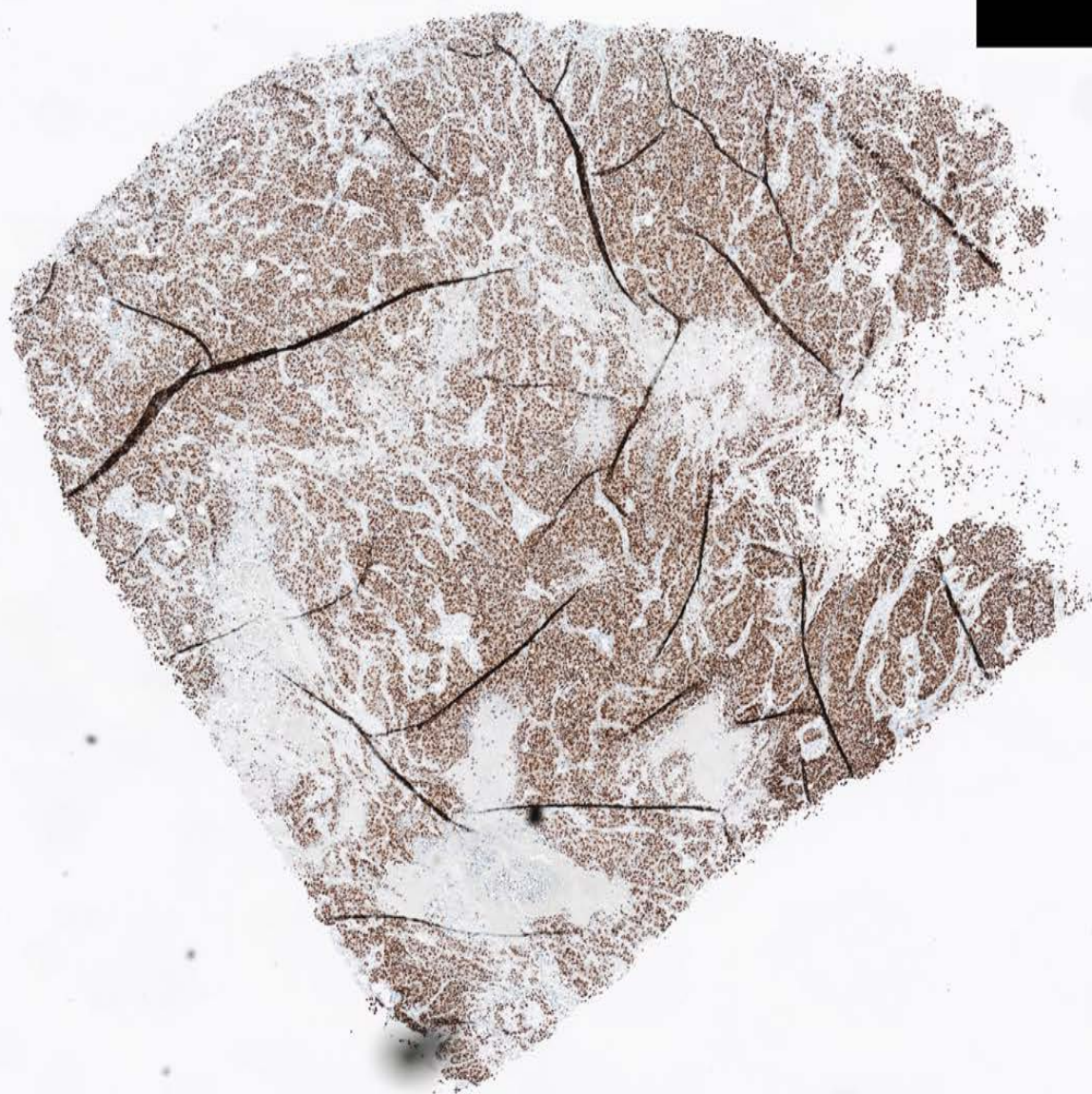

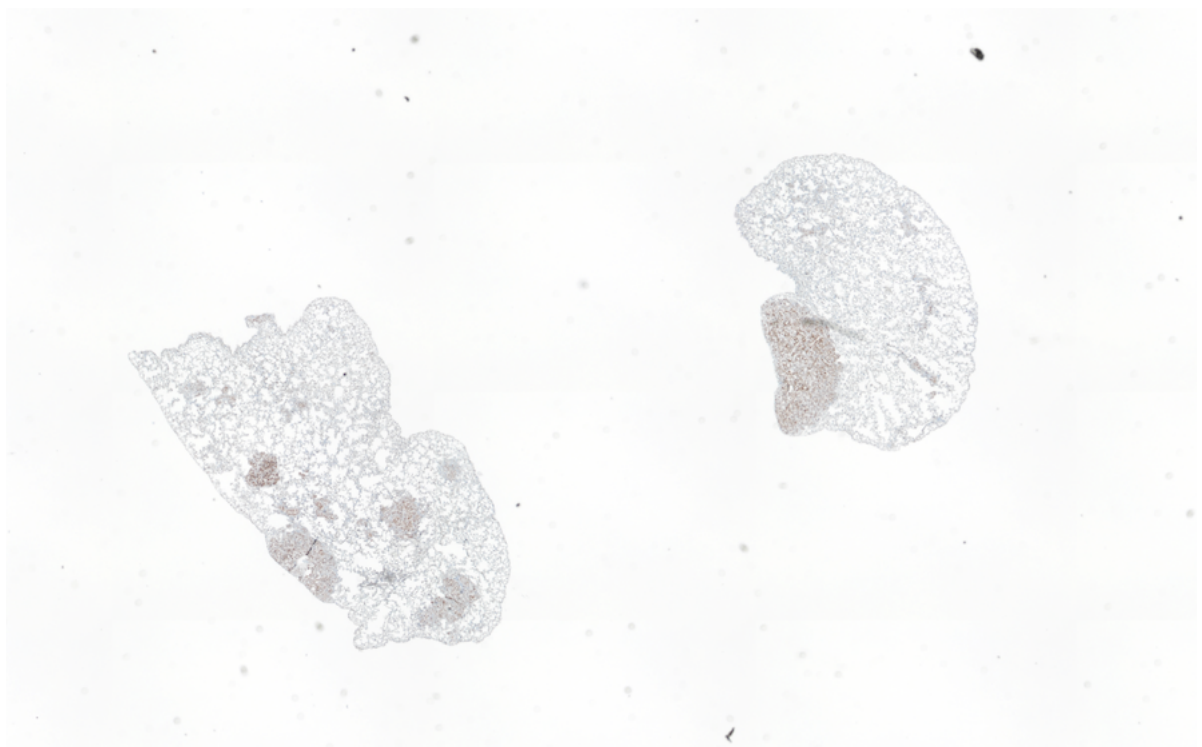

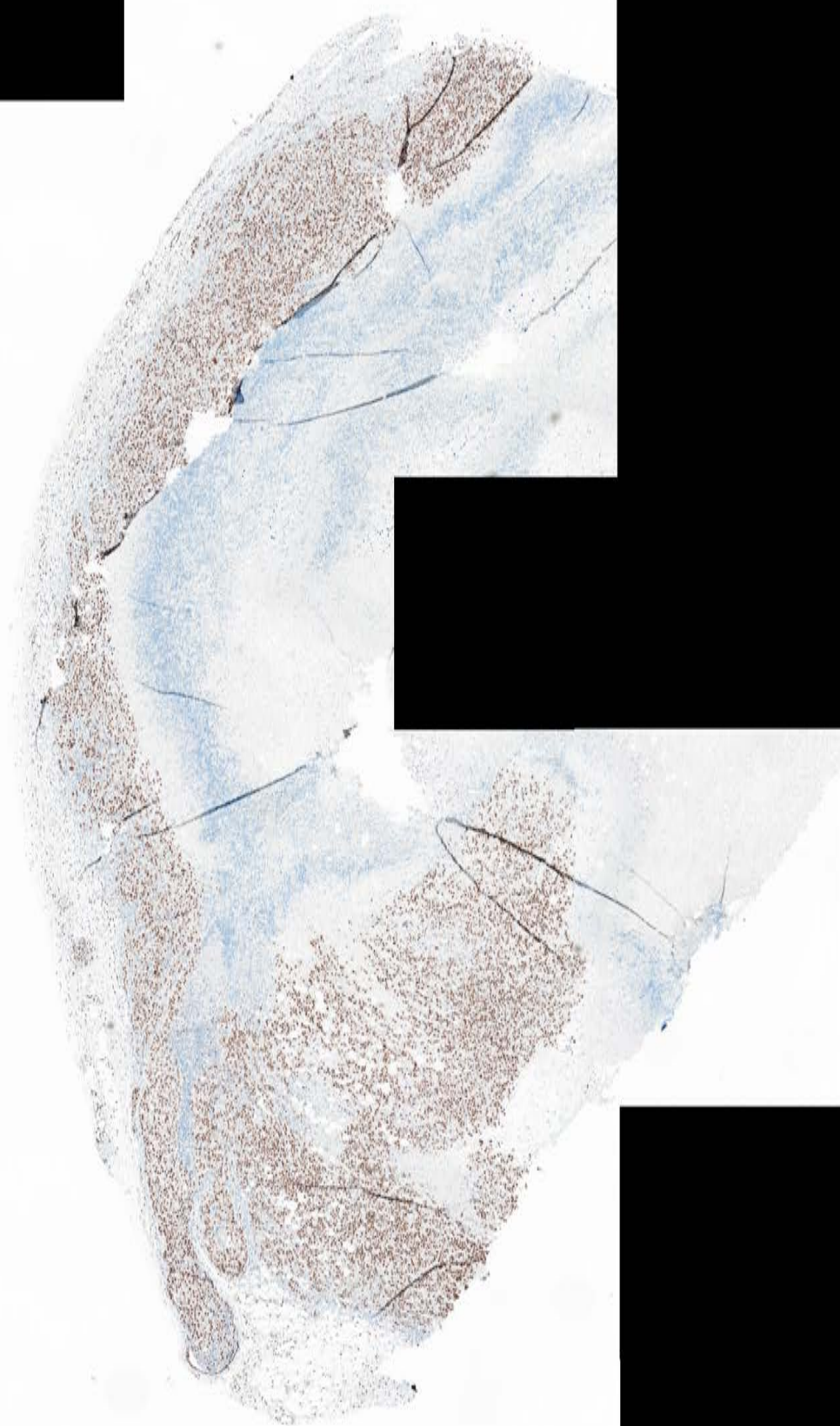

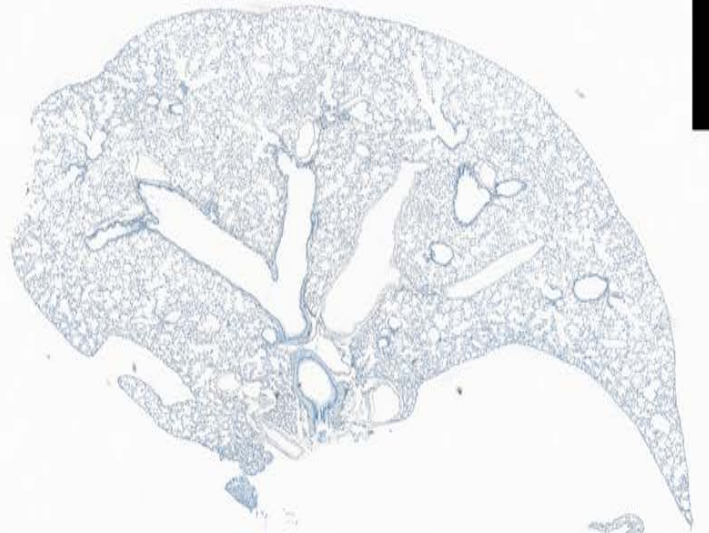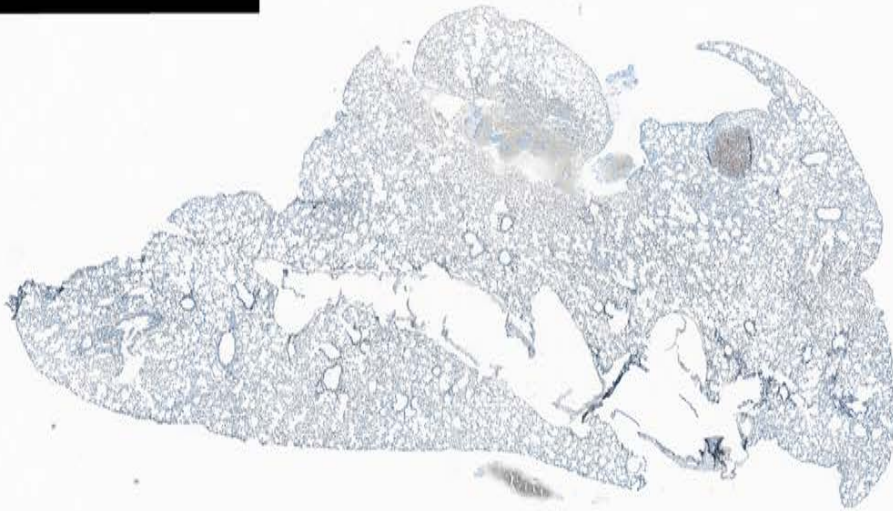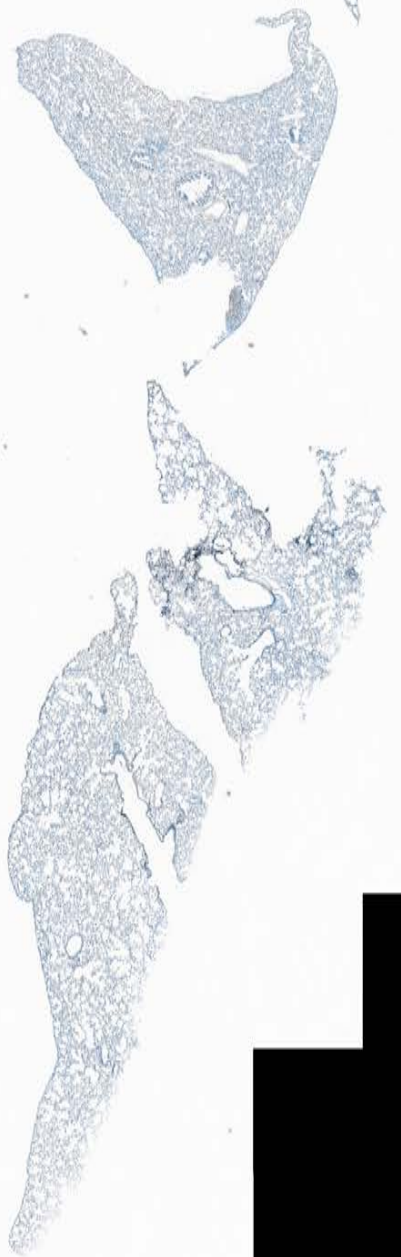

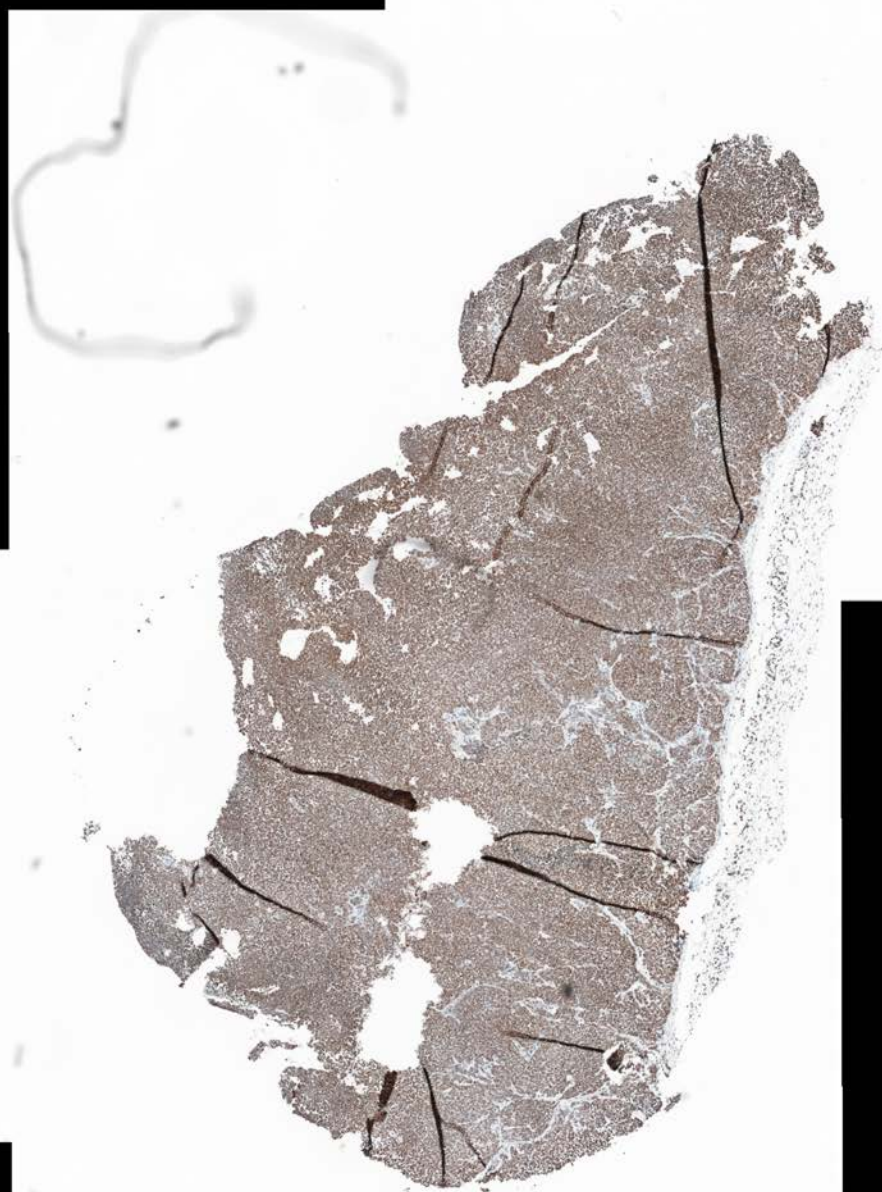

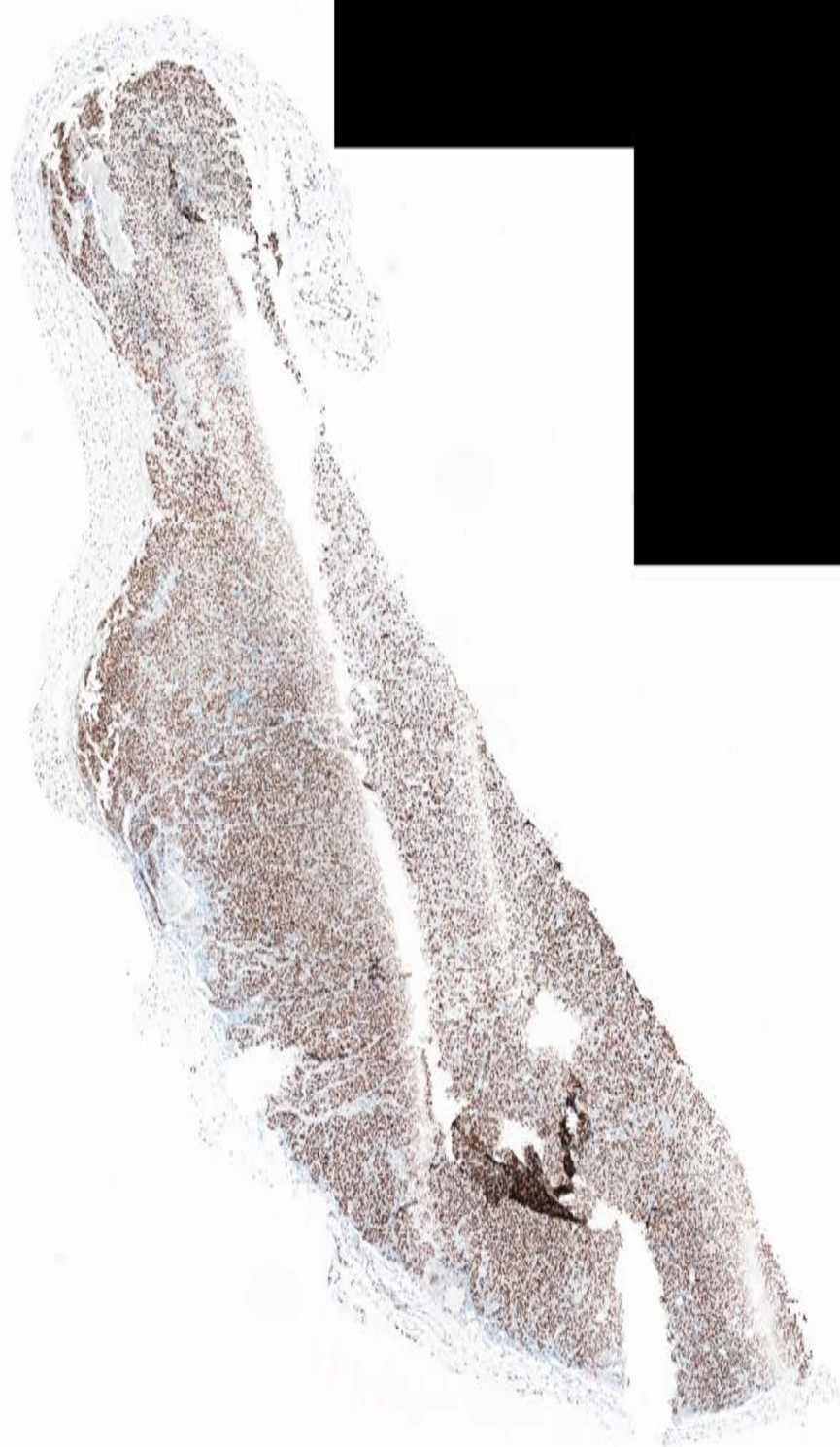

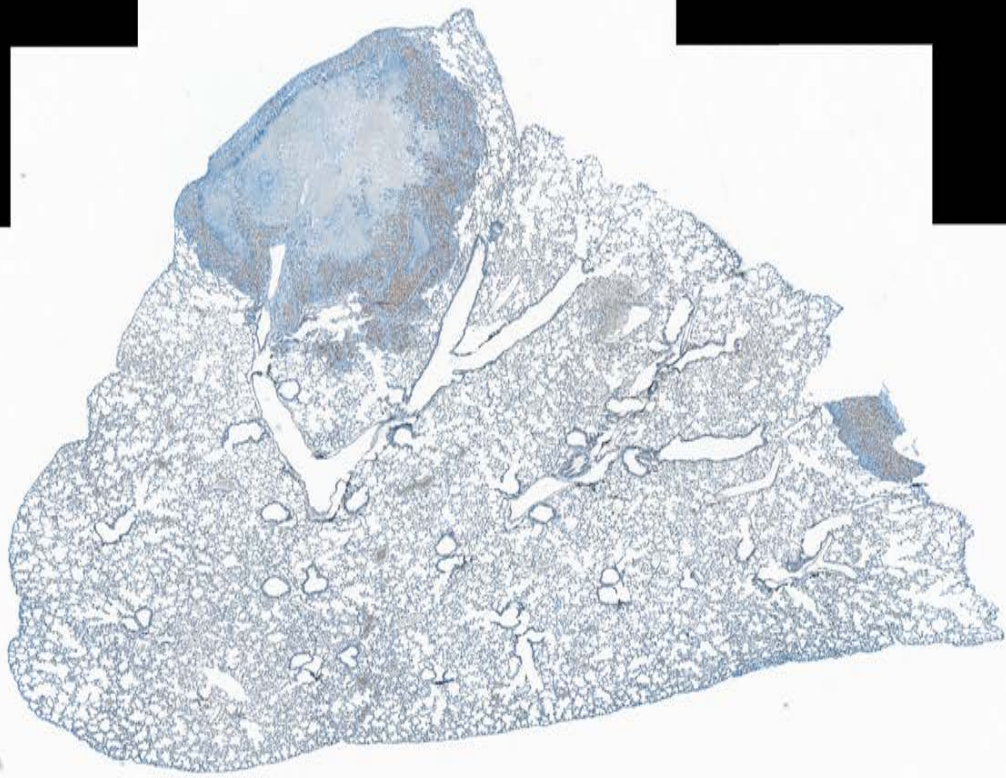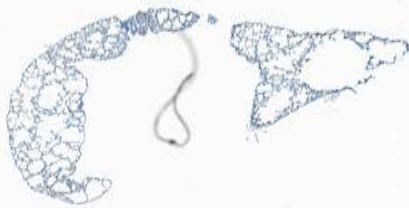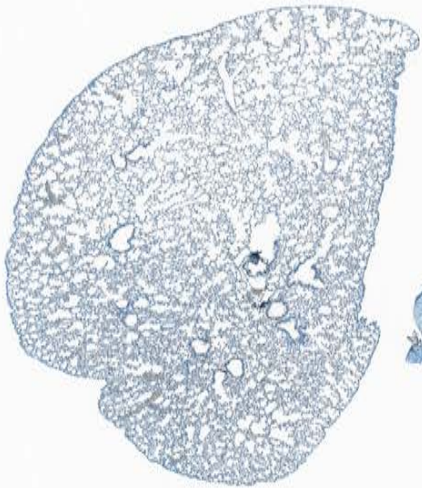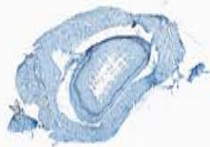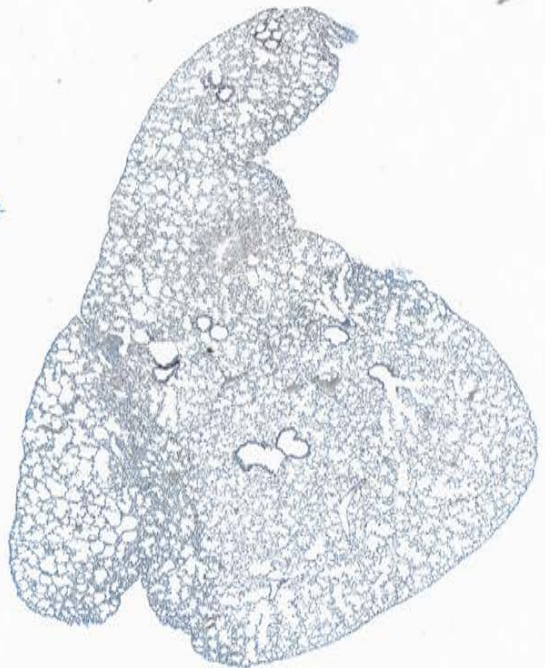

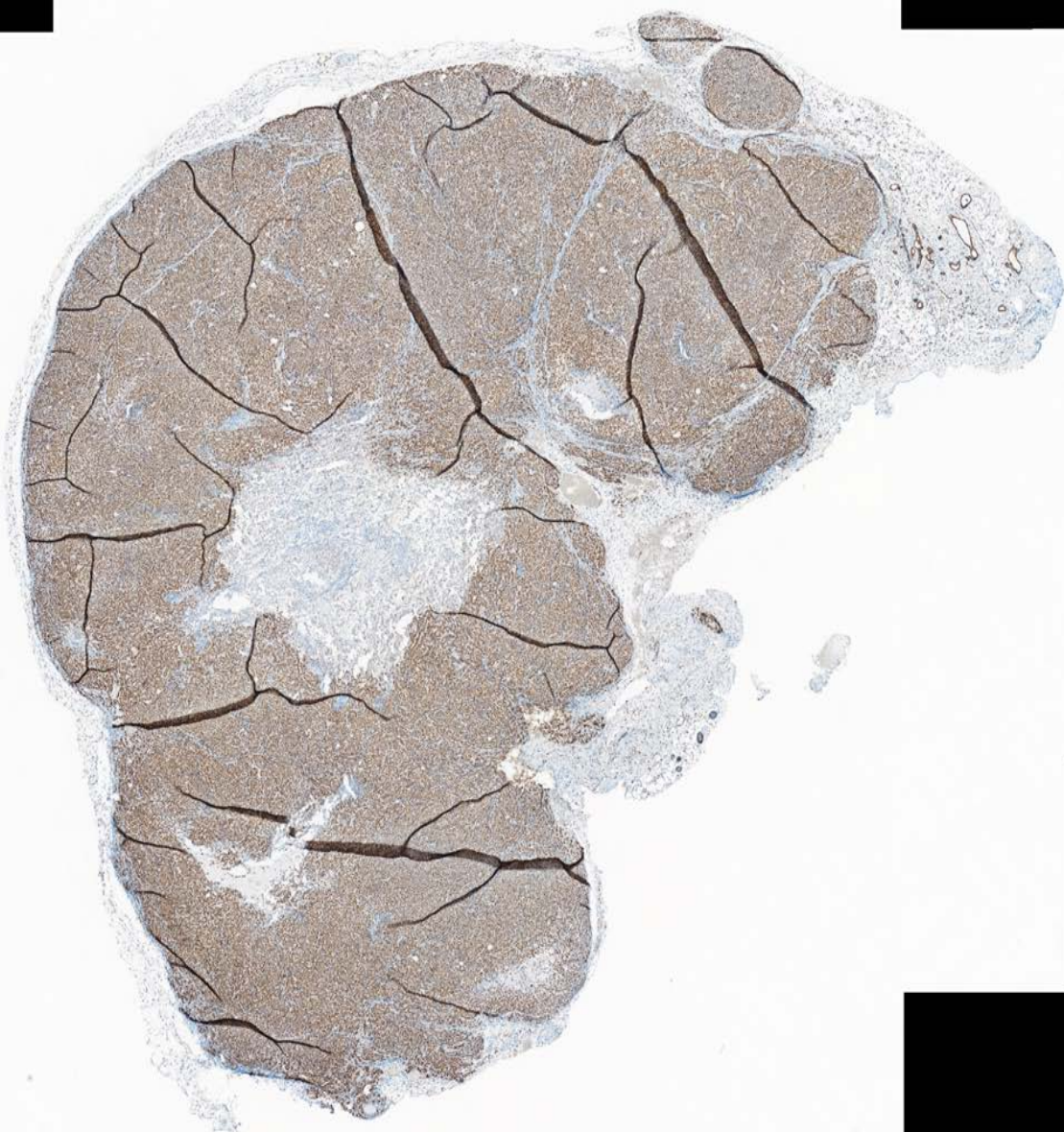

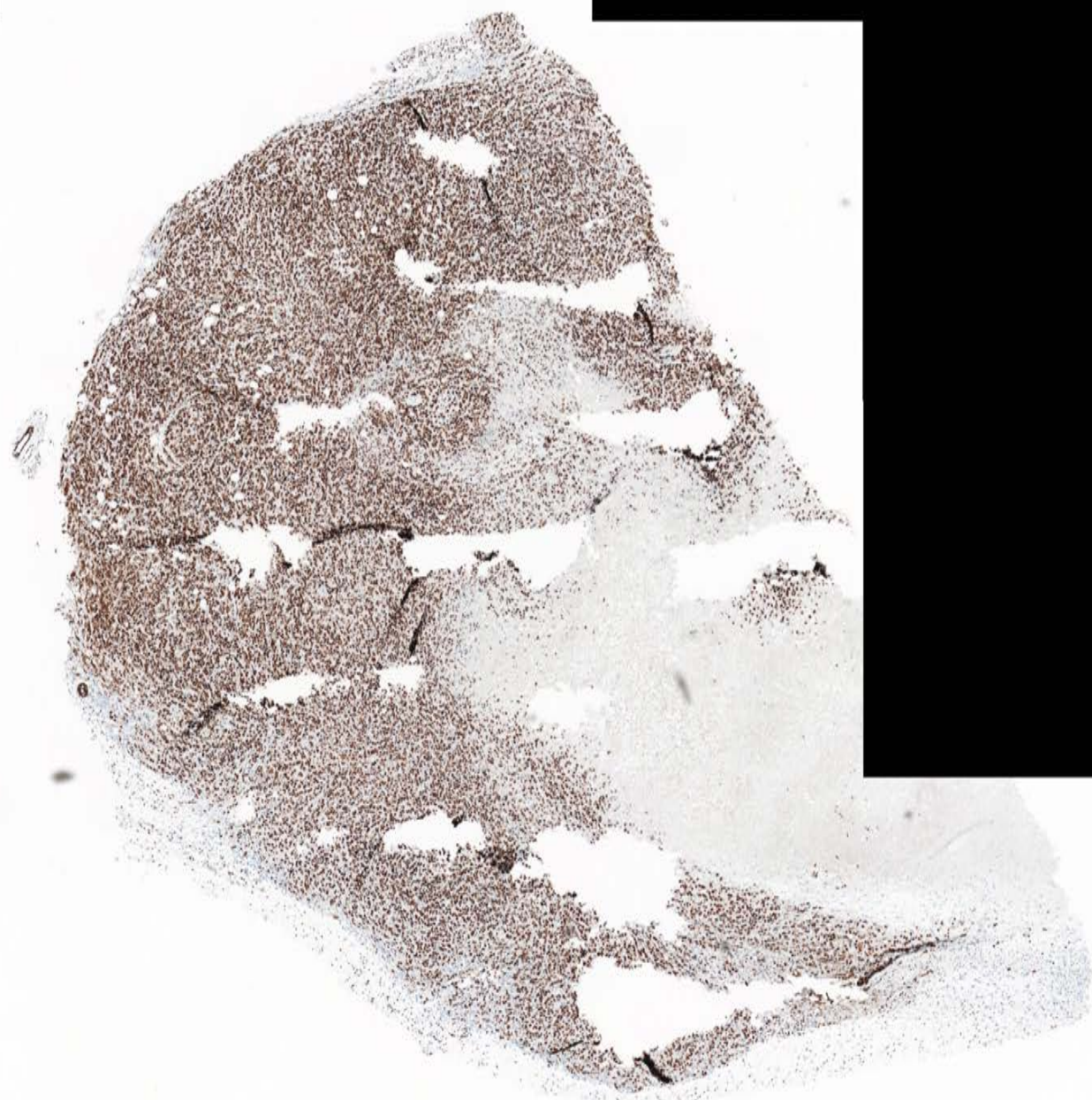

Supplement: Supplementary file 14 — Source Data for Figure 6 [file EMMM-13-e13162-s010.zip › SourceDataFor_Figure6_NFIB.pdf]

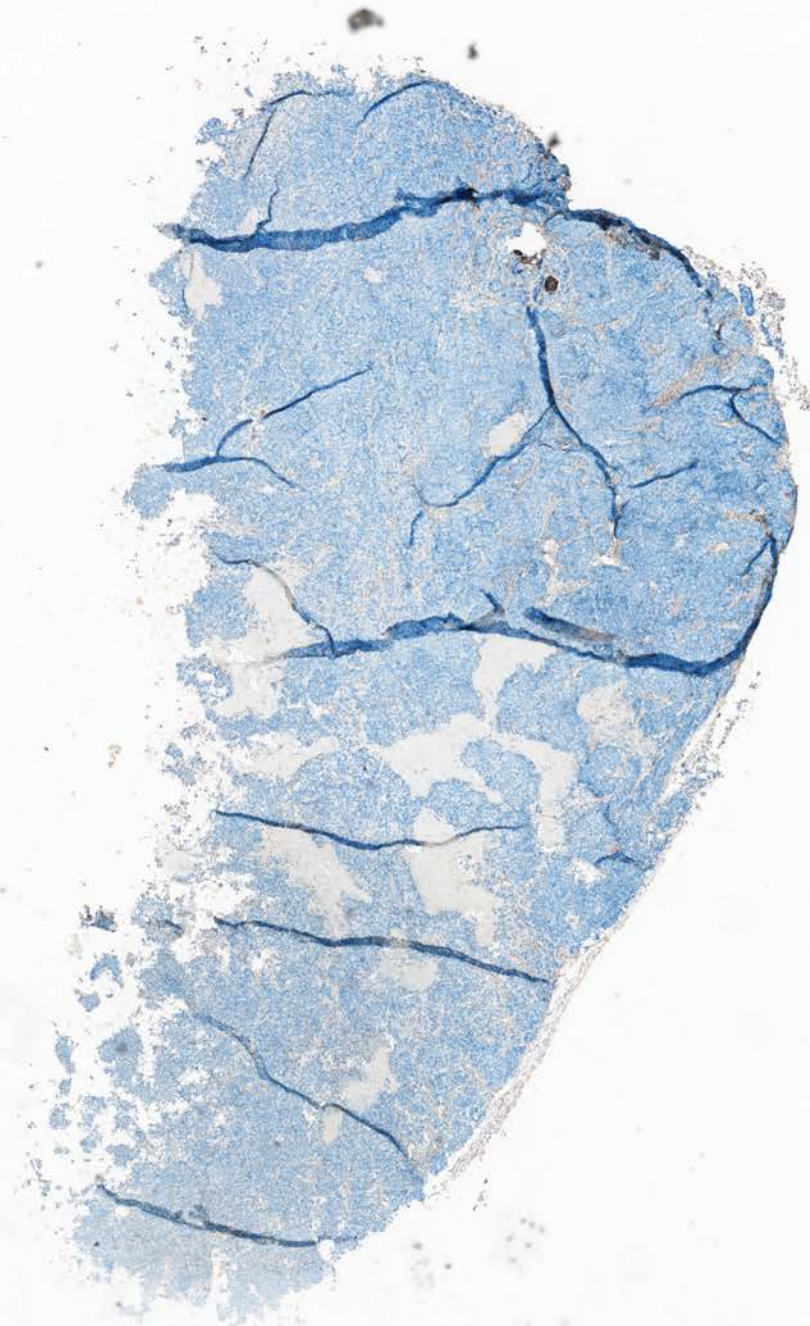

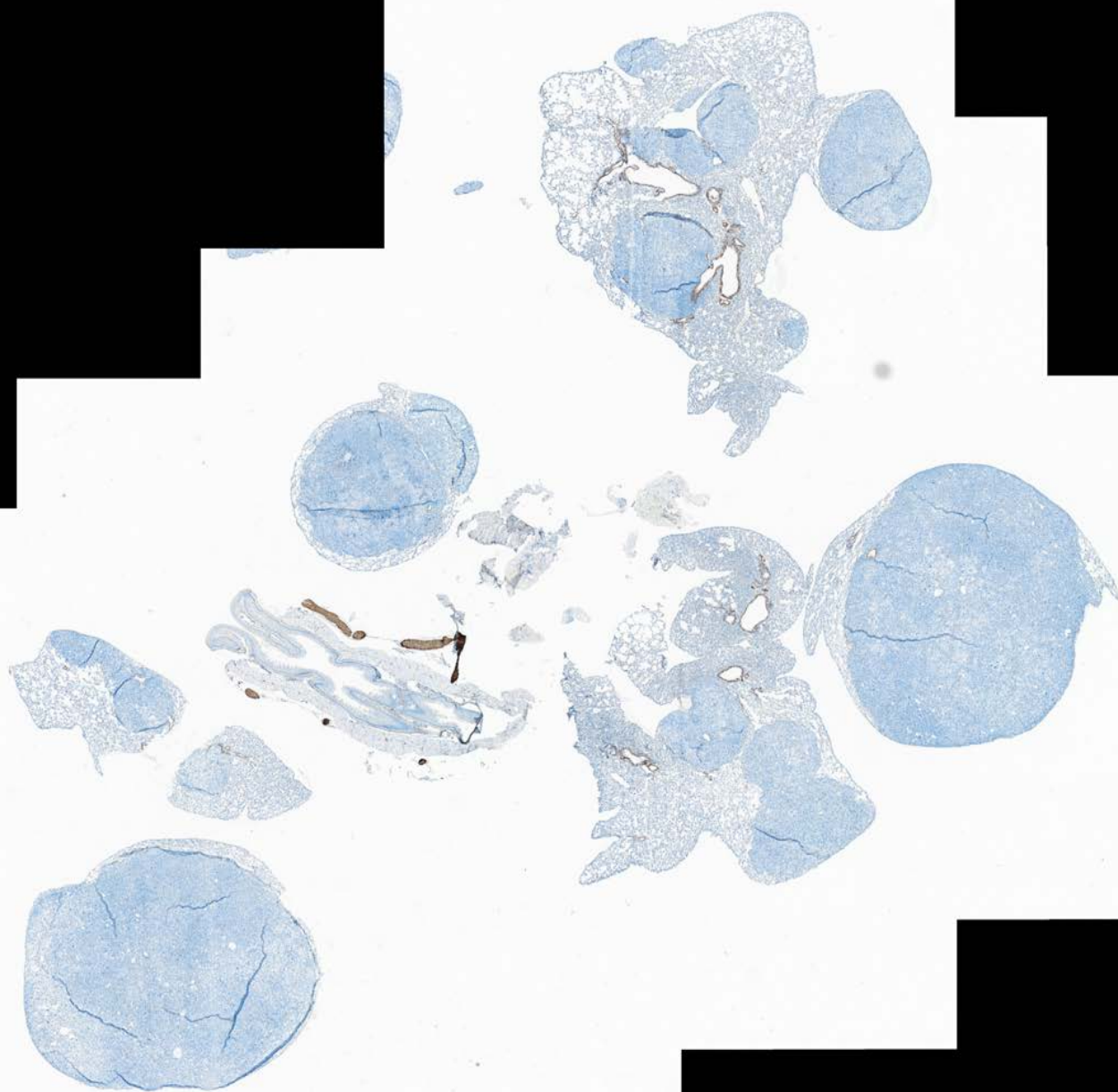

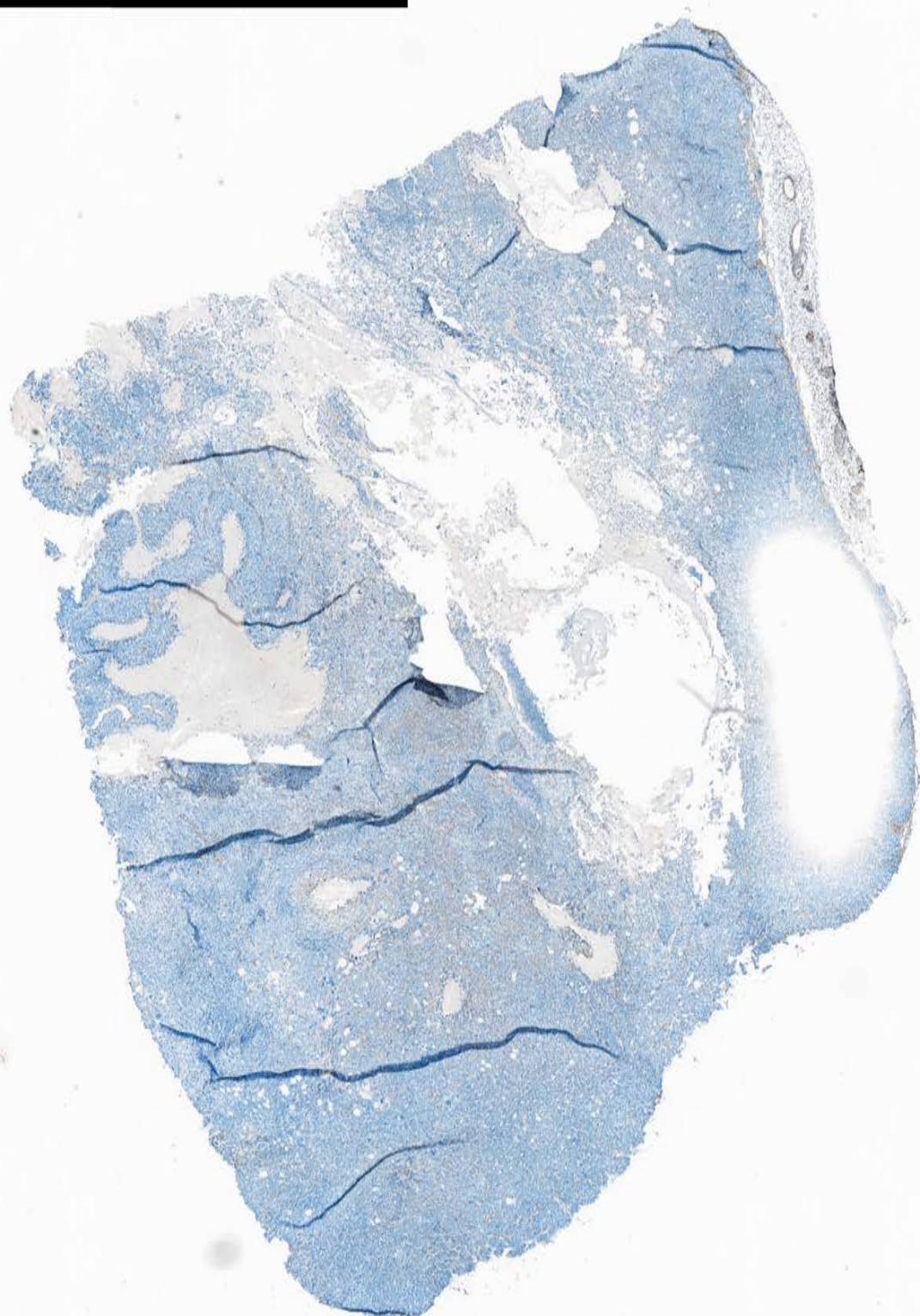

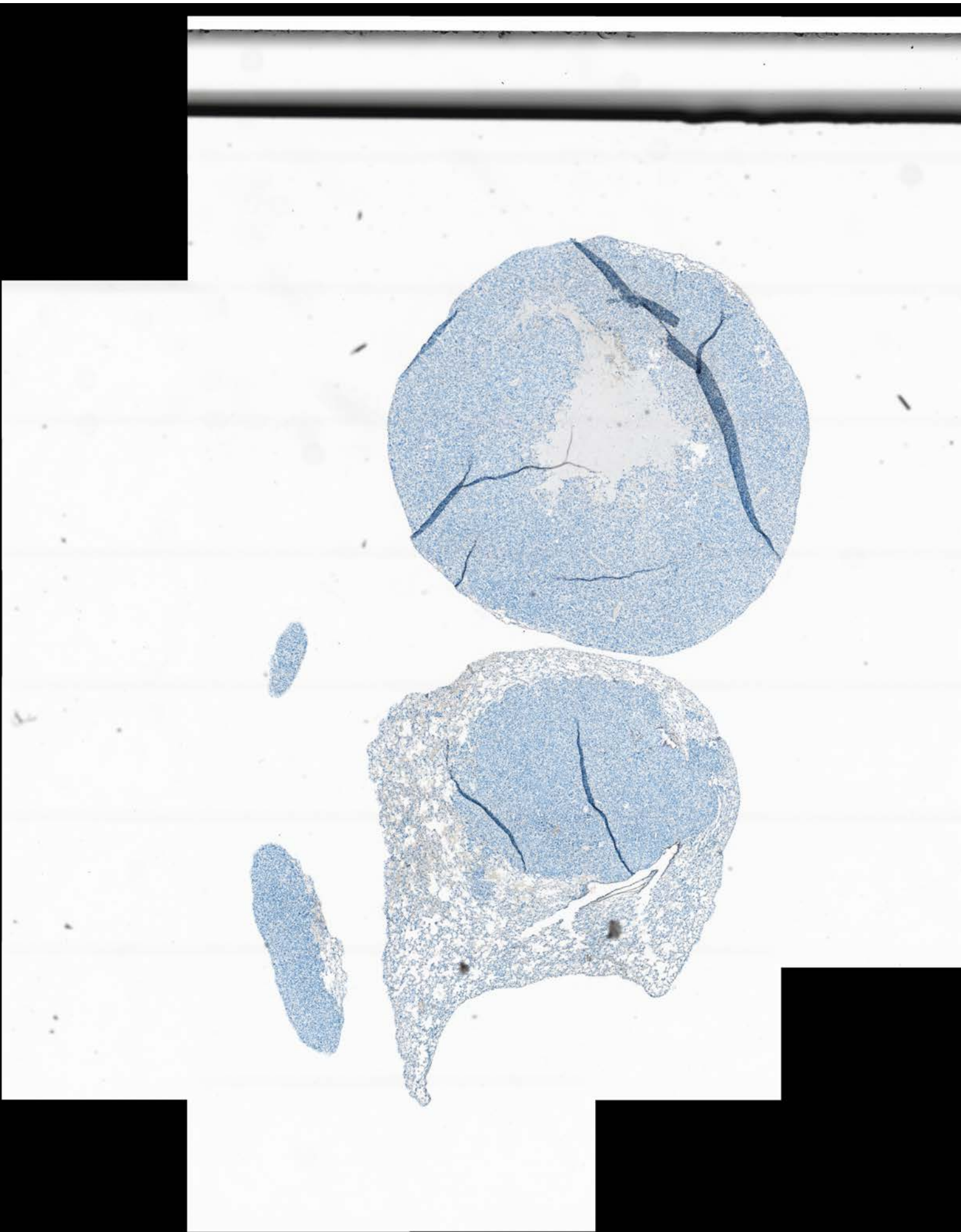



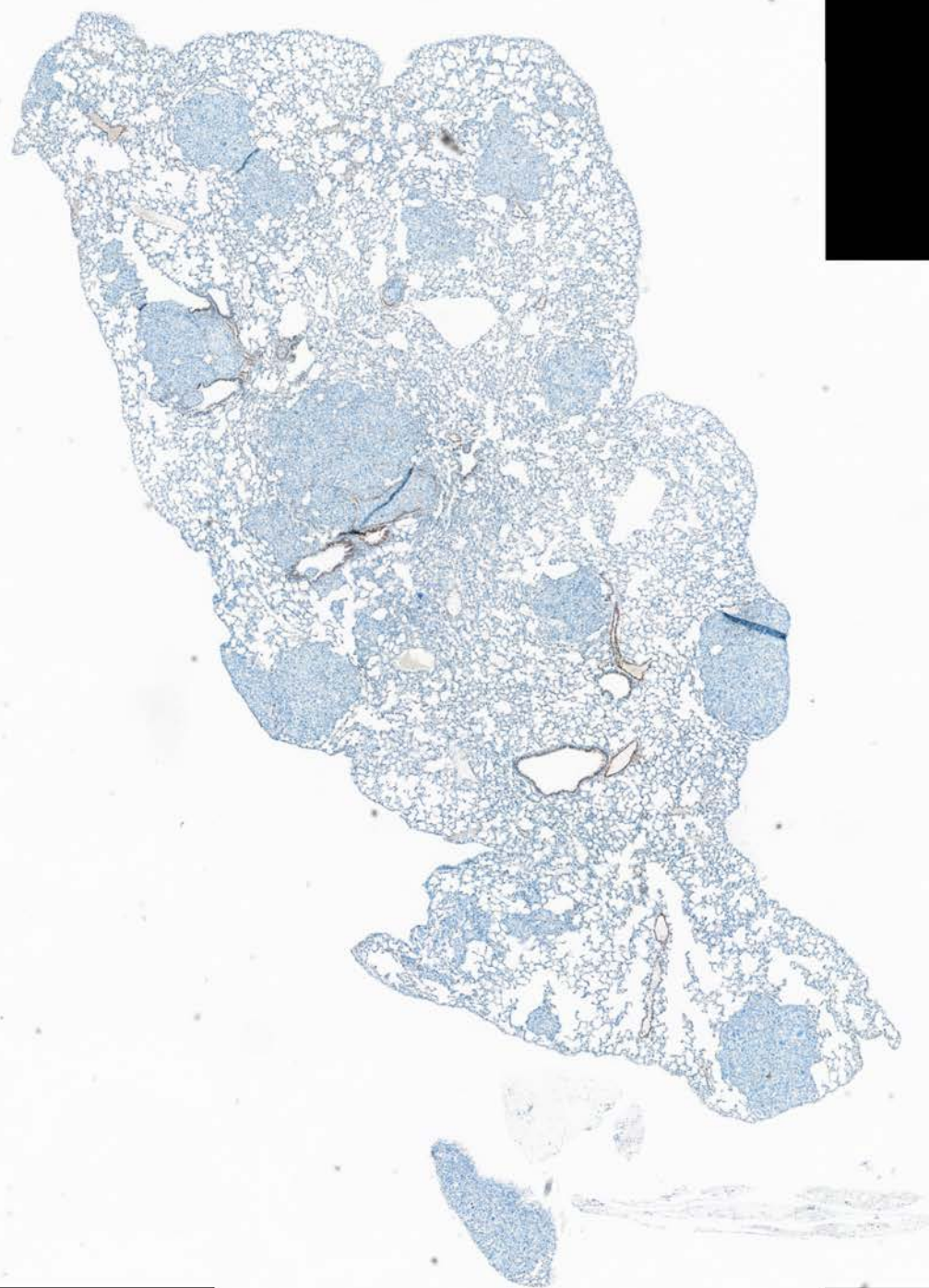

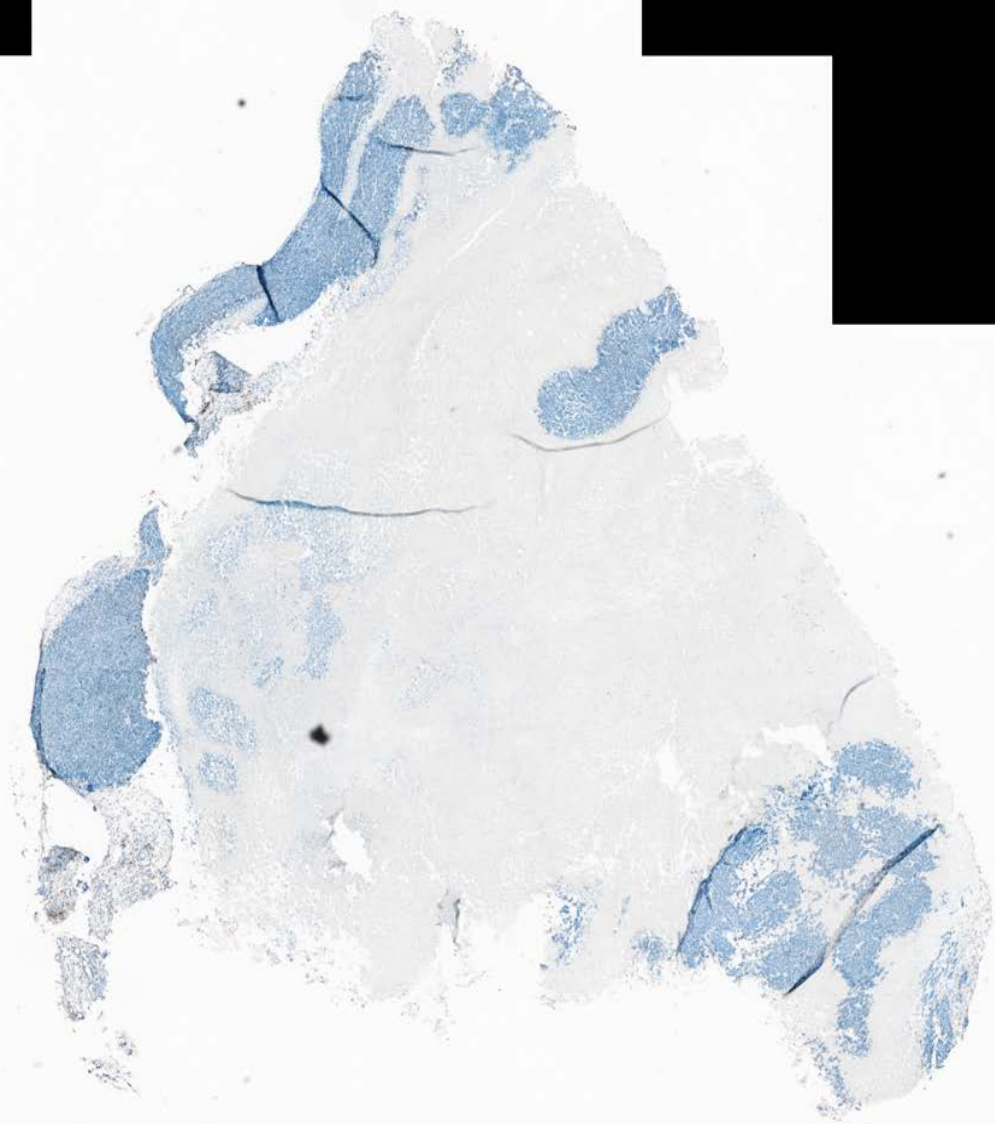

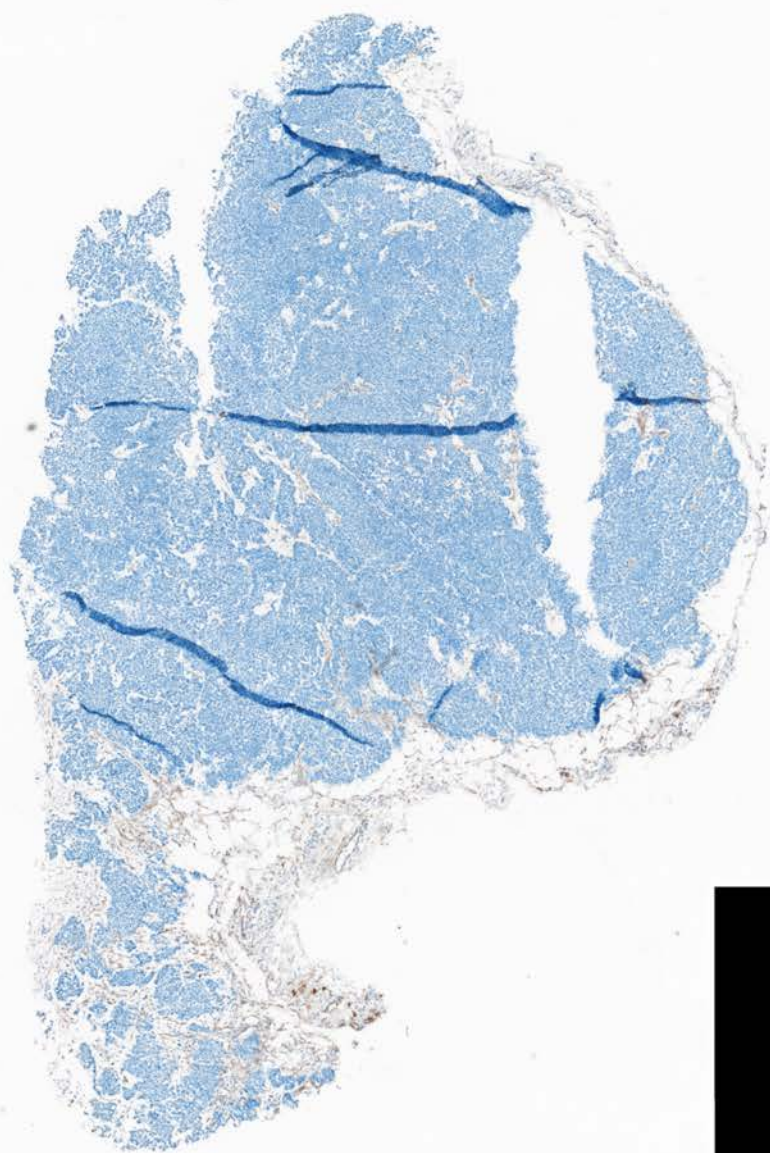

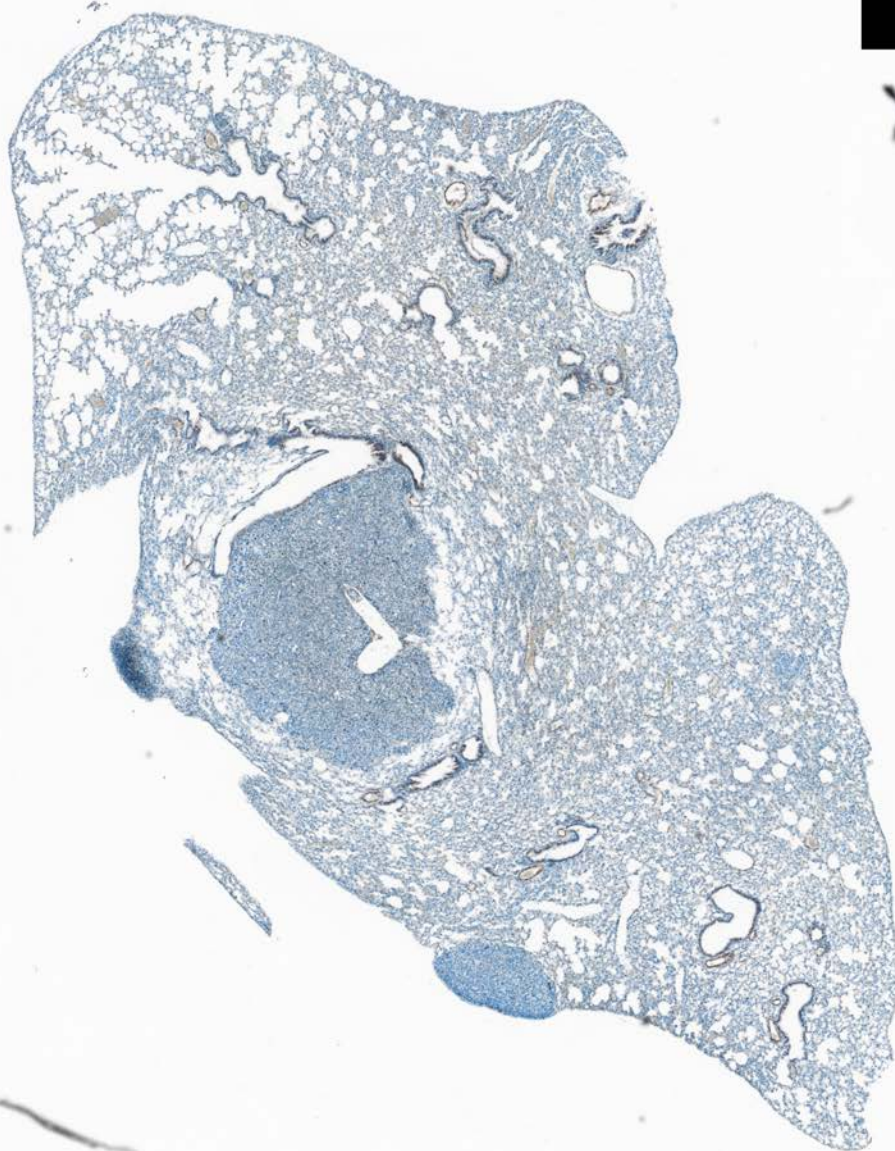

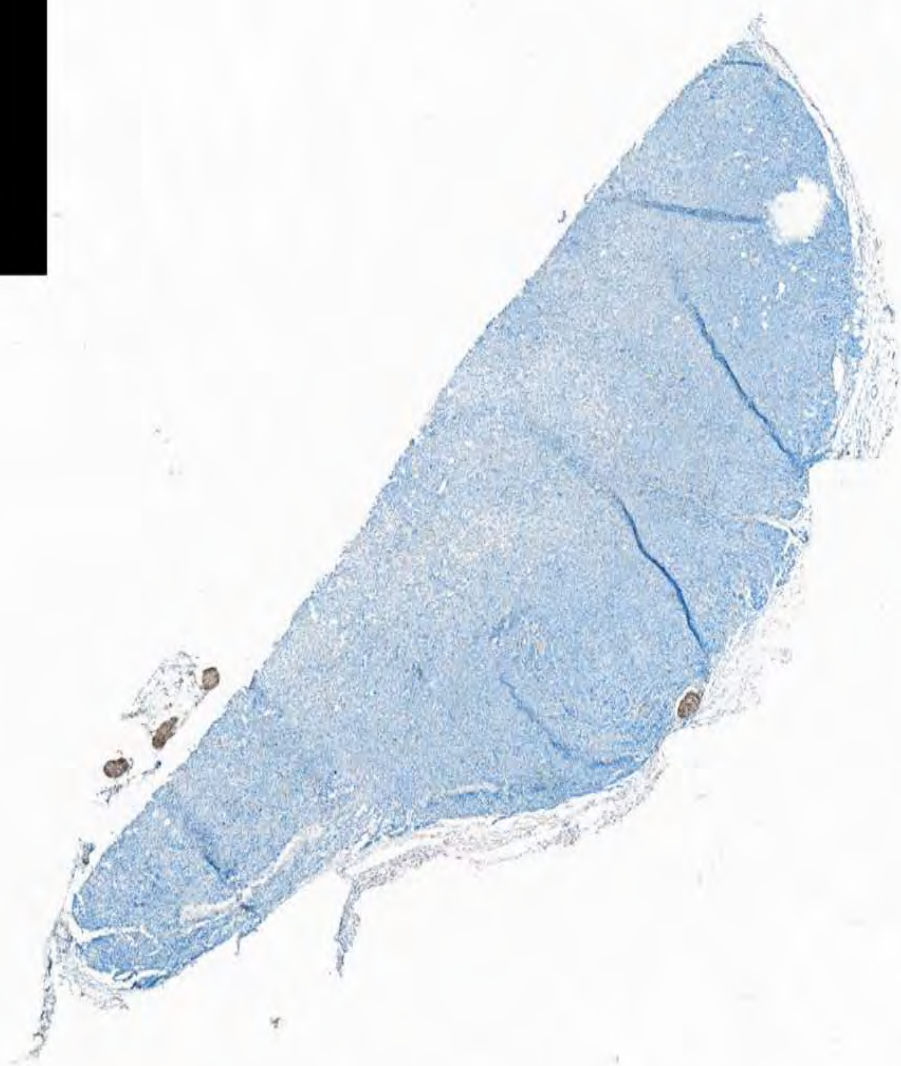

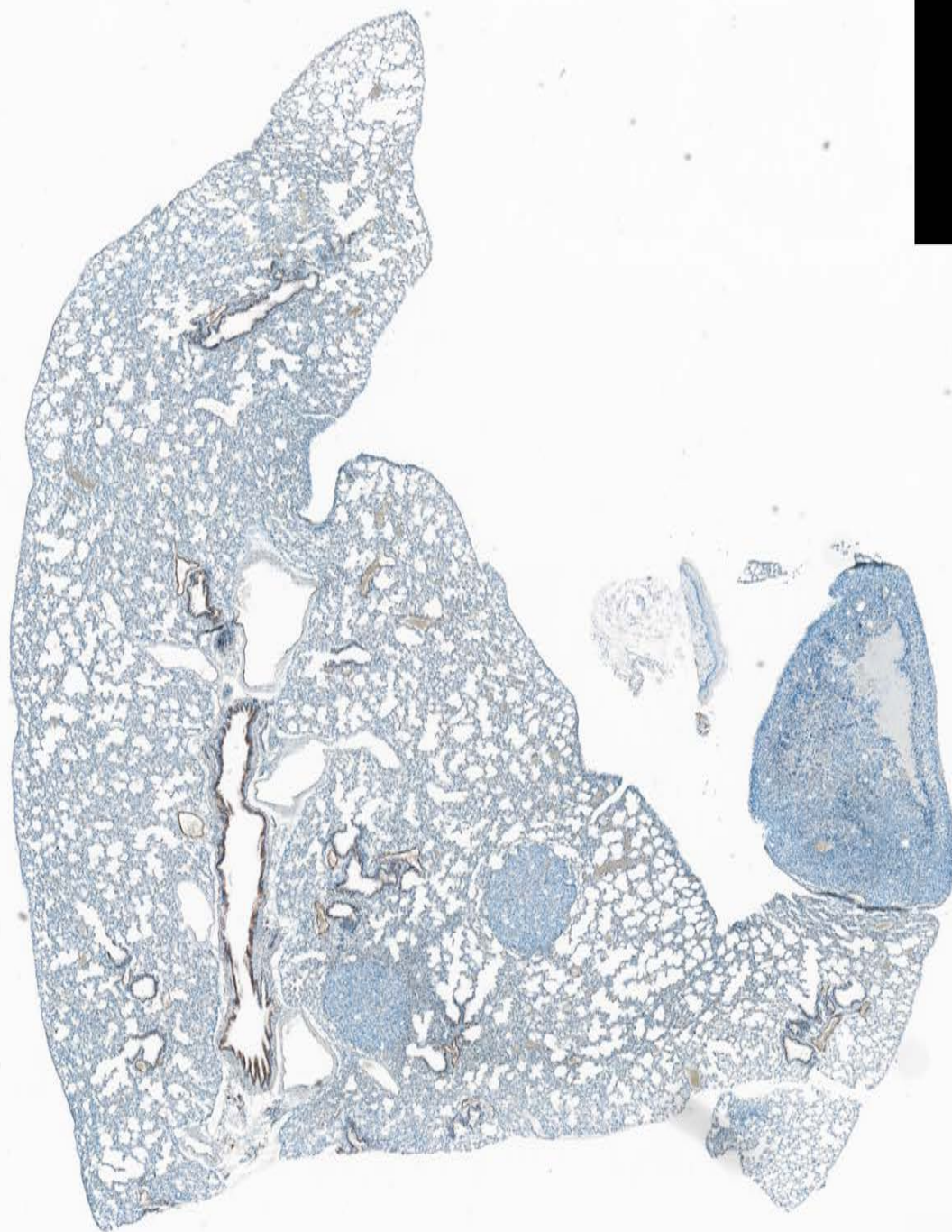

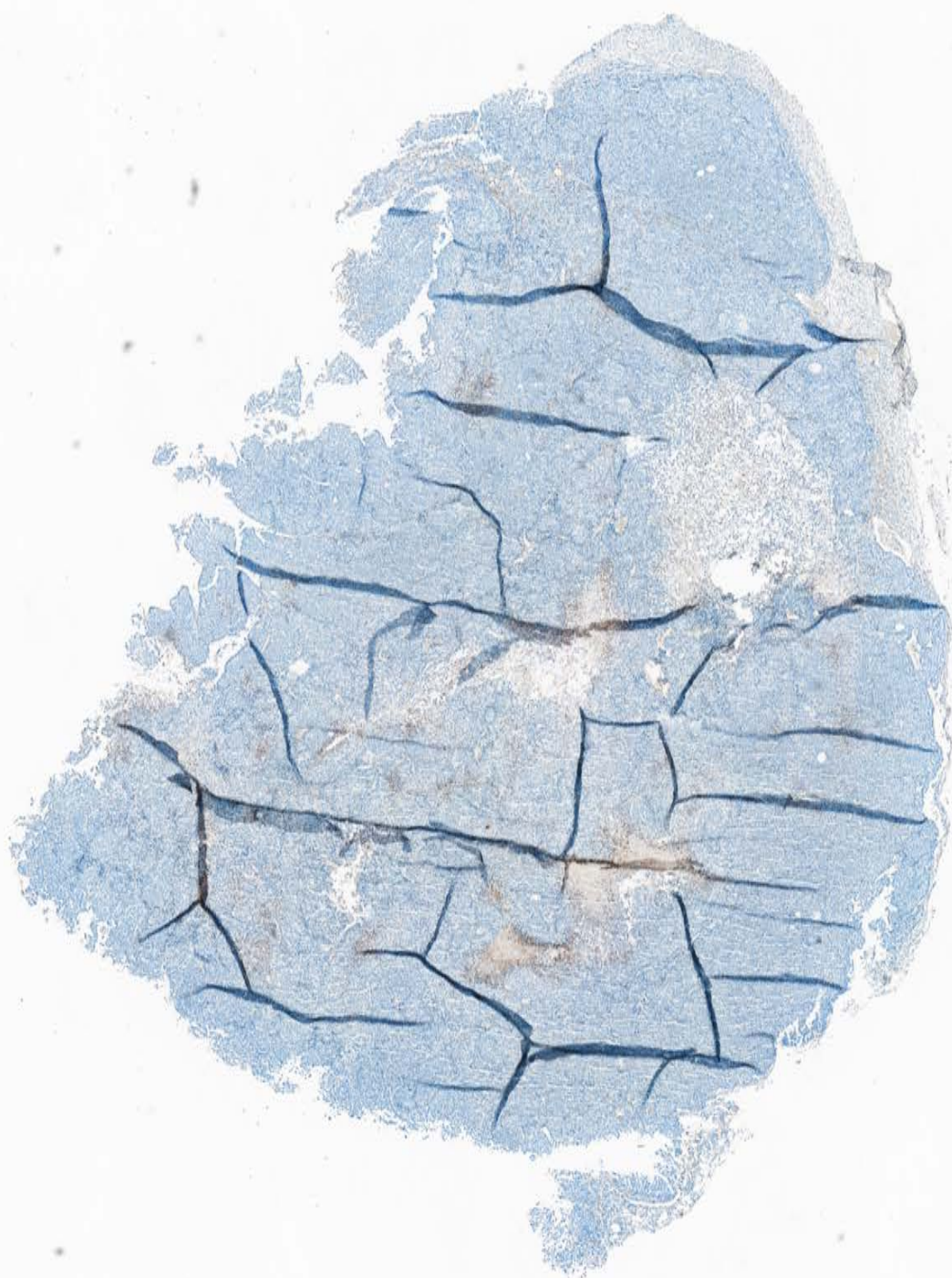

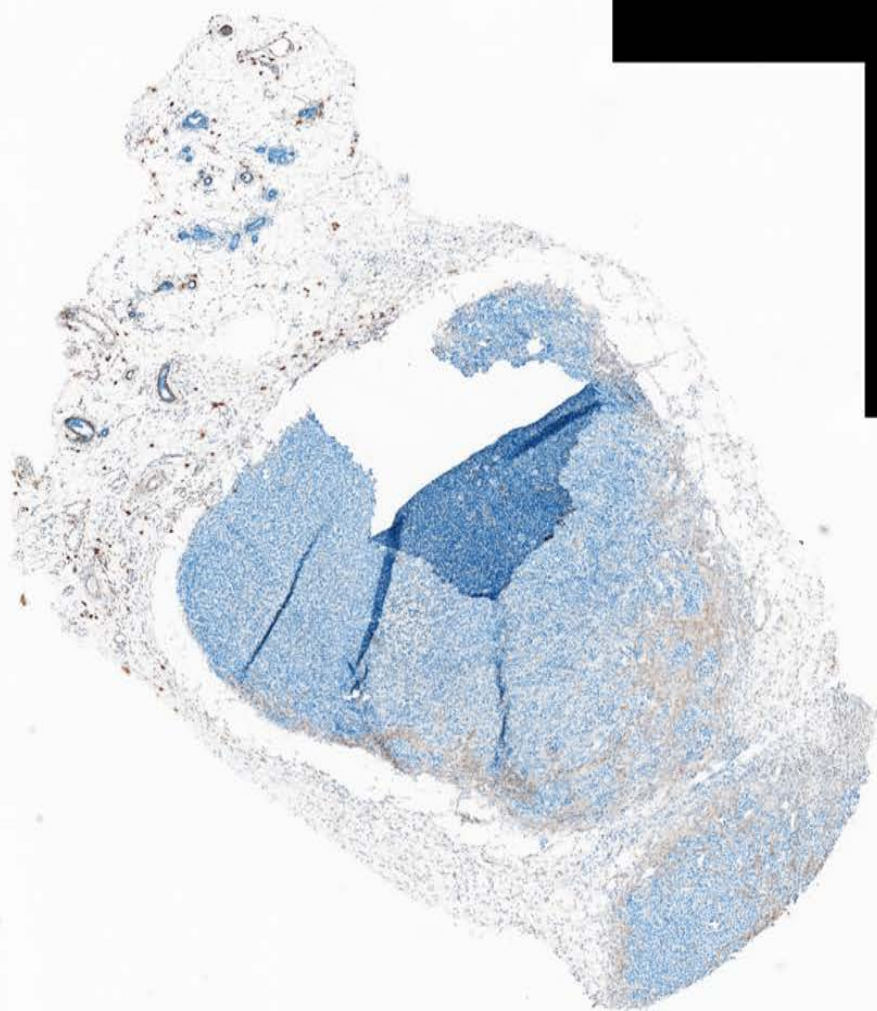

Supplement: Supplementary file 14 — Source Data for Figure 6 [file EMMM-13-e13162-s010.zip › SourceDataFor_Figure6_VEGFA.pdf]
