# Supplementary material for: The NFIB‐ERO1A axis promotes breast cancer metastatic colonization of disseminated tumour cells
Source: EMBO Mol Med. 2021 Mar 10;13(4):e13162. doi: 10.15252/emmm.202013162 (PMC8033524; doi:10.15252/emmm.202013162)

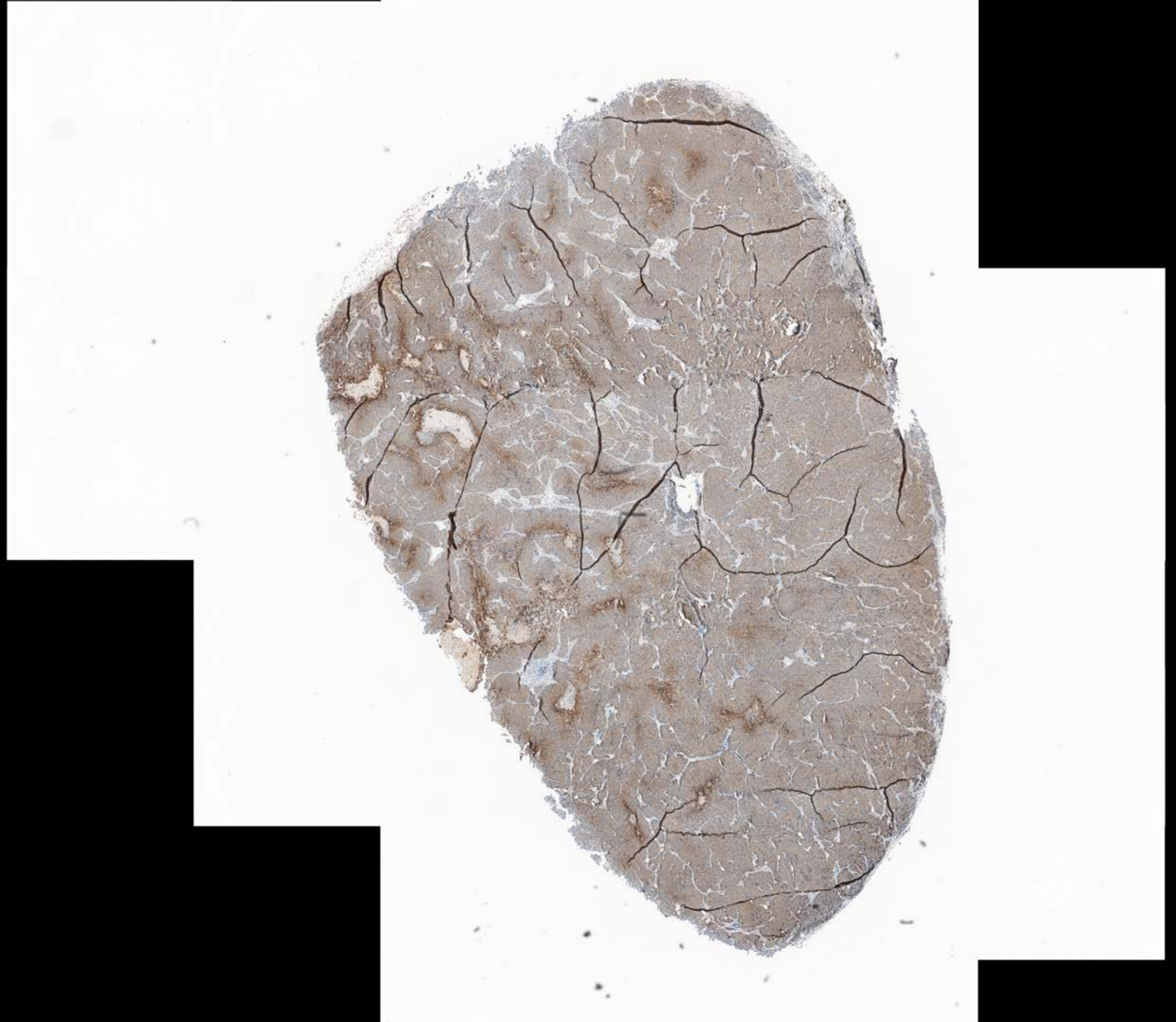

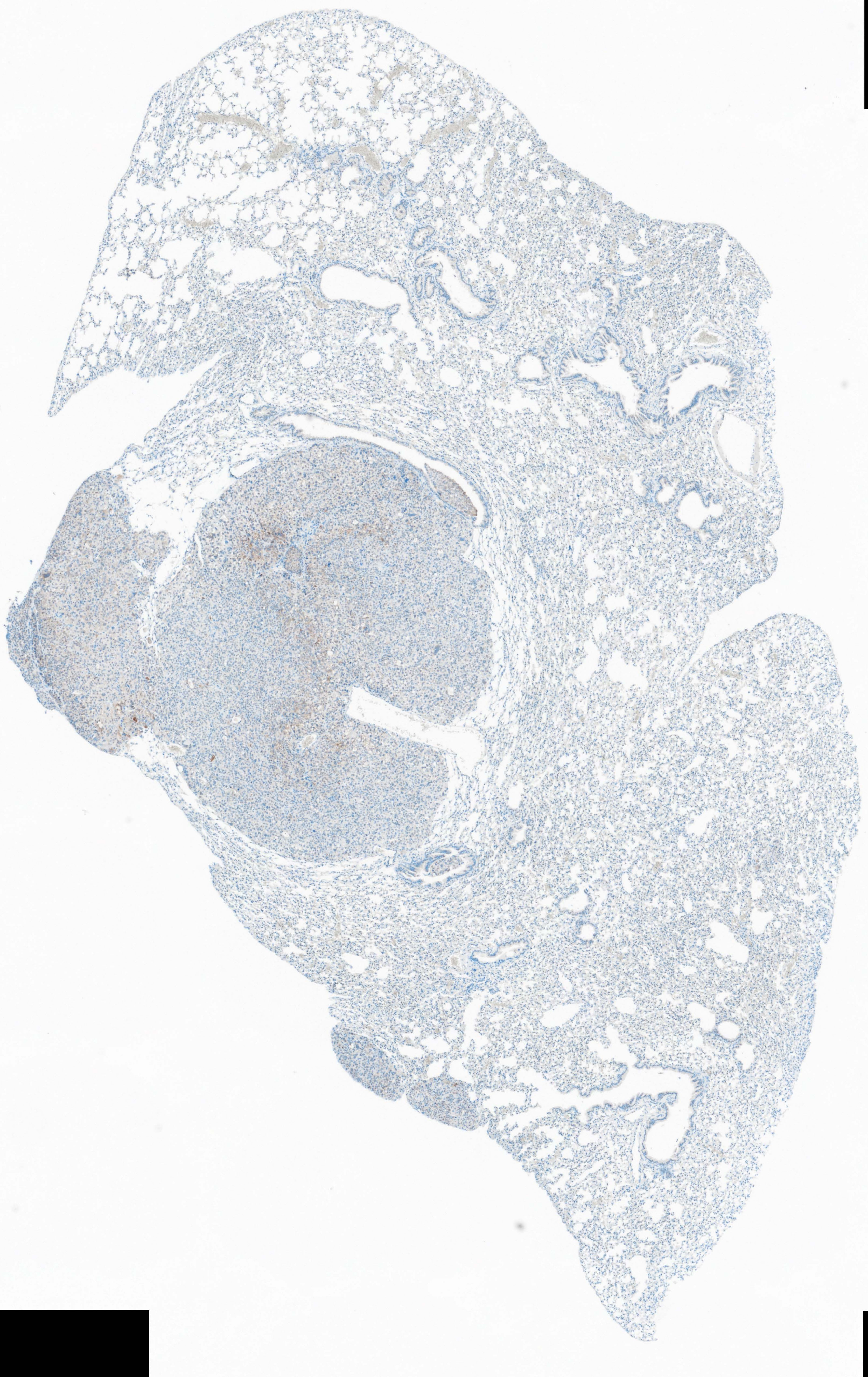

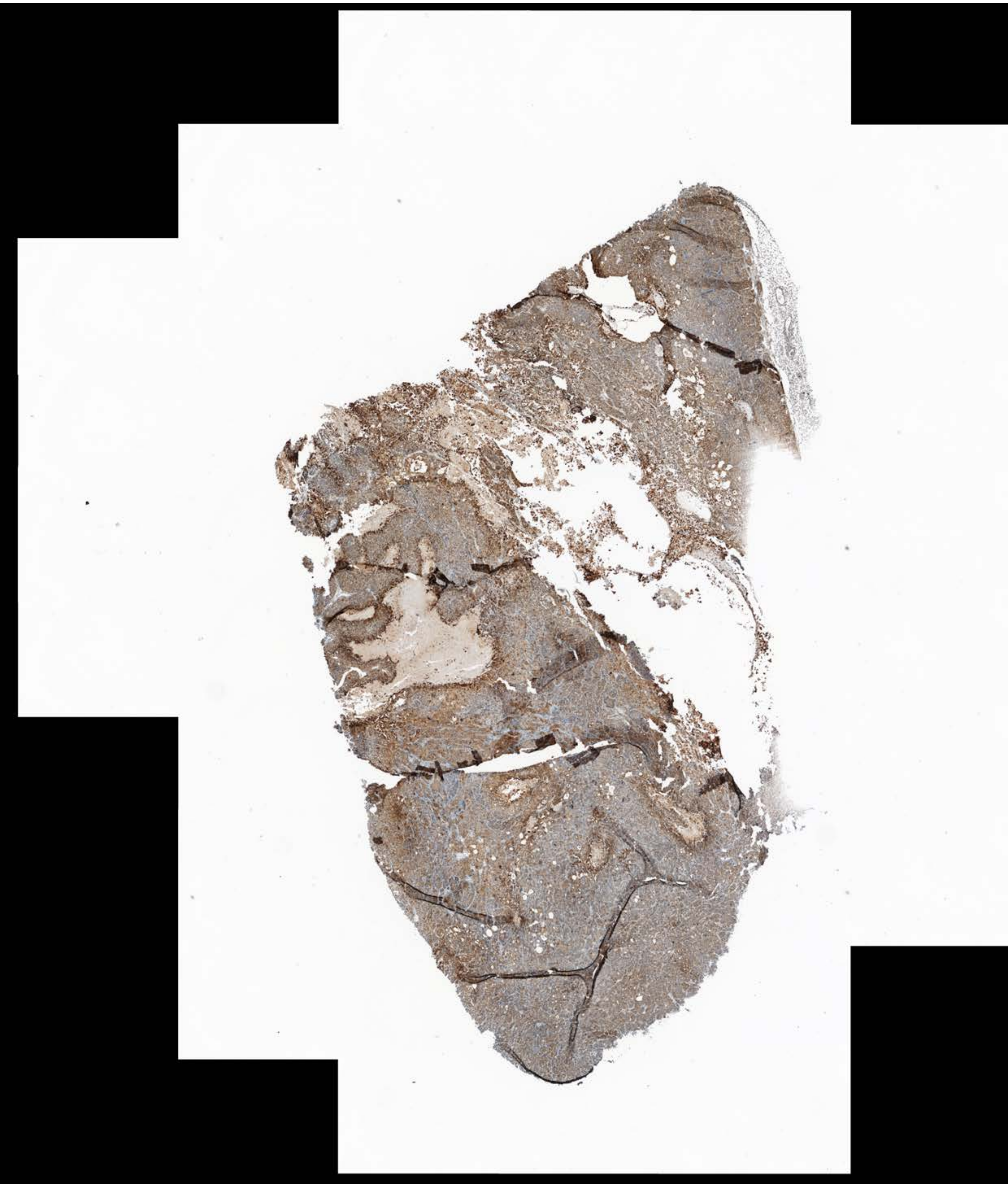

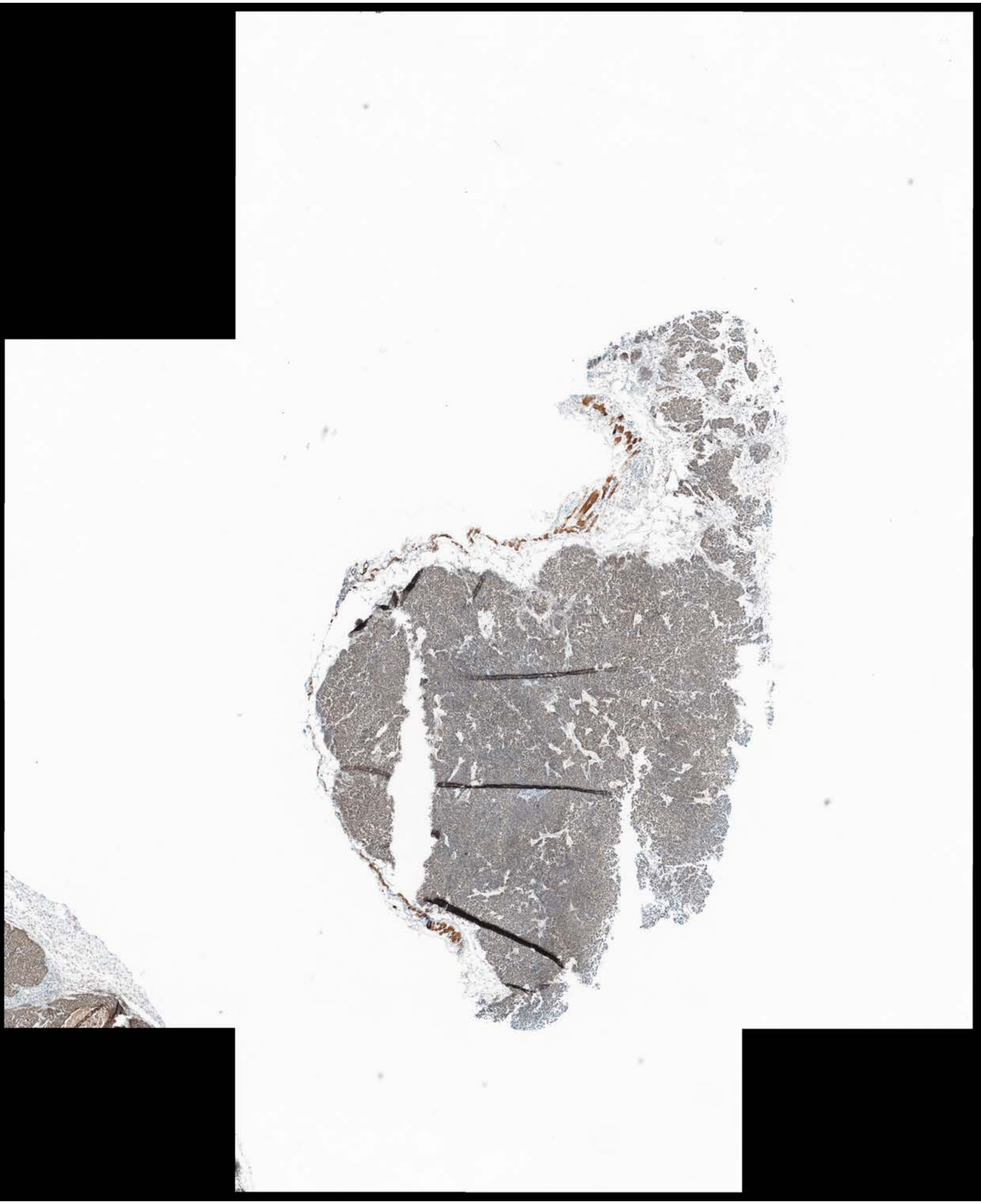

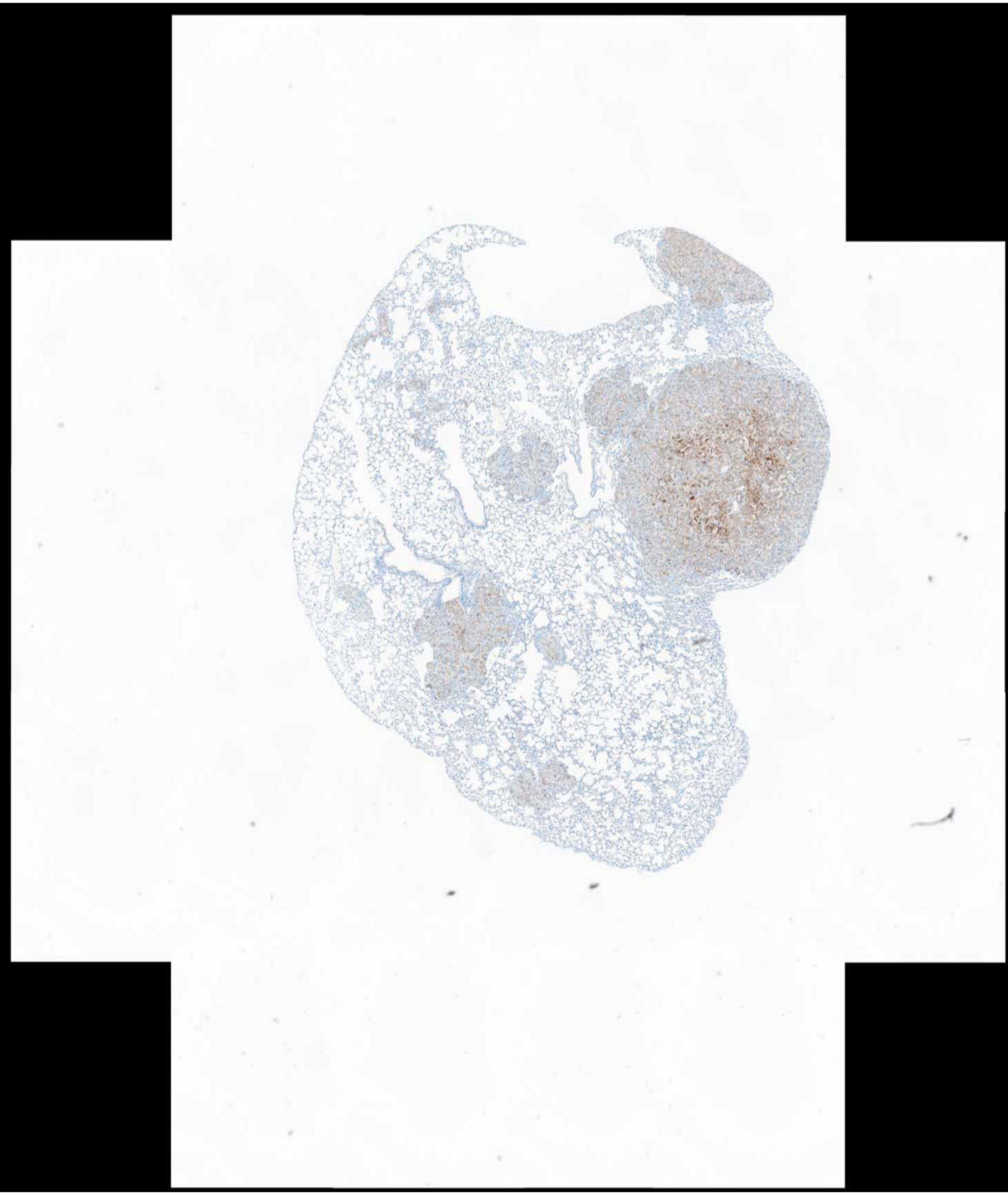

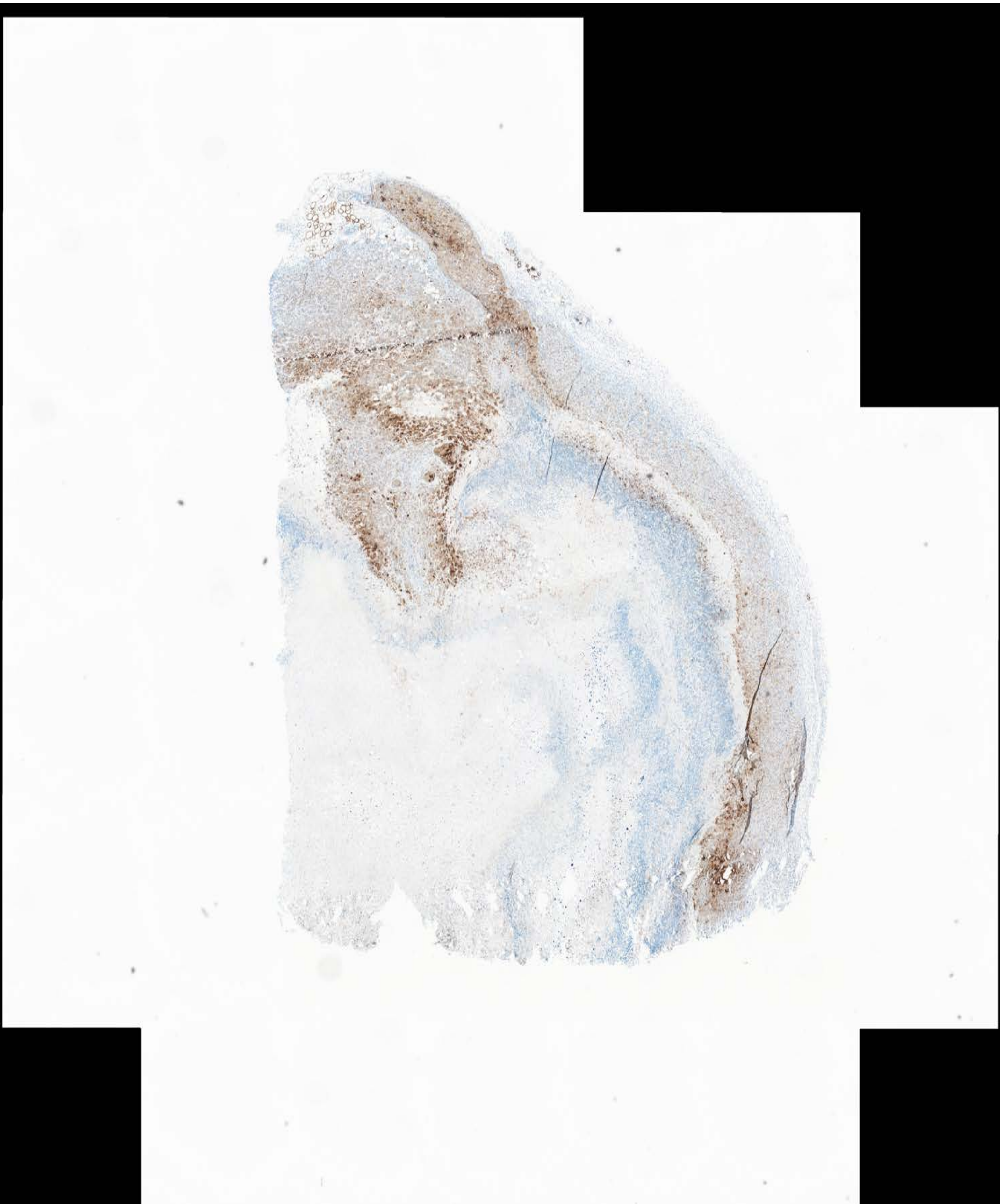

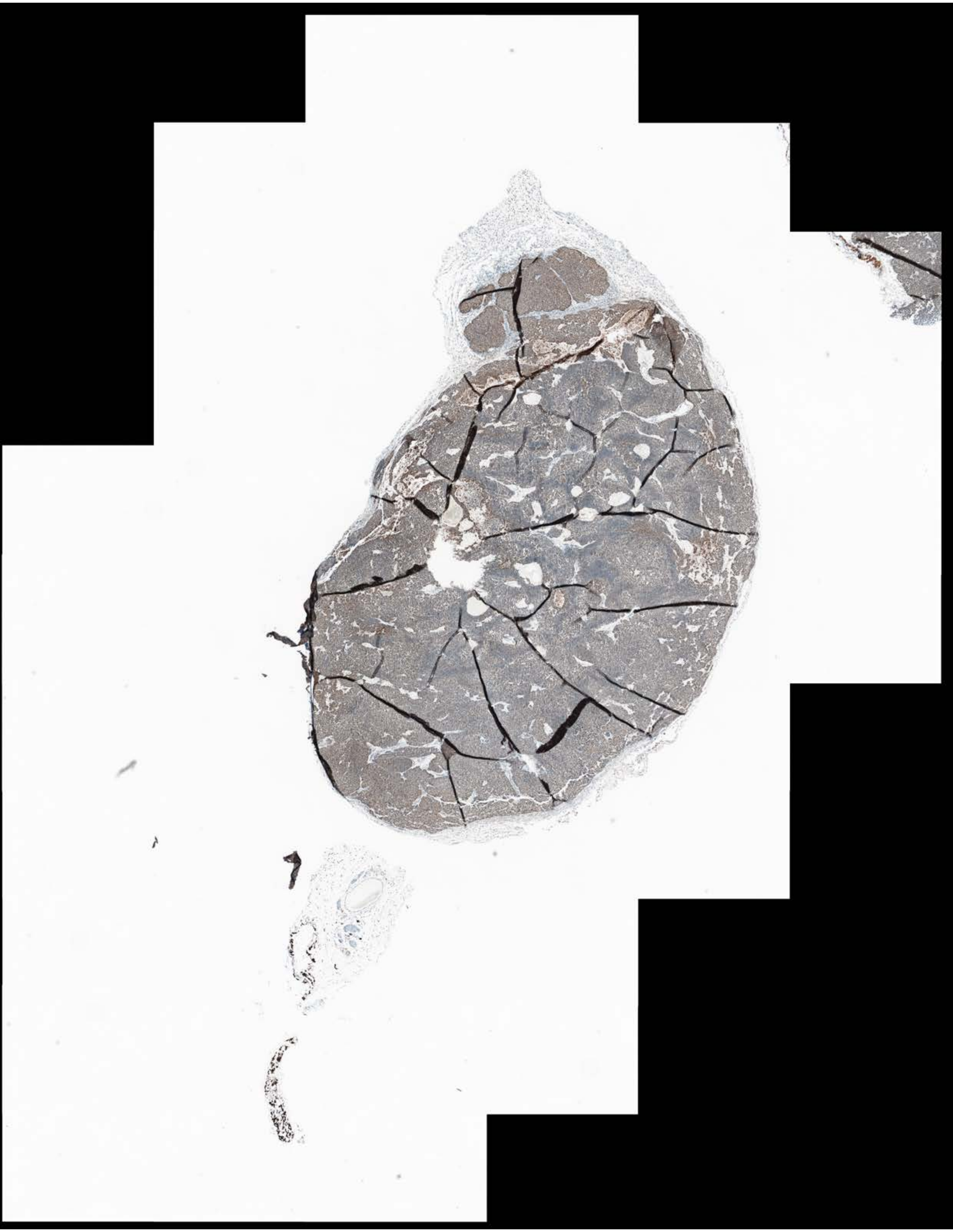

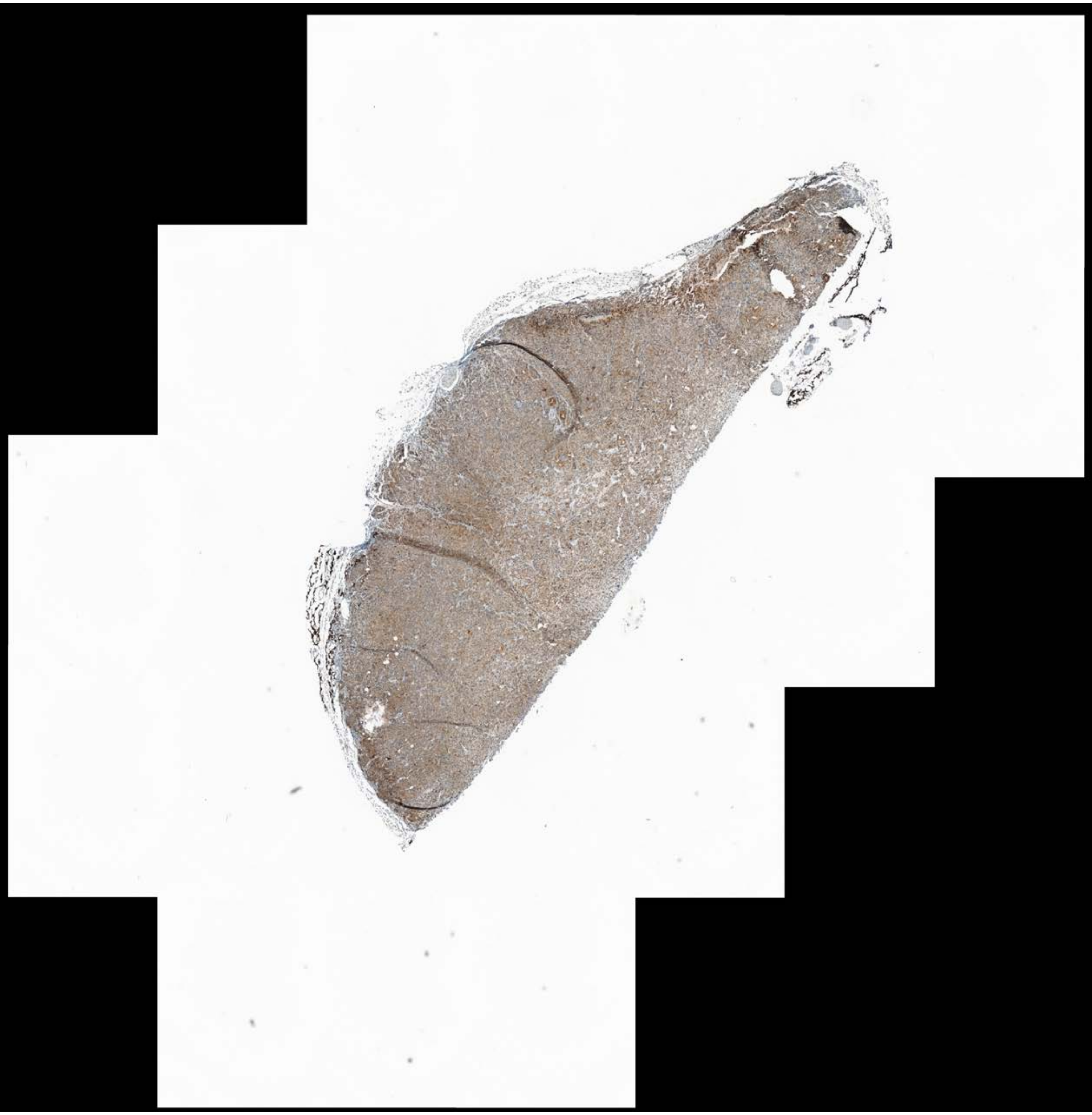

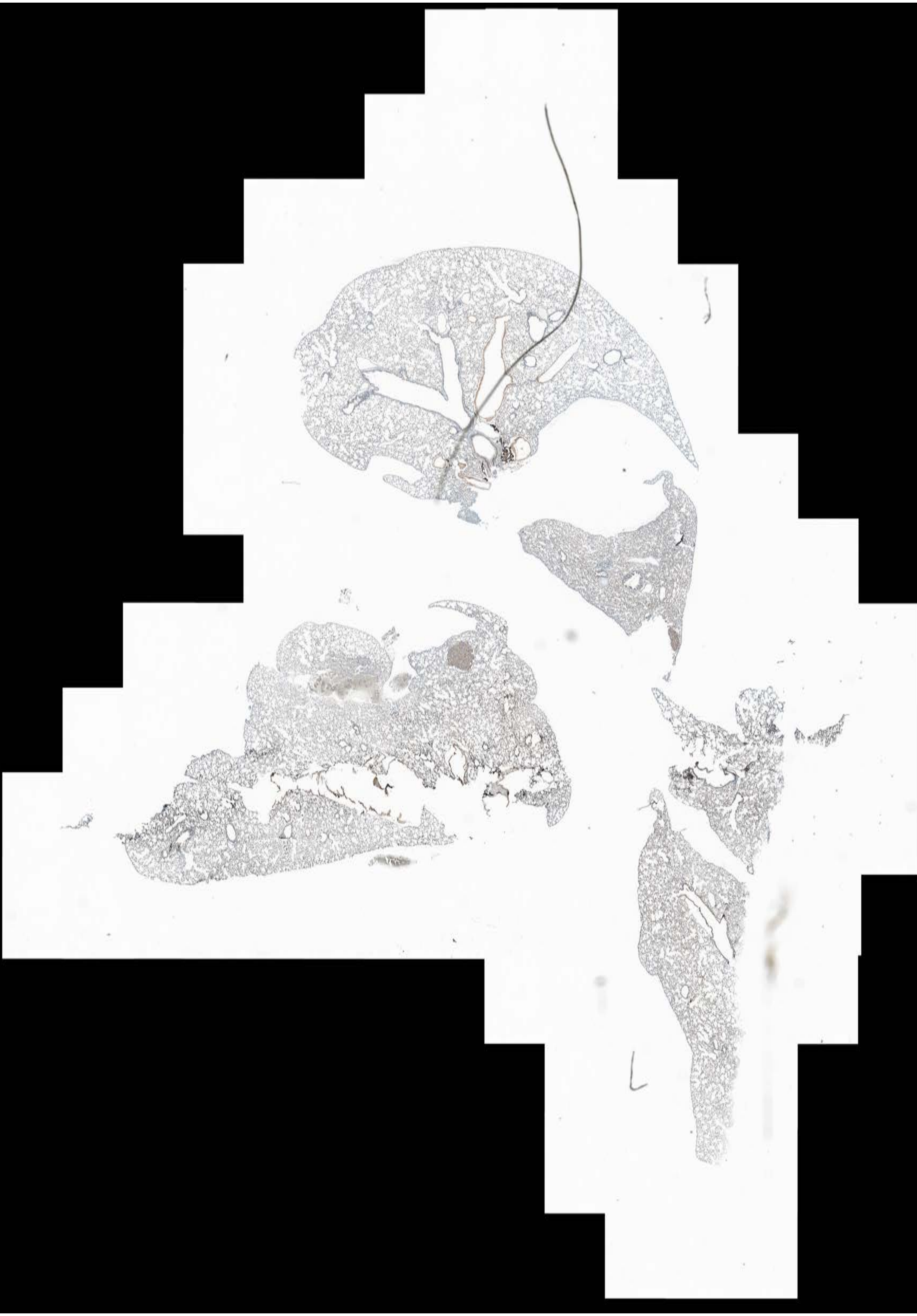

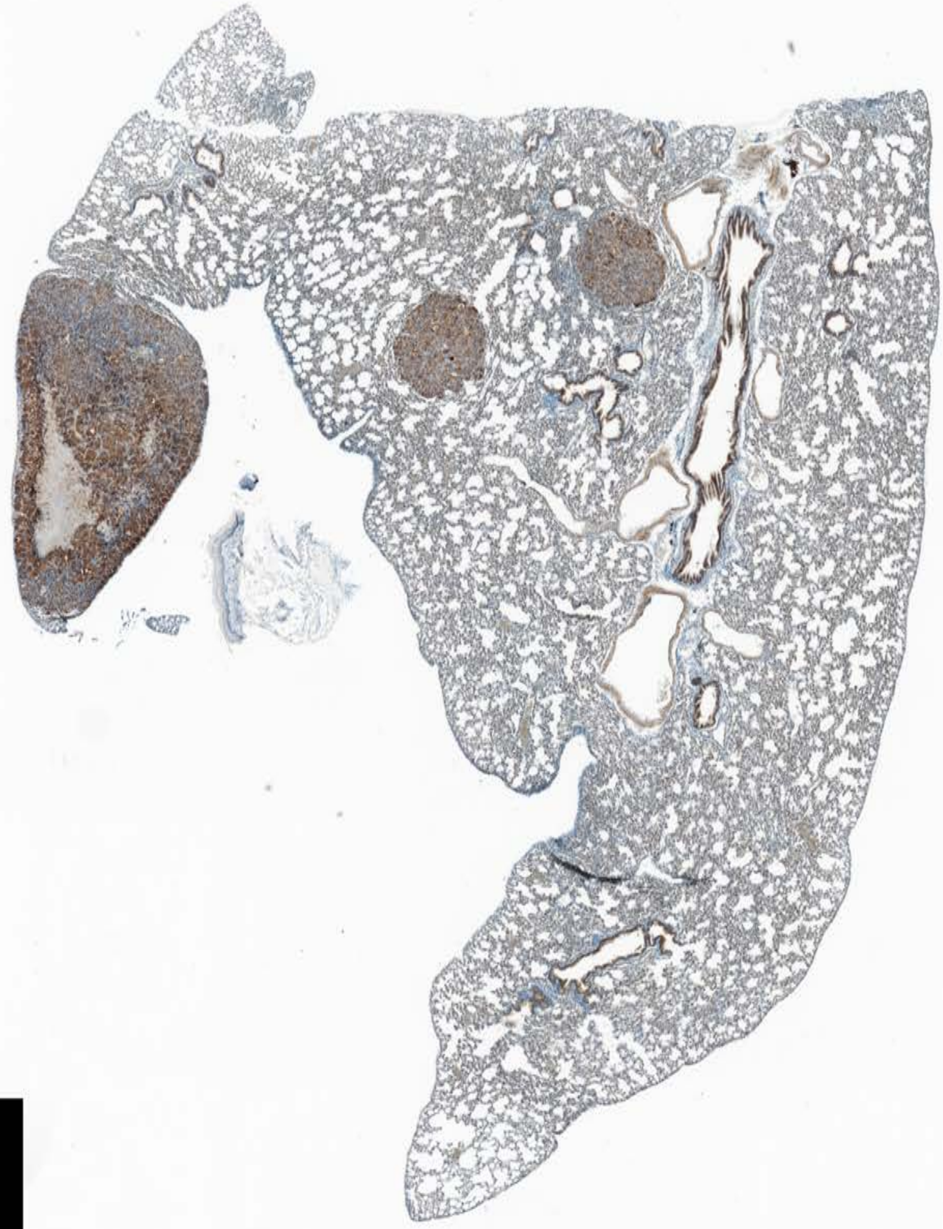

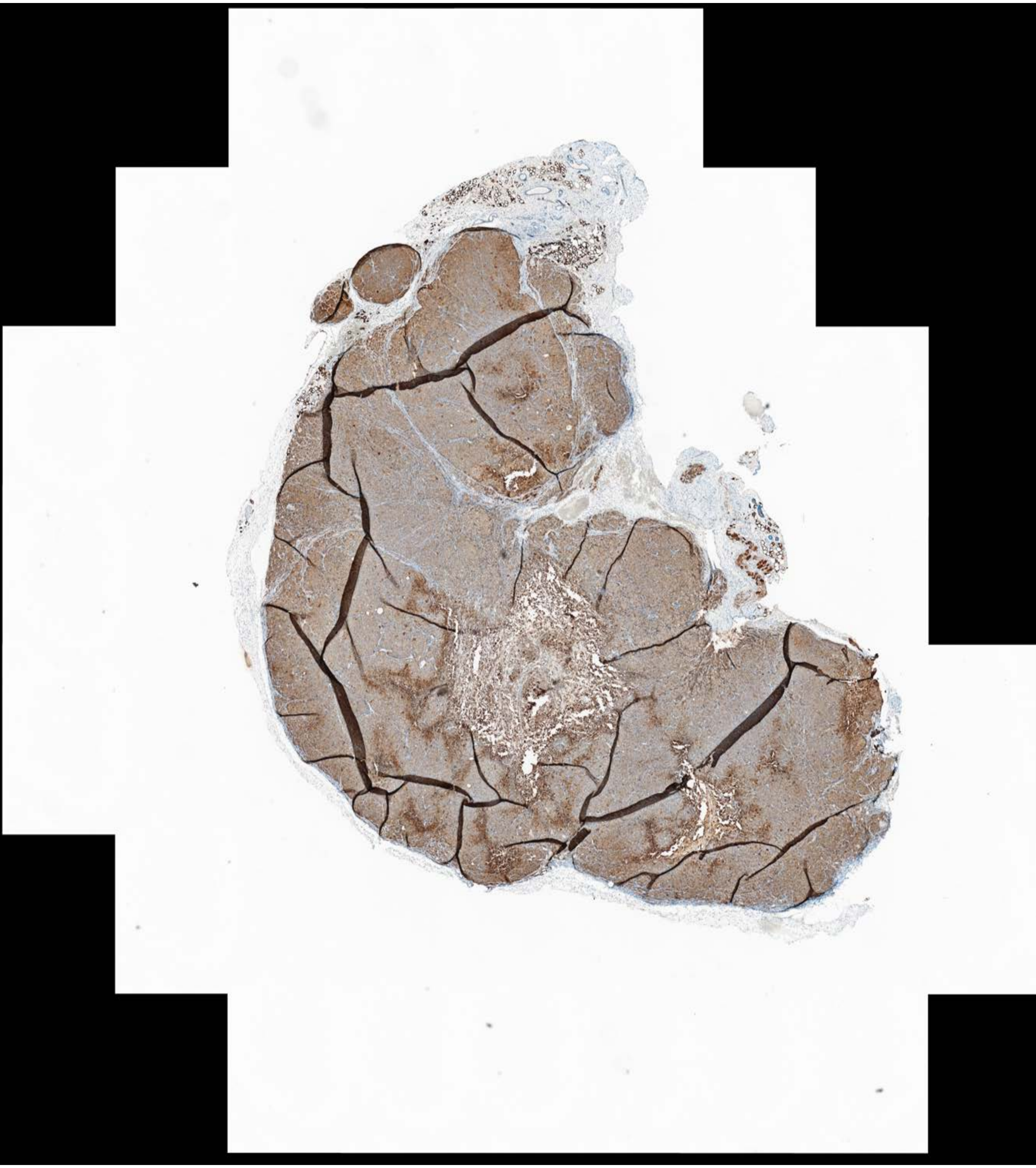

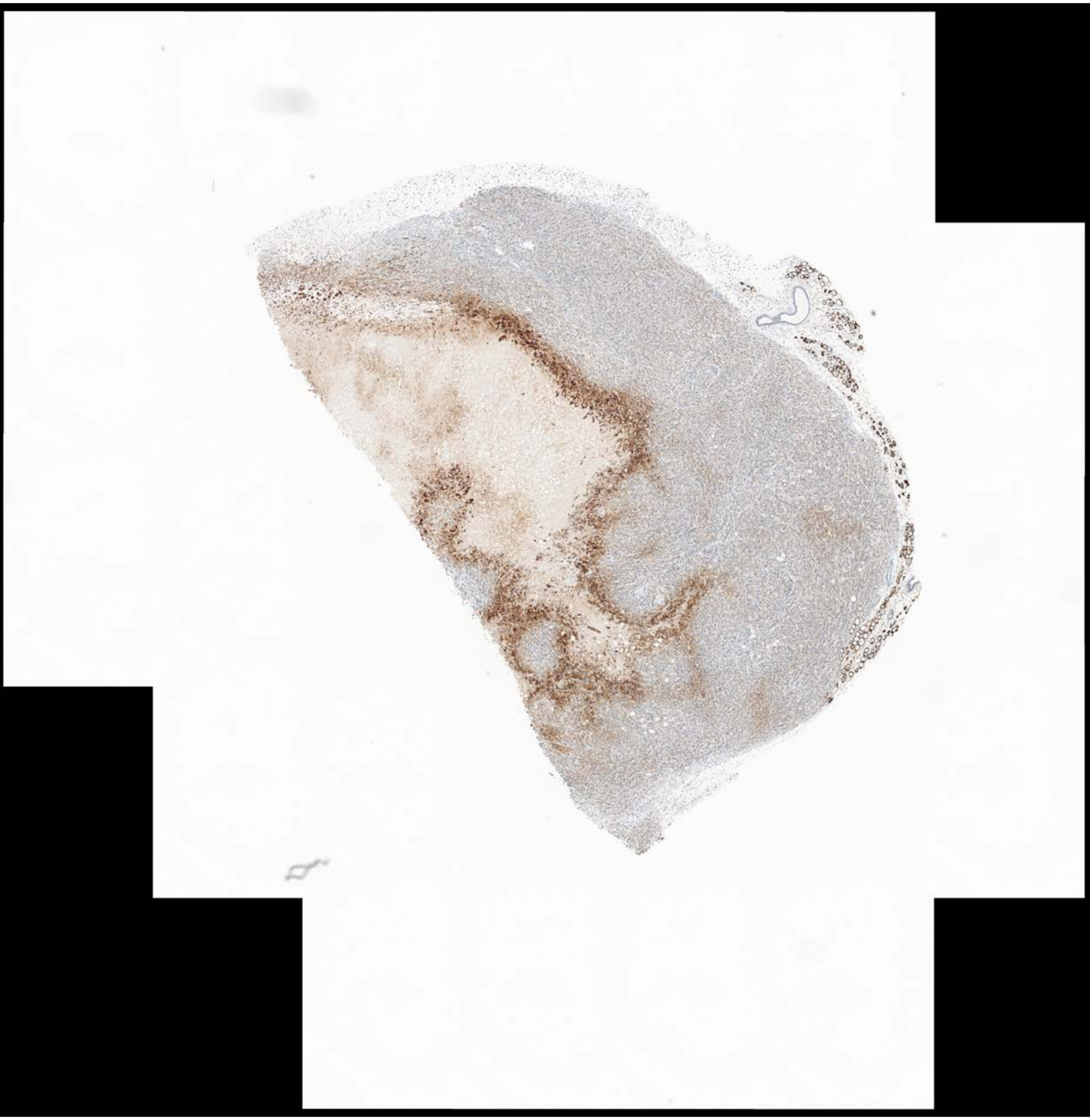

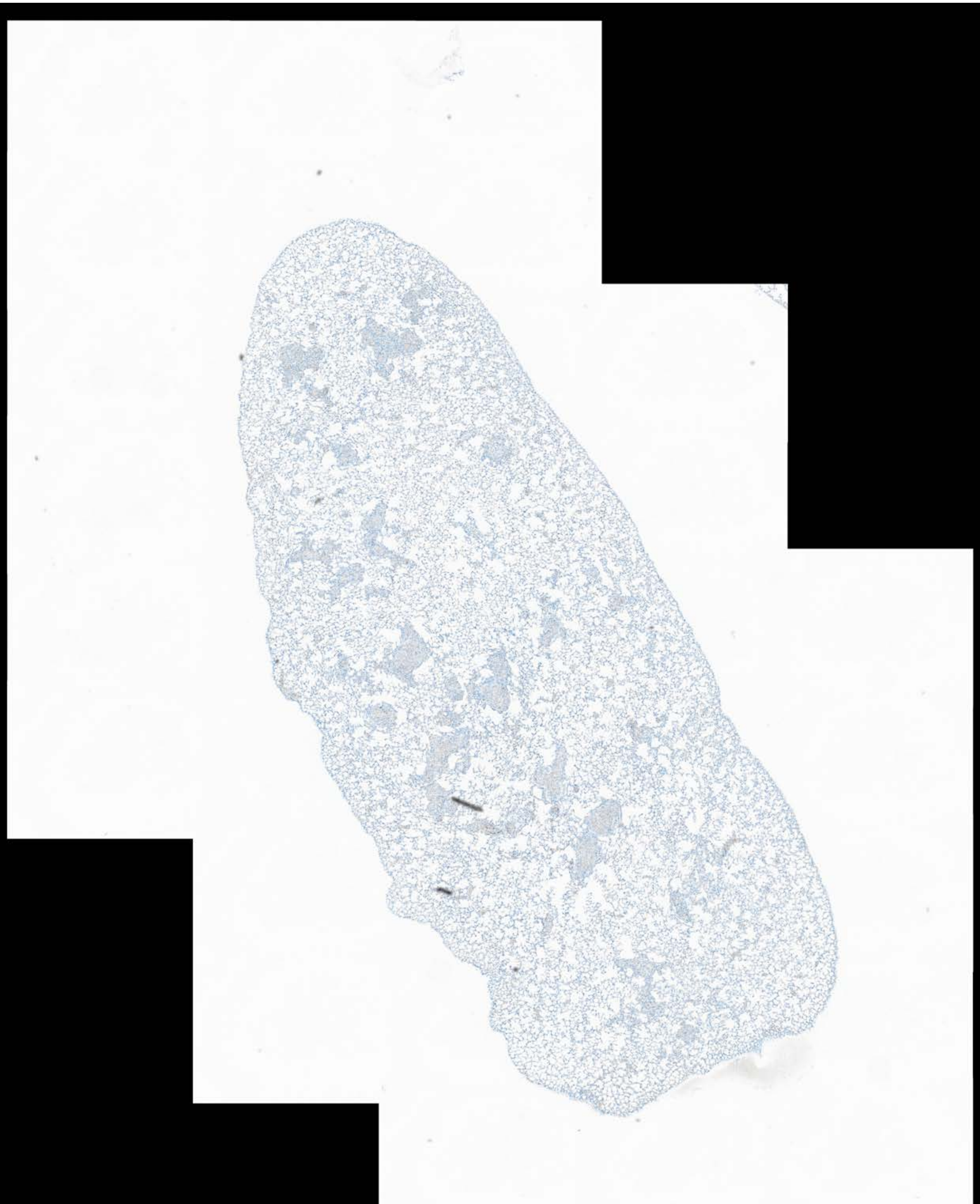

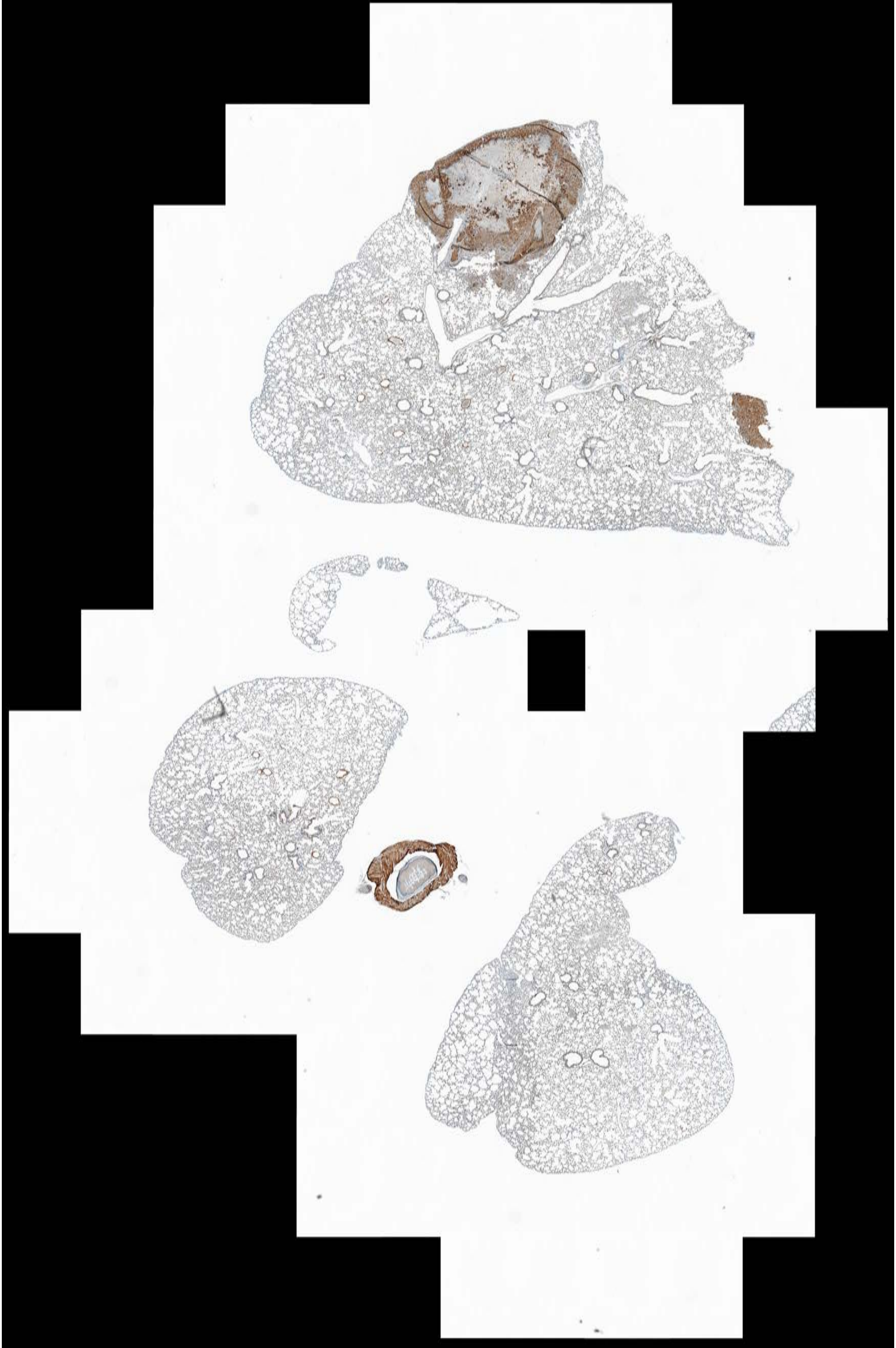

Supplement: Supplementary file 14 — Source Data for Figure 6 [file SourceDataFor_Figure6_ERO1A.pdf › SourceDataFor_Figure6_ERO1A.pdf]
