# Supplementary material for: The NFIB‐ERO1A axis promotes breast cancer metastatic colonization of disseminated tumour cells
Source: EMBO Mol Med. 2021 Mar 10;13(4):e13162. doi: 10.15252/emmm.202013162 (PMC8033524; doi:10.15252/emmm.202013162)
Supplement: Supplementary file 7 — Source Data for Expanded View and Appendix [file EMMM-13-e13162-s013.zip › SourceDataForAppendixFigures.zip!/SourceDataForAppendix FigureS1.zip!/SourceDataForAppendix FigureS1A.pdf]

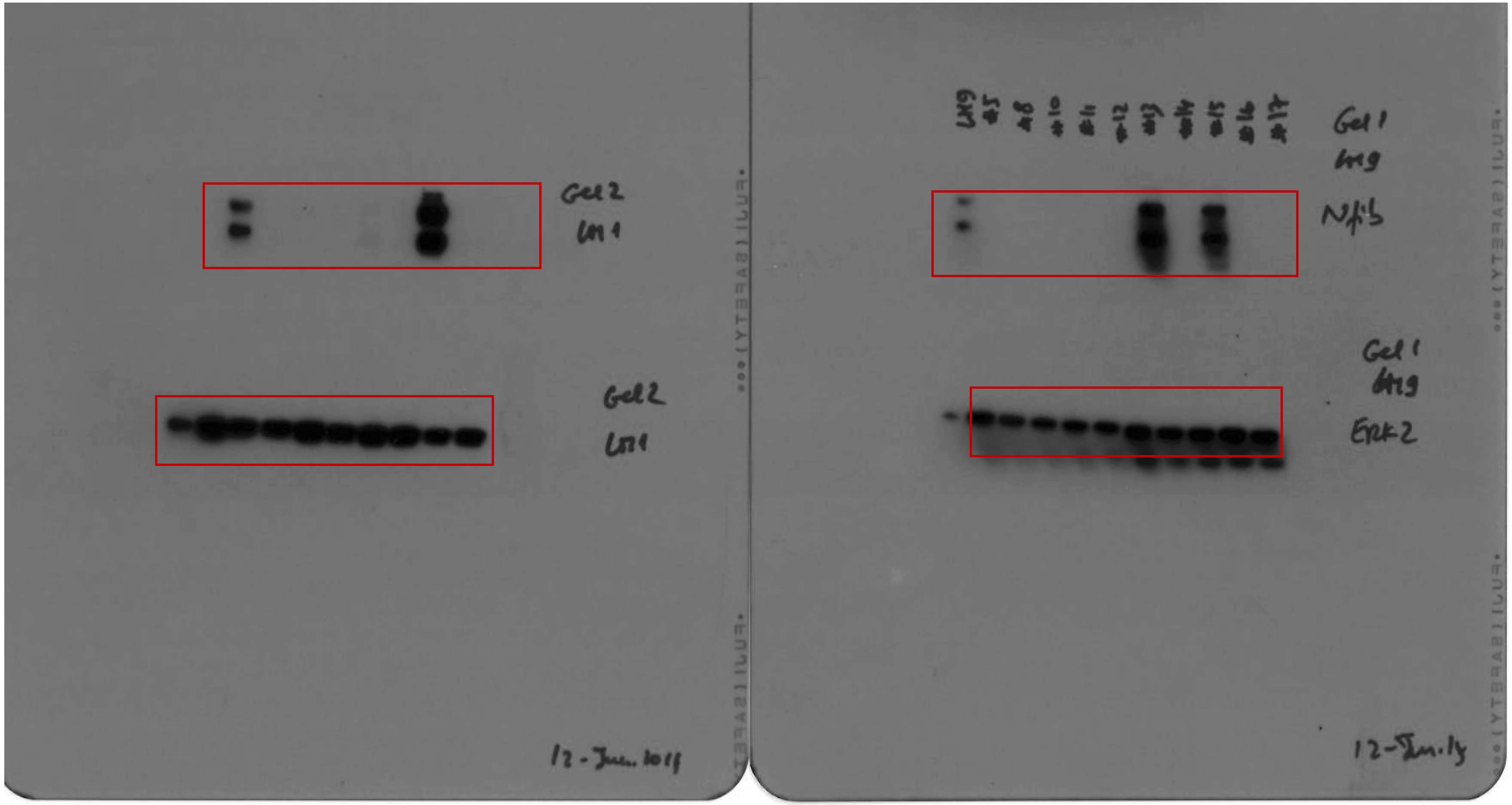
Gel 2
Gel 2
Gel 1
Nfib
Gel 1
ERK2
