# Supplementary material for: The NFIB‐ERO1A axis promotes breast cancer metastatic colonization of disseminated tumour cells
Source: EMBO Mol Med. 2021 Mar 10;13(4):e13162. doi: 10.15252/emmm.202013162 (PMC8033524; doi:10.15252/emmm.202013162)
Supplement: Supplementary file 7 — Source Data for Expanded View and Appendix [file EMMM-13-e13162-s013.zip › SourceDataForAppendixFigures.zip!/SourceDataForAppendix FigureS1.zip!/SourceDataForAppendix FigureS1B.pdf]

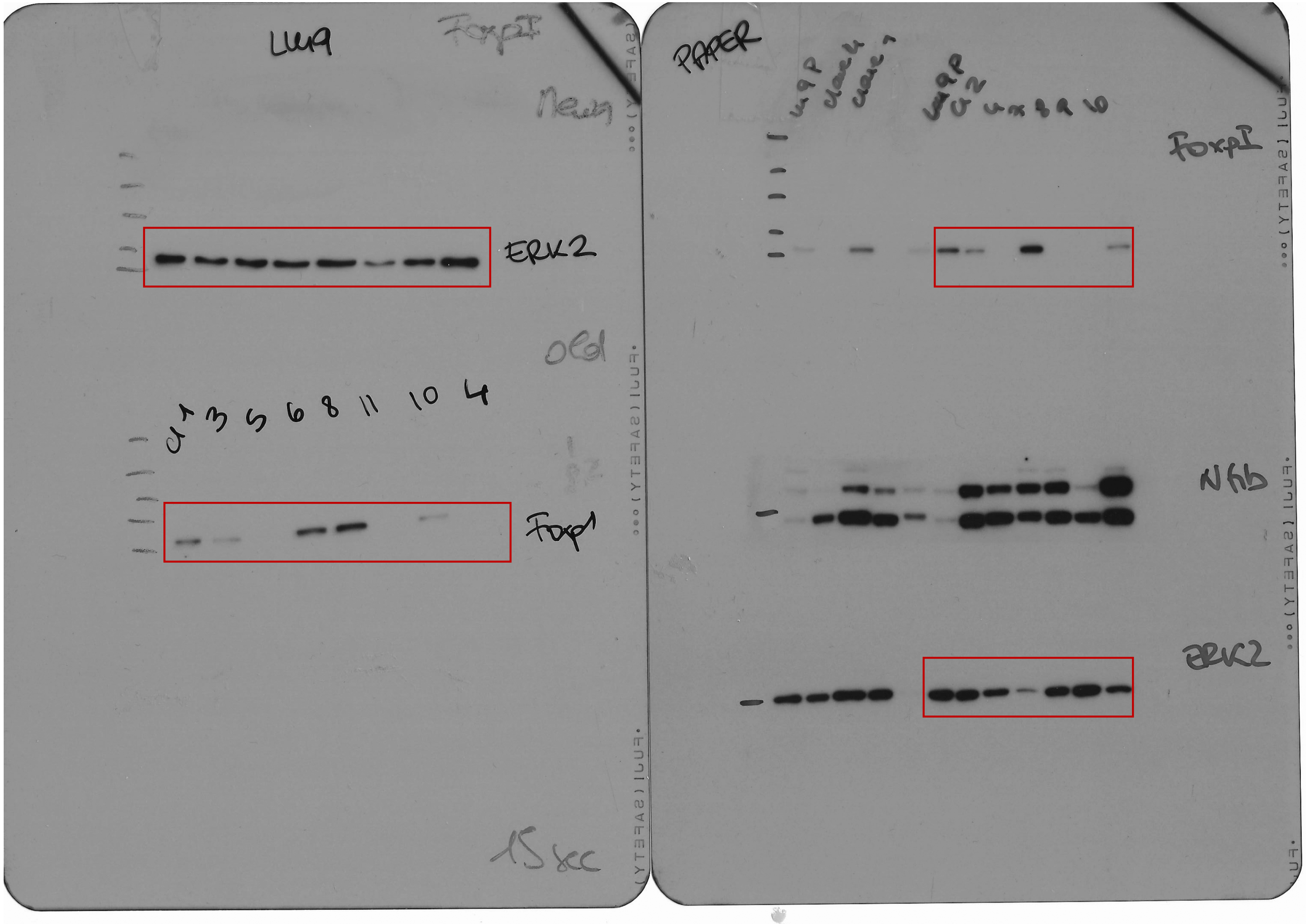

Lw9
FoxpI
New
ERK2
old
1 3 5 6 8 11 10 4
Foxp1
15 sec
PAPER
FoxpI
Nfkb
ERK2
